# Reactions of 2-carbonyl- and 2-hydroxy(or methoxy)alkyl-substituted benzimidazoles with arenes in the superacid CF_3_SO_3_H. NMR and DFT studies of dicationic electrophilic species

**DOI:** 10.3762/bjoc.15.191

**Published:** 2019-08-19

**Authors:** Dmitry S Ryabukhin, Alexey N Turdakov, Natalia S Soldatova, Mikhail O Kompanets, Alexander Yu Ivanov, Irina A Boyarskaya, Aleksander V Vasilyev

**Affiliations:** 1 Department of Chemistry, Saint Petersburg State Forest Technical University, Institutsky per., 5, Saint Petersburg, 194021, Russian Federation; 2 National Research Tomsk Polytechnic University, Lenin Avenue 30, Tomsk, 634050, Russian Federation; 3 Litvinenko Institute of Physico-Organic and Coal Chemistry, NAS of Ukraine, Kharkivske Hgw, 50, Kyiv, 02160, Ukraine; 4 National Technical University of Ukraine “Igor Sikorsky Kyiv Polytechnic Institute”, Prosp. Peremohy, 37, Kyiv, 03056, Ukraine; 5 Center for Magnetic Resonance, Research Park, Saint Petersburg State University, Universitetskiy pr., 26, Saint Petersburg, Petrodvoretz, 198504, Russian Federation; 6 Department of Organic Chemistry, Institute of Chemistry, Saint Petersburg State University, Universitetskaya nab., 7/9, Saint Petersburg, 199034, Russian Federation

**Keywords:** benzimidazoles, cations, Friedel–Crafts reaction, triflic acid

## Abstract

Reactions of 2-carbonyl- and 2-hydroxy(or methoxy)alkylbenzimidazoles with arenes in the Brønsted superacid TfOH resulted in the formation of the corresponding Friedel–Crafts reaction products, 2-diarylmethyl and 2-arylmethyl-substituted benzimidazoles, in yields up to 90%. The reaction intermediates, protonated species derived from starting benzimidazoles in TfOH, were thoroughly studied by means of NMR and DFT calculations and plausible reaction mechanisms are discussed.

## Introduction

Imidazoles and benzimidazoles are important heterocyclic scaffolds in pharmaceuticals and agrochemicals [1–10]. They also have applications in the fields of dyes, chemo-sensing, and fluorescent materials [3]. (Benz)imidazoles are a common motif present in some components of human organisms, histidine, vitamin B12, purines, histamine, biotin, and in natural compounds such as lepidiline A and B [6].

**Figure 1:**
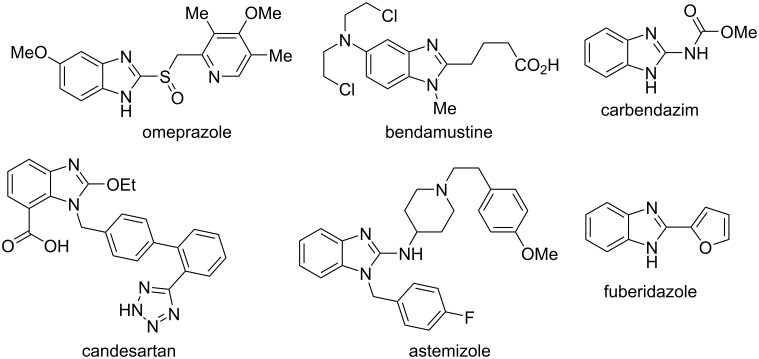
Examples of some commercially available pharmaceuticals and agrochemicals containing the benzimidazole scaffold.

Over the years of active research, benzimidazole derivatives have been involved in medicinal chemistry covering a wide range of biological activities including antiparasitic (albendazole, mebendazole), antiulcer (omeprazole), antihypertensive (candesartan, telmisartan, azilsartan, medoxomil, mebefradil), anticancer (bendamustine), antiemetic/antipsychotic (droperidole), antihistaminic (astemizole, emedastine) and many others (Figure 1) [1–10]. Benzimidazole fungicides (carbendazim, benomyl, thiabendazole and fuberidazole) have been widely used to fight against destructive plant pathogens (Figure 1) [7]. Interestingly, most of the above listed drugs are 2- or 1,2-disubstituted benzimidazole derivatives [2].

Thus, the development of further syntheses of benzimidazole derivatives and the study of their properties are important goals for chemistry, medicine and materials science.

Since George Olah proposed the concept of generating superelectrophilic intermediates through the protonation by Brønsted superacids or coordination by a Lewis superacid to produce a di- (tri- or higher) cationic species, the study of superelectrophilic activation became a very active area of research [11–13]. The acid-catalyzed condensation of ketones and aldehydes with aromatic compounds is known as the hydroxyalkylation reaction [14]. Recently, several hydroxyalkylation reactions followed by alkylation of arenes have been reported involving heterocycle-based superelectrophiles: pyridines, thiazoles, quinolines, isoquinolines, pyrazines, pyrazoles, imidazole and furans, bearing a formyl (carbonyl) group [15–23]. These carbonyl-substituted heteroarenes possess basic sites (nitrogen or oxygen atoms of the heterocyclic system), which are fully protonated in acid, so that upon subsequent protonation of the carbonyl oxygen, more reactive dicationic electrophiles can be generated.

**Figure 2:**
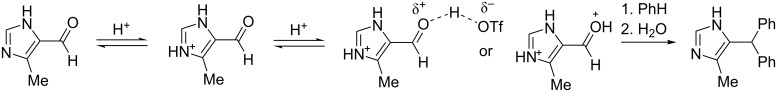
Formation of cationic species by protonation of 5-formyl-4-methylimidazole in TfOH and their reaction with benzene (data from ref. [19]).

Previously, superelectrophilic activation of the carbonyl group was achieved for 5-formyl and 5-acetylimidazoles in triflic acid CF_3_SO_3_H (TfOH) by Klumpp [19]. It was proposed that the triflic acid initially protonated the imidazole ring and an equilibrium was established with the O-protosolvated form or the dicationic superelectrophile. These two kinds of species may react with benzene leading to products of the transformation of the carbonyl group into a diphenylmethyl one (Figure 2) [19].

To the best of our knowledge, no data on the generation of superelectrophilic species from carbonyl-substituted benzimidazoles and their reactions exist. Based on our previous studies on the chemistry of heterocycles under superelectrophilic activation [22–24], we undertook this research on reactions of benzimidazole derivatives in (super)acids.

**Figure 3:**
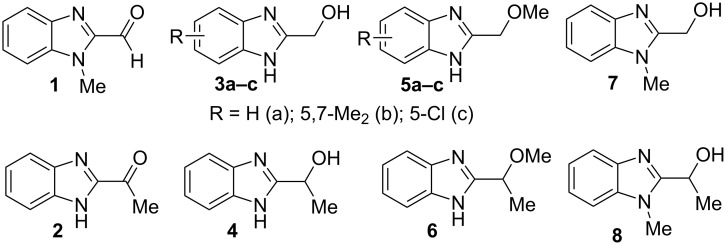
Benzimidazoles **1–8** used in this study.

The main goal of this work was the study of reactions of substituted benzimidazoles **1–8** (Figure 3) with arenes under the action of Brønsted (super)acids CF_3_SO_3_H (TfOH, triflic acid), H_2_SO_4_ and strong Lewis acids AlX_3_ (X = Cl, Br). One would expect the electrophilic activation of carbonyl or 2-hydroxyalkyl groups of these benzimidazoles in hydroxyalkylation and alkylation of arenes.

## Results and Discussion

The protonation of formyl and acetylbenzimadazoles **1** and **2** gave *N*,*O*-diprotonated species **I** and **II**, respectively (see Table 1). Protonation of the hydroxy group of benzimidazoles **3–8** in strong acids gave dicationic species **III**, **V**, **VII**, and **VIII**, the dehydration of the latter resulted in the formation of heteroaromatic benzyl-type dications **IV**, **VI**, and **IX**, respectively. Electronic characteristics, energies of HOMO/LUMO, electrophilicity indices ω [25–26], charge distribution, and contribution of atomic orbital into the LUMO of species **I–IX** were calculated by DFT method to estimate their electrophilicity and electronic properties (Table 1). Apart from that, Δ*G*_298_ of the formations of cations **I–IX** from parent benzimidazoles **1–8** were calculated to estimate a thermodynamic possibility of the species formation (Table 1). The calculations were carried out for the solution phase in water.

**Table 1:** Selected calculated [DFT, 6-311+G(2d,2p) basis set] electronic characteristics of the protonated forms of benzimidazoles.

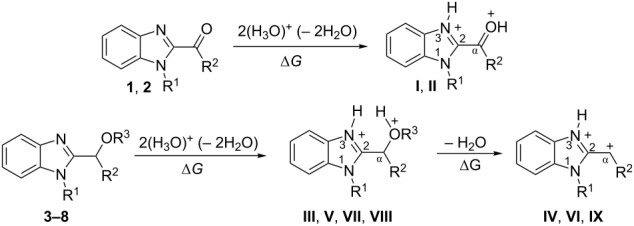										

Species	*E*_HOMO_, eV	*E*_LUMO_, eV	ω^a^ eV	q(C^α^)^b^ e	q(C2)^b^ e	q(N1)^b^ e	q(N3)^b^ e	k(Cα)_LUMO_^с^ %	k(C2)_LUMO_^с^ %	∆*G*^d^ kcal/mol

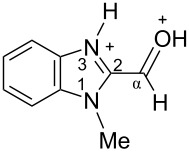 **I**	−9.53	−6.39	4.9	0.47	0.33	−0.29	−0.44	22.1	2.3	−18.6
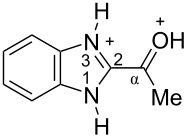 **II**	−9.38	−4.16	4.4	0.65	0.35	−0.30	−0.44	35.4	4.7	−21
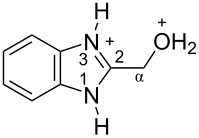 **III**	−9.11	−2.02	2.2	−0.06	0.46	−0.47	−0.48	17.3	26.3	−21.5
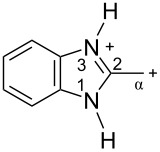 **IV**	−10.07	−5.23	6.1	−0.06	0.31	−0.42	−0.42	28.2	0.6	15.8
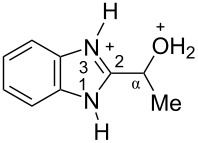 **V**	−9.03	−1.85	2.1	0.11	0.48	−0.34	−0.49	13.9	20.4	−25.6
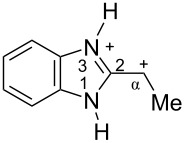 **VI**	−9.77	−4.92	5.6	0.14	0.33	−0.28	−0.43	30.5	1.4	7.6
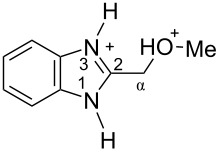 **VII**	−9.09	−1.90	2.1	−0.06	0.47	−0.48	−0.47	16.3	22.6	−25.8
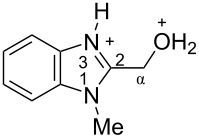 **VIII**	−9.04	−2.01	2.2	−0.06	0.47	−0.34	−0.48	22.1	17.3	−23.7
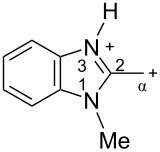 **IX**	−9.96	−5.14	5.9	−0.08	0.33	−0.29	−0.43	26.5	0.6	14.4

^a^Global electrophilicity index ω = (*E*_HOMO_ + *E*_LUMO_)^2^/8(*E*_LUMO_ − *E*_HOMO_). ^b^Natural charges. ^c^Contribution of atomic orbital into the molecular orbital. ^d^Gibbs energy of the species formation reaction.

According to DFT calculations, protonation of the benzimidazole nitrogen N3 and the oxygen of the carbonyl or hydroxymethyl group in **1–8** leading to dicationic species **I–III**, **V**, **VII**, and **VIII** is thermodynamically favorable (−18.6 to −25.8 kcal/mol, Table 1). On the other hand, the dehydration of species **III**, **V**, **VII**, **VIII** is rather unfavorable, since Δ*G*_298_ values for the formation of dications **IV**, **VI**, and **IX** are 7.2–15.8 kcal/mol (Table 1). These data reveal that, in the case of carbonyl-substituted benzimidazoles **1** and **2**, the formation of dications **I** and **II** is very likely, and these species should be reactive electrophiles. While, in the case of hydroxyalkylbenzimidazoles **3–8**, *N,O*-diprotonated species **III**, **V**, **VII**, **VIII**, the most probably, may be reactive intermediates.

The calculation of electrophilic properties of these cations show that species **I** and **II** have higher values of electrophilicity indices ω of 4.4 and 4.9 eV, respectively, compared to cations **III**, **V**, **VII**, and **VIII** with ω values of 2.1–2.2 eV. Among all studied species, the heteroaromatic benzyl dication **IV**, **VI**, **IX** have the highest electrophilicity indices ω = 5.6–6.1 eV (Table 1).

In the dications **I** and **II**, the carbon Cα bears a large positive charge (0.47 and 0.65 e) and gives a big contribution into the LUMO (22.1 and 35.4%, Table 1). It points out that this particular carbon should be an electrophilic reactive center by both charge and orbital factors. For comparison, the carbon C2 in **I** and **II** has a less positive charge (0.33 and 0.35 e) and a much less contribution in the LUMO (2.3 and 4.7%).

In hydroxonium type species **III**, **V**, **VII**, and **VIII**, the charge on the carbon Cα is from −0.06 to 0.11 e (Table 1). However, this carbon provides a big contribution into the LUMO, 13.9–22%. This reveals that electrophilic properties of this carbon are mainly ruled by orbital factors. Despite the carbon C2 in these species has a large positive charge (0.46–0.48 e) and a great orbital coefficient (17.3–26.3%), reactions of nucleophiles with this atom are less probable, since it leads to a loss of aromaticity of the benzimidazole system. The same applies to the reactivity of the carbon C2 in dications **I** and **II**.

Then we studied the protonation of benzimidazoles in the superacid TfOH by means of NMR. Selected ^1^H, ^13^C and ^15^N NMR data for starting neutral benzimidazoles **1**, **3a**, and **7** (in CDCl_3_, (CD_3_)_2_CO, CD_3_OD) and their corresponding protonated forms **I'**, **III'**, and **VIII'**, respectively, in TfOH are presented in Table 2.

**Table 2:** ^1^H, ^13^C and ^15^N NMR data of benzimidazoles **1**, **3a**, **7** in the corresponding deuterated solvent and species **I'**, **III'**, **VIII'** in TfOH at room temperature.

Compound or cation	Solvent	NMR, δ, ppm					
		^1^H		^13^C		^15^N	
		Hα	N3H	C2	Cα	N1	N3

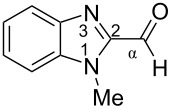 **1**	CDCl_3_	10.09	–	146.1	185.0	268.4	142.2
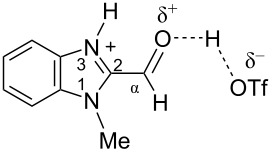 **I‘**	TfOH	10.28	12.12	137.4	176.8	158.9	151.6

∆δ^a^		0.19		−8.7	−8.2	109.5	9.4

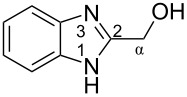 **3a**	(CD_3_)_2_CO	4.86	–	155.8	59.2	–^b^	–^b^
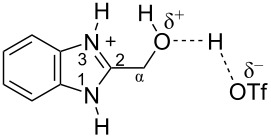 **III‘**	TfOH	5.64	11.53	149.5	57.5	148.7	148.7

∆δ^a^		0.78		−6.3	−1.7	–	–

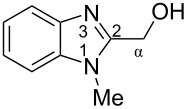 **7**	CD_3_OD	4.85	–	142.5	57.7	no data	no data
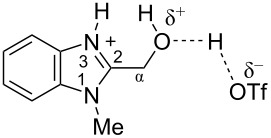 **VIII‘**	TfOH	5.57	11.37	148.4	56.9	152.5	149.1

∆δ^a^		0.72		5.9	−0.8	–^b^	–^b^

^a^∆δ = δ_cation_ − δ_initial_. ^b^No correlation was observed in HSQC and HMBC N–H spectra.

The signals of the proton attached to the nitrogen N3 in ^1^H NMR spectra of all species **I'**, **III'**, and **VIII'** in TfOH appear in the range of 11.37–12.12 ppm (Table 2, see also the spectra given in Supporting Information File 1). However, the signals of the proton bound to the oxygen of the formyl or the hydroxy groups is not observed due to the fast proton exchange for these groups in TfOH at room temperature.

The obtained NMR data demonstrate the formation of *N*-protonated-*O*-protosolvated species **I'**, **III'**, and **VIII'** from benzimidazoles **1**, **3a**, and **7**, respectively, in the superacid TfOH. However, it could not be excluded that the *N*,*O*-diprotonated species was formed in low concentration as a result of complete protonation of oxygen. These dicationic species may participate in further reactions with arenes.

Signals of Hα protons for species **I'**, **III'**, and **VIII'** in TfOH are downfield shifted relatively to the same signals in their neutral precursors **1**, **3a**, and **7** (in CDCl_3_, (CD_3_)_2_CO, CD_3_OD), respectively (see ∆δ values in Table 2). This reveals substantial solvatation of the formyl or the hydroxy oxygen of benzimidazoles **1**, **3a**, **7** in TfOH.

In the ^13^C NMR spectra, the signal of carbon Cα in species **I'**, **III'**, and **VIII'** in TfOH is slightly upfield shifted due to positive charge delocalization into the benzimidazole ring. Apart from that, the chemical shift of Cα in hydroxonium-type species **III'** and **VIII'** at 57.5 and 56.9 ppm, which are close to the shifts in starting neutral precursors **3a** and **7**, proves unambiguously that no dehydration of these species leading to heteroaromatic benzyl-type cations take place.

Based on HSQC and HMBC N–H correlations, we were able to measure ^15^N chemical shifts of nitrogen atoms for cations **I'**, **III'**, and **VIII'** in TfOH (Table 2). The peaks of the nitrogen atoms N1 and N3 appear in a rather narrow range of 148.7–158.9 ppm due to charge delocalization between these two nitrogen atoms for the corresponding resonance forms.

Then, we carried out Friedel–Crafts reactions of benzimidazoles **1–8** with arenes under the action of Brønsted acids (H_2_SO_4_, TfOH) or Lewis acids (AlX_3_, X = Cl, Br). Reactions of 2-formyl-1-methylbenzimidazole (**1**) with various arenes (benzene, and its methyl, methoxy or chloro-substituted derivatives) are given in Table 3. These reactions proceed on the formyl group of **1** and give rise to the corresponding 2-diarylmethylbenzimidazoles **9a–l**. The best results were obtained in neat TfOH, which gave high yields of reaction products (54–86%) at room temperature after 2–3 h. Other acids were not so efficient. Thus, reactions under the action of sulfuric acid H_2_SO_4_ or AlCl_3_ and AlBr_3_ took longer time (24–27 h, Table 3, entries 1, 2, and 4). In some cases, the use of H_2_SO_4_ resulted in the formation of oligomeric reaction products in the reaction of **1** with electron donating *p*-xylene (Table 3, entry 10). Apart from that, the acidity of H_2_SO_4_ was not high enough to activate **1** in the reaction with poor π-nucleophilic 1,2-dichlorobenzene (Table 3, entry 13).

**Table 3:** Reactions of 2-formyl-1-methylbenzimidazole (**1**) with arenes under the action of various acids at room temperature.

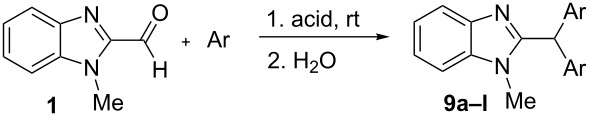				

Entry	Arene (equiv)	Reaction conditions^a^		Reaction products **9**, yield (%)
		Acid (equiv)	Time, h	

1	benzene (18)	H_2_SO_4_ (120)	24	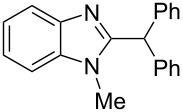 **9a** (69%)
2	benzene (18)	TfOH (35)	2	**9a** (61%)
3	benzene (100)	AlCl_3_ (5)	25	**9a** (54%)^b^
4	benzene (100)	AlBr_3_ (5)	27	**9a** (80%)
5	toluene (2.2)	TfOH (35)	2	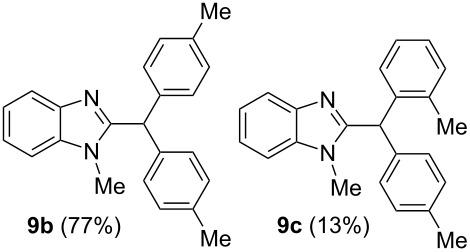 ratio **9b**/**9c** 6:1
6	anisole (2.2)	TfOH (35)	2	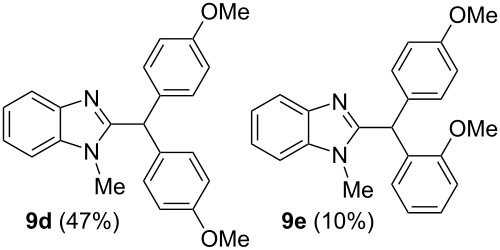 ratio **9d**/**9e** 5:1
7	chlorobenzene (2.2)	TfOH (35)	2	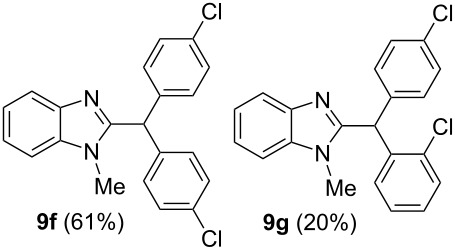 ratio **9f**/**9g** 3:1
8	*o*-xylene (2.2)	TfOH (35)	3	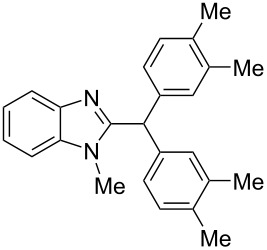 **9h** (75%)
9	*m*-xylene (2.2)	TfOH (35)	3	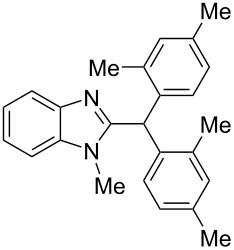 **9i** (63%)
10	*p*-xylene (2.2)	H_2_SO_4_ (120)	24	oligomeric materials
11	*p*-xylene (2.2)	TfOH (35)	3	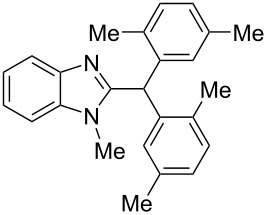 **9j** (86%)
12	1,2-dichlorobenzene (2.2)	TfOH (35)	3	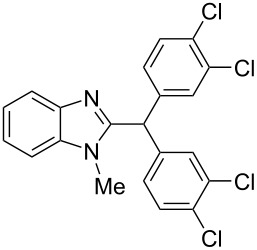 **9k** (61%)
13	1,2-dichlorobenzene (2.2)	H_2_SO_4_ (120)	24	–^c^
14	1,2,4-tremethylbenzene (2.2)	TfOH (35)	3	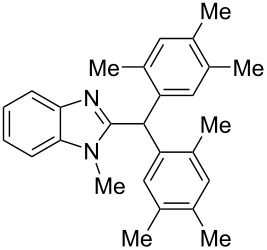 **9l** (78%)

^a^All reactions were carried out at room temperature. ^b^26% of the starting benzimidazole **1** was recovered. ^c^72% of the starting benzimidazole **1** was recovered.

**Scheme 1:**
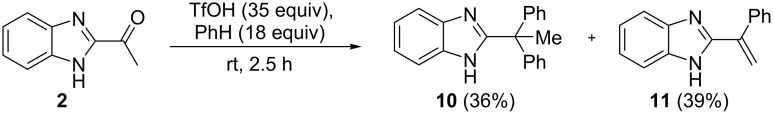
Reaction of 2-acetylbenzimidazole (**2**) with TfOH and benzene.

2-Acetylbenzimidazole (**2**) reacted with benzene in TfOH in the same way, which gave two reaction products **10** and **11** (Scheme 1). Compound **10** was obtained as a result of the addition of two benzene molecules to the carbonyl group of **2**. Alkenyl-substituted benzimidazole **11** was formed in an alternative way of transformation of intermediate cations (see reaction mechanism below in Scheme 3). It should be noted, that compound **11** in the reaction with benzene in TfOH at room temperature for 24 h afforded benzimidazole **10**.

**Scheme 2:**
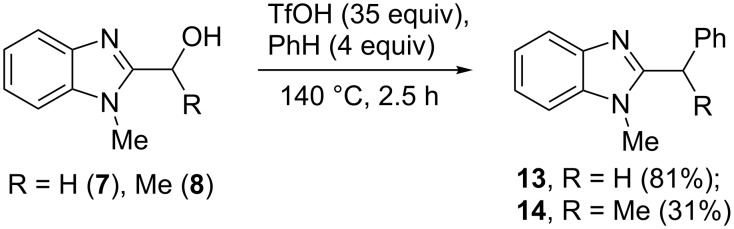
Reactions of hydroxymethyl-substituted benzimidazole **7** and **8** with TfOH and benzene.

Then, we studied reactions of 2-hydroxyalkylbenzimidazoles **3a–c**, **4**, **7**, and **8**. It was found that these reactions needed extremely harsh reaction conditions, heating in neat TfOH at 140 °C in glass high pressure tubes (Table 4, Scheme 2). Only at this high temperature the formation of Friedel–Crafts reaction products **12a–h**, **13**, and **14** was achieved. No reactions took place at lower temperature or under the action of Lewis acids AlX_3_ (X = Cl, Br). For these 2-hydroxyalkylbenzimidazoles **3a–c**, **4**, **7**, and **8**, we were able to receive compounds **12–14** only in reactions with benzene, 1,2-dichlorobenzene and 1,3-dibromobenzene (Table 4). Transformations with methyl-substituted benzenes (xylenes) at these harsh conditions gave complex mixtures of oligomeric materials. The same interactions of 2-methoxyalkylbenzimidazoles **5a–c**, and **6** with benzene in TfOH at 140 °C resulted in the formation of compounds **12a**,**f–h** (Table 5).

**Table 4:** Reactions of 2-hydroxyalkylbenzimidazoles **3a–c** and **4** with arenes in TfOH at 140 °C.

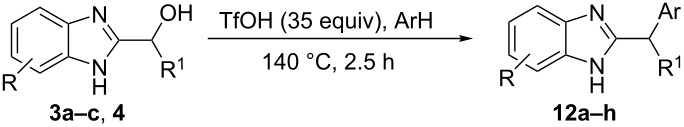			

Entry	Starting benzimidazole	Starting arene (equiv)	Reaction products **12**, yield (%)

1	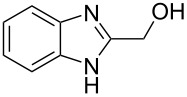 **3a**	benzene (18)	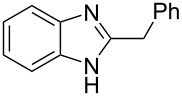 **12a** (87%)
2	**3a**	1,2-dichlorobenzene (4)	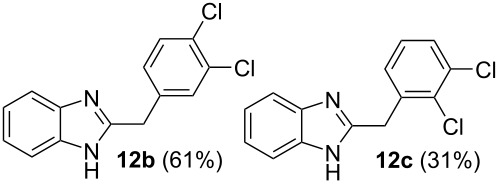 ratio **12b**/**12c** 1:0.5
3	**3a**	1,3-dibromobenzene (4)	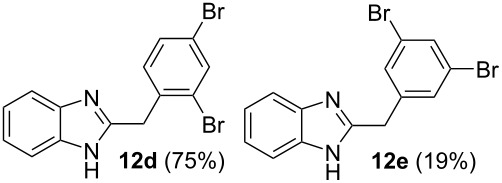 ratio **12d**/**12e** 1:0.25
4	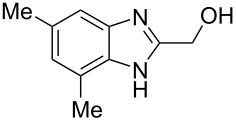 **3b**	benzene (18)	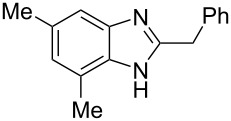 **12f** (82%)
5	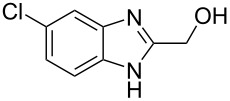 **3c**	benzene (18)	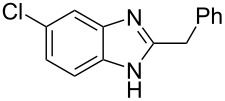 **12g** (88%)
6	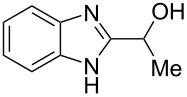 **4**	benzene (18)	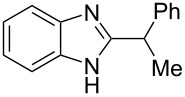 **12h** (90%)

**Table 5:** Reactions of 2-methoxyalkylbenzimidazoles **5a–c**, and **6** with benzene in TfOH at 140 °C.

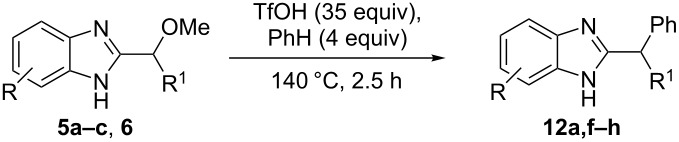		

Entry	Strarting benzimidazole	Reaction products **12**, yield (%)

1	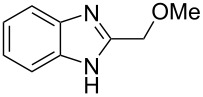 **5a**	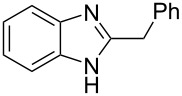 **12a** (79%)
2	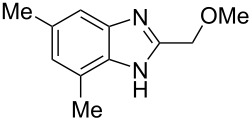 **5b**	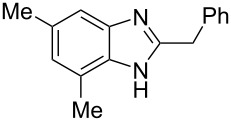 **12f** (79%)
3	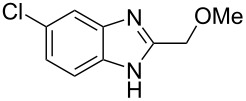 **5c**	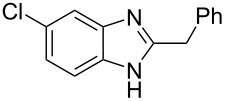 **12g** (52%)
4	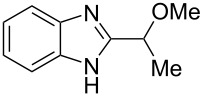 **6**	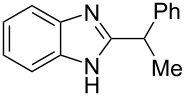 **12h** (84%)

**Scheme 3:**
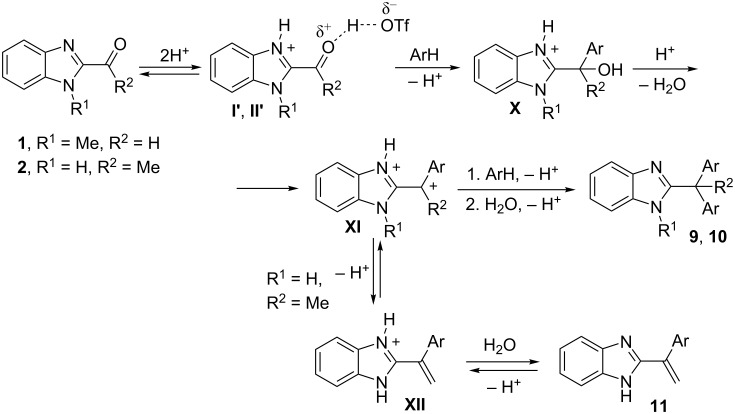
Reaction mechanism of the formation of compounds **9–11**.

**Scheme 4:**
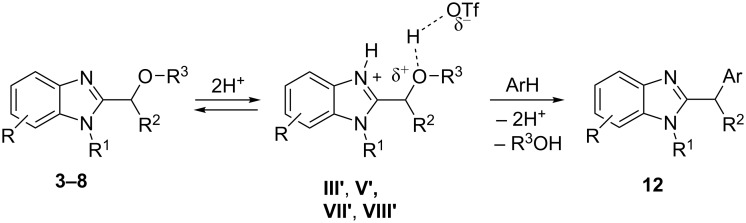
Reaction mechanism of the formation of compounds **12**.

Summarizing all data obtained by DFT calculations (Table 1) and NMR studies (Table 2) of intermediate cations generated from benzimidazoles **1–8** in TfOH, and their reactions with arenes (Tables 3–5, Scheme 1, and Scheme 2), the following reaction mechanisms are proposed (Scheme 3 and Scheme 4).

2-Carbonyl-substituted benzimidazoles **1** and **2** give rise to *N*-protonated-*O*-protosolvated forms **I'** and **II'**. These species in reactions with arenes furnish hydroxyalkylation products **X**, which are further transformed into cations **XI**. These species consequently react with arenes forming the target compounds **9** and **10** after final hydrolysis of superacidic reaction mixtures. There is another possible reaction pathway for 2-acetylbenzimidazole (**2**) on the stage of the formation of cation **XI**. The latter may undergo deprotonation at the methyl group, which results in the formation of alkenyl benzimidazole **11**. Compound **11** may be also transformed into compound **10** by reaction with arenes in TfOH (see Scheme 2 and the corresponding discussion).

Hydroxy(or methoxy)alkyl-substituted benzimidazoles **3–8** form cations **III'**, **V'**, **VII'**, and **VIII'**, respectively. These species react with arenes, the most probably, in S_N_2 way to give benzimidazoles **12** (Scheme 4).

However, once again, in these reactions (Scheme 3 and Scheme 4) the formation of the corresponding *N*,*O*-diprotonated species **I'**, **II'**, **III'**, **V'**, **VII'**, and **VIII'** in low concentration, which are not detected by NMR, could not be excluded. And these dications may react with arenes, as well.

It should be specially emphasized that 2-diarylmethyl and 2-arylmethyl-substituted benzimidazoles **9–12** are synthetically hardly available compounds [27–28]. For instance, they were investigated as AMP-activated protein kinase activators [27].

## Conclusion

Reactions of 2-carbonyl and 2-hydroxy(or methoxy)alkyl-substituted benzimidazoles with arenes in the Brønsted superacid TfOH were studied for the first time. These reactions proceed at the carbon atom of the 2-carbonyl or 2-hydroxyalkyl group leading to the corresponding 2-diarylmethyl or 2-arylmethyl-substituted benzimidazoles. Based on this superelectrophilic activation approach, we have developed a useful synthetically procedure for the modification of the benzimidazole structure.

## Supporting Information

File 1 Experimental details, compound characterization data, copies of ^1^H and ^13^C NMR spectra, and details of DFT calculations.
