# Supplementary material for: Reactions of 2-carbonyl- and 2-hydroxy(or methoxy)alkyl-substituted benzimidazoles with arenes in the superacid CF3SO3H. NMR and DFT studies of dicationic electrophilic species
Source: Beilstein J Org Chem. 2019 Aug 19;15:1962–73. doi: 10.3762/bjoc.15.191 (PMC6720581; doi:10.3762/bjoc.15.191)
Supplement: File 1 — Experimental details, compound characterization data, copies of 1H and 13C NMR spectra, and details of DFT calculations. [file Beilstein_J_Org_Chem-15-1962-s001.pdf]

BEILSTEIN JOURNAL OF ORGANIC CHEMISTRY

Supporting Information

for

Reactions of 2-carbonyl- and 2-hydroxy(or methoxy)alkyl-substituted benzimidazoles with arenes in the superacid CF_3SO_3H. NMR and DFT studies of dicationic electrophilic species

Dmitry S. Ryabukhin, Alexey N. Turdakov, Natalia S. Soldatova, Mikhail O. Kompanets, Alexander Yu. Ivanov, Irina A. Boyarskaya and Aleksander V. Vasilyev

Beilstein J. Org. Chem. **2019,** *15,* 1962–1973. doi:10.3762/bjoc.15.191

Experimental details, compound characterization data, copies of ^{1}H and ^{13}C NMR spectra, and details of DFT calculations

License and Terms: This is a supporting information file under the terms of the Creative Commons Attribution License (http://creativecommons.org/licenses/by/4.0). Please note that the reuse, redistribution and reproduction in particular requires that the authors and source are credited.
The license is subject to the *Beilstein Journal of Organic Chemistry* terms and conditions: (https://www.beilstein-journals.org/bjoc)

Contents

1. Experimental part…………………………………………………………………...S2

2. References………………………………………………………………………..S9

3. ^{1}H and ^{13}C NMR spectra of compounds **1–14**……………………………………….S10

4. ^{1}H and ^{13}C NMR spectra of cations **I, III, VIII** in TfOH……………………………..S40

5. Details of DFT calculations of cations **I–IX**…………………………………………...S47

1. Experimental part

General

NMR spectra of solutions of compounds in $CDCl_3$, MeOD-d$_4$, $(CD_3)_2CO$ were recorded on Bruker AVANCE 500 spectrometer (at 500 and 125 MHz for ^{1}H and ^{13}C NMR spectra respectively) at 25 °C. The solvent residual signals $CDCl_3$ (δ 7.26 ppm), MeOD-d_4 (δ 3.31 ppm), $(CD_3)_2CO$ (δ 2.05 ppm) for ^{1}H NMR spectra and the carbon signal of $CDCl_3$ (δ 77.0 ppm), MeOD-d_4 (δ 49.0 ppm), $(CD_3)_2CO$ (δ 29.8 ppm) for ^{13}C NMR spectra were used as references. NMR spectra in TfOH were recorded on a Bruker AVANCE III 400 spectrometer (at 400 and 100 MHz for ^{1}H and ^{13}C NMR spectra, respectively) using CH_2Cl_2 as internal standard. HRMS was carried out with a Bruker maXis HRMS-ESI-QTOF instrument or by matrix-assisted laser desorption ionization (MALDI–MS) using Fourier Transform (Ion Cyclotron Resonance). The preparative reactions were monitored by thin-layer chromatography carried out on silica gel plates (Silufol UV-254), using UV light for detection. Column chromatography was performed on silica gel Merck-60 with petroleum ether/diethyl ether or DCM/MeOH mixtures as eluents.

DFT calculations. All computations were carried out at the DFT/HF hybrid level of theory using hybrid exchange functional M06 by using GAUSSIAN 2009 program packages [1]. The geometry optimizations were performed using the 6-311+G(2d,2p) basis set (standard 6-311 basis set added with polarization (d, p) and diffuse functions). Optimizations were performed on all degrees of freedom and solvent-phase optimized structures were verified as true minima with no imaginary frequencies. The Hessian matrix was calculated analytically for the optimized structures in order to prove the location of correct minima and to estimate the thermodynamic parameters. Solvent-phase calculations used the Polarizable Continuum Model (PCM).

Procedures for the synthesis of 2-carbonylbenzimidazoles 1-2. Analogous as described in [2].

Dess–Martin reagent (1.1 mmol) was added to a solution of benzimidazol-2-ylmethanol **4** or **7** (1 mmol) in methylene chloride (10 mL). The reaction mixture was stirred at 4 °C for 1 h prior to being quenched with saturated aqueous sodium thiosulfate solution (3 mL). The subsequent mixture was extracted with methylene chloride (3 × 10 mL). The combined organic extracts were dried over anhydrous magnesium sulfate and concentrated in vacuo to provide a crude product, which was crystallized from methanol to give corresponding product.

1-Methyl-1*H*-benzimidazole-2-carboxaldehyde (1) [3] was obtained as beige solid in 30% yield. M.p. 134-136°C from methanol. ^{1}H NMR (500 MHz, $CDCl_3$) δ 4.12 (s, NMe), 7.35-7.39 (m, 1$H_{arom.}$), 7.43-7.48 (m, 2$H_{arom.}$), 7.90 (d, 1$H_{arom.}$, J = 10.3 Hz), 10.09 (s, 1H, CHO). ^{13}C NMR (125 MHz, $CDCl_3$) δ 31.2, 110.5, 122.3, 124.0, 126.8, 136.9, 142.7, 146.1, 185.0.

2-Acetyl-1*H*-benzimidazole (2) [4] was obtained as beige solid in 30% yield. M.p. 134-136°C from methanol. ^{1}H NMR (500 MHz, $CDCl_3$) δ 2.81 (s, 3H, Me), 7.38 (br.s, $2H_{arom.}$), 7.68 38 (br.s, $2H_{arom.}$), 10.16 (br.s, 1H, NH). ^{13}C NMR (125 MHz, $CDCl_3$) δ 25.9, 141.7, 147.7, 191.8.

Procedures for synthesis of substituted benzimidazol-2-methanols 3a–c and 4.

Mixture of phenylenediamine 10 g (92.6 mmol), glycolic/lactic acid (166.7 mmol), 135 ml 4M HCl was refluxed for 6 hours. Then reaction mixture was alkalinized to pH 7 with aqueous solution of KOH. Precipitate was filtered and crystallized from water.

(Benzimidazol-2-yl)methanol (**3a**) [5] was obtained as beige solid in 74% yield. M.p. 159-161°C from H_2O. ^{1}H NMR (500 MHz, $(CD_3)_2CO$) δ 4.86 (s, 2H), 7.23 – 7.11 (m, $2H_{arom.}$), 7.64 – 7.46 (m, $2H_{arom.}$), 11.47 (brs, 1H). ^{13}C NMR (125 MHz, $(CD_3)_2CO$) δ 59.2, 122.4, 155.8. HRMS, m/z calculated for $C_8H_8N_2NaO$ [M+Na]: 171.0534. Found: 171.0537.

(5,7-Dimethylbenzimidazol-2-yl)methanol (**3b**) was obtained as beige solid in 54% yield. M.p. 173-175°C.^{1}H NMR (500 MHz, MeOD-d_4) δ 2.39 (s, 3H), 2.50 (s, 3H), 4.80 (s, 2H), 6.84 (s, $1H_{arom.}$), 7.14 (s, $1H_{arom.}$). ^{13}C NMR (125 MHz, MeOD-d_4) δ 17.0, 21.6, 58.9, 125.5, 133.3, 155.1. HRMS, m/z calculated for $C_{10}H_{13}N_2O$ $[M+H]^+$: 177.1022. Found: 177.1029.

(5-Chlorobenzimidazol-2-yl)methanol (**3c**) [6] was obtained as beige solid in 54% yield. M.p. 186-188°C. ^{1}H NMR (500 MHz, MeOD-d_4) δ 4.82 (s, 2H), 7.20 (dd, $1H_{arom.}$, J = 2.0, 8.6 Hz), 7.50 (d, $1H_{arom.}$, J = 8.6 Hz), 7.53 (s, $1H_{arom.}$). ^{13}C NMR (125 MHz, MeOD-d_4) δ 58.9, 123.8, 129.0, 157.8.

1-(1*H*-benzimidazol-2-yl)ethan-1-ol 4 [4] was obtained as beige solid in 93% yield. M.p. 172-174°C from ethanol. ^{1}H NMR (500 MHz, MeOD-d_4) δ 1.61 (d, 3H, J = 6.5 Hz), 5.06 (q, 1H, J = 6.5 Hz), 7.35 – 6.98 (m, 2H), 7.67-7.34 (m, 2H). ^{13}C NMR (125 MHz, MeOD-d_4) δ 23.1, 65.5, 111.1, 115.8, 123.3, 139.3, 159.8.

Procedures for synthesis of substituted 2-(methoxymethyl)-1*H*-benzimidazole 5a–c and 6.

NaOH (9.5 mmol) was added to a stirring solution of benzimidazole (6.7 mmol) in 10 mL of EtOH/H_2O 3:1 (v/v). Dimethyl sulfate (9.5 mmol) was added dropwise over 5 min and the mixture was stirred for additional 2 h. Solvents were evaporated to give colorless solid, which was crystallized from methanol to give corresponding product.

2-(Methoxymethyl)-1*H*-benzimidazole (5a) [7] was obtained as beige solid in 95% yield. M.p. 137-139°C from methanol.^{1}H NMR (500 MHz, MeOD-d_4) δ 3.67 (s, 3H, OMe), 5.10 (s, 2H), 7.55 (dd, $2H_{arom.}$, J = 3.1, 6.2 Hz), 7.76 (dd, $2H_{arom.}$, J = 3.1, 6.2 Hz). ^{13}C NMR (125 MHz, MeOD-d_4) δ 55.1, 56.7, 114.9, 127.2, 132.5, 156.0. HRMS, m/z calculated for $C_9H_{11}N_2O$ $[M+H]^+$: 163.0866. Found: 163.0873.

5,7-Dimethyl-2-(methoxymethyl)-1*H*-benzimidazole (5b) was obtained as beige solid in 94% yield. M.p. 110-112°C from methanol. ^{1}H NMR (500 MHz, MeOD-d_4) δ 3.68 (s, 3H, OMe), 4.99 (s, 2H), 7.13 (s, $2H_{arom.}$), 7.30 (s, $2H_{arom.}$). ^{13}C NMR (125 MHz, MeOD-d_4) δ 16.6, 21.5, 55.1, 57.1, 111.9, 125.5, 128.5, 132.0, 133.9, 137.1, 155.0. HRMS, m/z calculated for $C_{11}H_{15}N_2O$ $[M+H]^+$: 191.1179. Found: 191.0981.

5-Chloro-2-(methoxymethyl)-1*H*-benzimidazole (5c) was obtained as purple solid in 86% yield. M.p. 127-129°C from methanol. ^{1}H NMR (500 MHz, MeOD-d_4) δ 3.67 (s, 3H, OMe), 5.06 (s, 2H), 7.52 (dd, $1H_{arom.}$, J = 1.8, 8.8 Hz), 7.72 (d, $1H_{arom.}$, J = 8.8 Hz), 7.77 (d, $1H_{arom.}$, J = 1.8 Hz). ^{13}C NMR (125 MHz, MeOD-d_4) δ 55.1, 57.0, 115.0, 116.3, 127.4, 131.9, 132.6, 134.0, 157.5. HRMS, m/z calculated for $C_9H_{10}ClN_2O$ $[M+H]^+$: 197.0476. Found: 197.0482.

2-(1-Methoxyethyl)-1*H*-benzimidazole (6) was obtained as beige solid in 89% yield. M.p. 109-111°C from methanol. ^{1}H NMR (500 MHz, MeOD-d_4) δ 1.71 (d, J = 6.8 Hz, 3H), 3.67 (s, 3H), 5.34 (q, J = 6.8 Hz, 1H), 7.57 (dd, $2H_{arom.}$, J = 3.1, 6.2 Hz), 7.77 (dd, $2H_{arom.}$, J = 3.1, 6.2 Hz). ^{13}C NMR (125 MHz, MeOD-d_4) δ 22.5, 55.1, 63.6, 115.0, 127.4, 132.3, 158.9. HRMS, m/z calculated for $C_{10}H_{13}N_2O$ $[M+H]^+$: 177.1022. Found: 177.1029.

Procedures for the synthesis of substituted (1-methylbenzimidazol-2-yl)methanol 7 and 8. Analogously as described in [2].

Potassium carbonate (5 mmol) was added to a stirring solution of benzimidazole-2-methanol (1 mmol) in DMF (2 mL). The reaction mixture was stirred at room temperature for 24 h prior to being diluted with methylene chloride (8 mL) and sodium chloride (8 mL). The organic layer was separated, and the aqueous layer was extracted with methylene chloride (8 mL × 2). The combined organic layers were dried over anhydrous sodium sulfate and concentrated in vacuo to provide a crude product, which was subjected to column purification using DCM/MeOH 100:5 (v/v) as eluent to yield the title compounds.

(1-Methylbenzimidazol-2-yl)methanol (7) [8] was obtained as beige solid in 30% yield. M.p. 134-136°C from methanol. ^{1}H NMR (500 MHz, MeOD-d_4) δ 3.89 (s, 3H, NMe), 4.85 (s, 2H), 7.25 (t, $1H_{arom.}$, J = 7.6 Hz), 7.31 (t, $1H_{arom.}$, J = 7.6 Hz), 7.49 (d, $1H_{arom.}$, J = 8.0 Hz), 7.61 (d, $1H_{arom.}$, J = 8.0 Hz). ^{13}C NMR (125 MHz, MeOD-d_4) δ 30.5, 57.7, 110.9, 119.7, 123.4, 124.2, 137.3, 142.5, 154.8. HRMS, m/z calculated for $C_9H_{11}N_2O$ $[M+H]^+$: 163.0866. Found: 163.0865.

2-(1-Hydroxyethyl)-1-methylbenzimidazole (8) [9] was obtained as beige solid in 87% yield. M.p. 197-199°C from methanol. ^{1}H NMR (500 MHz, MeOD-d_4) δ 1.73 (d, J = 6.9 Hz, 3H), 4.20 (s, 3H), 5.70 (q, J = 6.9 Hz, 1H), 7.71 (dd, $2H_{arom.}$, J = 3.1, 6.2 Hz), 7.94 (dd, $2H_{arom.}$, J = 3.1, 6.2 Hz). ^{13}C NMR (125 MHz, MeOD-d_4) δ 20.8, 33.0, 62.8, 113.9, 128.1, 133.5, 154.7. HRMS, m/z calculated for $C_{10}H_{13}N_2O$ $[M+H]^+$: 177.1022. Found: 177.1029.

General procedure for the reaction of benzimidazoles 1,2 with arenes in TfOH and H_2SO_4. Synthesis of compounds 9–11 (Table 3 and Scheme 2). Benzimidazole **1** or **2** (1 mmol) was added to the mixture of TfOH (1 mL) or H_2SO_4 (2 mL) and arene (2–18 mmol). Reaction mixture was stirred at room temperature for the time as indicated in Table 3 or Scheme 2. The mixture was poured into ice water (30 mL). After extraction with CH_2Cl_2 (3 × 30 mL), the combined extracts were consequently washed with water (50 mL), saturated aqueous solution of Na_2CO_3 (30 mL), water (50 mL), dried with anhydrous Na_2SO_4 and evaporated in vacuo to give crude products, which was subjected to chromatographic separation on silica gel using petroleum ether/diethyl ether as an eluent.

General procedure for the reaction of benzimidazole 1 with benzene under the action of strong Lewis acids $AlCl_3$ or $AlBr_3$. Synthesis of compound 9a (Table 3). Benzimidazole **1** (1 mmol) was added to the mixture of $AlCl_3$ or $AlBr_3$ (5.0 mmol) in benzene (3 mL). The reaction mixture was stirred at room temperature for the time as indicated in Table 1. The mixture was quenched with ice water (50 mL), extracted and worked-up as described above.

General procedures for the reaction of benzimidazoles 3–8 with arenes in TfOH in high pressure tube. Synthesis of compounds 12–14 (Tables 4, 5, Scheme 3). Solution of benzimidazole (0.1 mmol) in TfOH (1 mL) and arene (0.1 mL) was magnetically stirred at 140 °C in glass high pressure tube for 2.5 h, then poured into water (50 mL). After extraction with CH_2Cl_2 (3 × 30 mL), the combined extracts were consequently washed with water (50 mL), saturated aqueous solution of Na_2CO_3 (30 mL), water (50 mL), dried with anhydrous Na_2SO_4 and evaporated in vacuo to give crude products, which were subjected to chromatographic separation on silica gel using petroleum ether/diethyl ether as an eluent.

2-Diphenylmethyl-1-methyl-1*H*-benzimidazole (9a). White solid. M.p. 135-137°C. ^{1}H NMR (500 MHz, $CDCl_3$) δ 3.61 (s, 3H, Me), 5.80 (s, 1H, CH), 7.25 (s, $3H_{arom}$), 7.27–7.35 (m, $10H_{arom}$), 7.81 (d, $1H_{arom}$, *J*=7.9). ^{13}C NMR (125 MHz, $CDCl_3$) δ 30.1, 50.1, 108.9, 120.1, 121.8, 122.4, 127.1, 128.7, 129.1, 135.9, 140.0, 142.4, 155.2. HRMS, m/z calculated for $C_{21}H_{19}N_2$ $[M+H]^+$: 299.1543. Found: 299.1548.

1-Methyl-2-[bis(4-methylphenyl)methyl]-1*H*-benzimidazole (9b) and **1-methyl-2-[α-(4-methylphenyl)-α-(2-methylphenyl)methyl]-1*H*-benzimidazole (9c)** were obtained as an oily mixture with the ratio of 6:1. Compound **9b**: ^{1}H NMR (500 MHz, $CDCl_3$, from the spectrum of the mixture) δ 2.38 (s, 6H, 2Me), 3.61 (s, 3H, NMe), 5.72 (s, 1H, CH), 7.18–7.32 (m, $11H_{arom.}$), 7.87–7.89 (m, $1H_{arom.}$). Compound **9c**: ^{1}H NMR (500 MHz, $CDCl_3$, from the spectrum of the mixture) δ 2.33 (s, 3H, Me), 2.40 (s, 3H, Me), 3.58 (s, 3H, NMe), 7.18–7.32 (m, $11H_{arom.}$), 7.87–7.89 (m, $1H_{arom.}$). For the mixture of compounds **9b,c**: ^{13}C NMR (125 MHz, $CDCl_3$) δ 20.9, 29.9, 46.6, 49.3,

108.8, 119.9, 121.5, 122.1, 128.72, 128.74, 129.1, 129.24, 129.28, 135.8, 136.5, 137.1, 155.5. HRMS, m/z calculated for $C_{23}H_{23}N_2$ $[M+H]^+$: 327.1856. Found: 327.1860.

1-Methyl-2-[bis(4-methoxyphenyl)methyl]-1*H*-benzimidazole (9d) and **1-methyl-2-[α-(4-methoxyphenyl)-α-(2-methoxyphenyl)methyl]-1*H*-benzimidazole (9e)** were obtained as an oily mixture with the ratio of 5:1. Compound **9d**: ^{1}H NMR (500 MHz, $CDCl_3$, from the spectrum of the mixture) δ 3.60 (s, 3H, NMe), 3.78 (s, 6H, 2MeO), 5.66 (s, 1H, CH), 6.85–7.29 (m, $11H_{arom.}$), 7.80–7.81 (m, $1H_{arom.}$). Compound **9e**: ^{1}H NMR (500 MHz, $CDCl_3$, from the spectrum of the mixture) δ 3.63 (s, 3H, NMe), 3.75 (s, 3H, MeO), 3.78 (s, 3H, MeO), 6.12 (s, 1H, CH), 6.85–7.29 (m. $11H_{arom.}$), 7.80–7.81 (m, $1H_{arom.}$). For the mixture of compounds **9d,e**: ^{13}C NMR (125 MHz, $CDCl_3$) δ 30.1, 41.7, 48.5, 55.2, 55.7, 108.9, 110.5, 114.0, 114.07, 114.09, 120.0, 121.0, 121.8, 122.4, 128.3, 130.0, 130.3, 134.4, 135.9, 155.8, 158.6. HRMS, m/z calculated for $C_{23}H_{23}N_2O_2$ $[M+H]^+$: 359.1754. Found: 359.1751.

1-Methyl-2-[bis(4-chlorophenyl)methyl]-1*H*-benzimidazole (9f) and **1-methyl-2-[α-(4-chlorophenyl)-α-(2-chlorophenyl)methyl]-1*H*-benzimidazole (9g)** were obtained as an oily mixture with the ratio 3:1. Compound **9f**: ^{1}H NMR (500 MHz, $CDCl_3$, from the spectrum of the mixture) δ 3.62 (s, 3H, NMe), 5.67 (s, 1H, CH), 7.17-7.33 (m, $11H_{arom.}$), 7.80–7.82 (m, $1H_{arom.}$). Compound **9g**: ^{1}H NMR (500 MHz, $CDCl_3$, from the spectrum of the mixture) δ 3.66 (s, 3H, NMe), 6.16 (s, 1H, CH), 7.17-7.33 (m, $11H_{arom.}$), 7.80–7.82 (m, $1H_{arom.}$). For the mixture of compounds **9f,g**: ^{13}C NMR (125 MHz, $CDCl_3$) δ 30.1 , 45.7, 48.6, 109.1, 120.0, 122.2, 122.8, 127.4, 128.9, 129.0, 129.6, 130.3, 130.6, 130.7, 133.3, 138.0, 154.1. HRMS, m/z calculated for $C_{21}H_{17}Cl_2N_2$ $[M+H]^+$: 367.0763. Found: 367.0766.

1-Methyl-2-[bis(3,4-dimethylphenyl)methyl]-1*H*-benzimidazole (9h). Oily compound. ^{1}H NMR (500 MHz, $CDCl_3$) δ 2.21 (s, 6H, 2Me), 2.23 (s, 6H, 2Me), 3.60 (s, 3H, Me), 5.64 (s, 1H, CH), 6.97 (dd, $2H_{arom.}$, J = 7.8 Hz), 7,03 (s, $2H_{arom.}$), 7.08 (d, $2H_{arom.}$, J = 7.8 Hz), 7.22-7.26 (m, $1H_{arom.}$), 7.26-7.31 (m, $2H_{arom.}$), 7.82 (d, 1H, J = 7.3 Hz). ^{13}C NMR (125 MHz, $CDCl_3$) δ 19.4, 19.8, 30.1, 49.4, 108.9, 120.1, 121.7, 122.2, 126.4, 129.86, 129.88, 130.2, 135.3, 135.9, 136.7, 137.5, 155.8. HRMS, m/z calculated for $C_{25}H_{27}N_2$ $[M+H]^+$: 355.2169. Found: 355.2174.

1-Methyl-2-[bis(2,4-dimethylphenyl)methyl]-1*H*-benzimidazole (9i). White solid. M.p. 224-226°C. ^{1}H NMR (500 MHz, $CDCl_3$) δ 2.16 (s, 6H, 2Me), 2.30 (s, 6H, 2Me), 3.52 (s, 3H, Me), 5.77 (s, 1H, CH), 6.71 (d, $2H_{arom.}$, J = 7.8 Hz), 6.91 (d, $2H_{arom.}$, J = 7.1 Hz), 7.02 (s, $2H_{arom.}$), 7.20-7.24 (m, $2H_{arom.}$), 7.27-7.30 (m, $1H_{arom.}$), 7.78 (d, $1H_{arom.}$, J = 7.8 Hz). ^{13}C NMR (125 MHz, $CDCl_3$) δ 19.4, 21.0, 29.7, 43.3, 108.8, 120.2, 121.6, 122.1, 127.1, 128.9, 131.3, 134.9, 135.7, 135.9, 136.6, 142.6, 155.6. HRMS, m/z calculated for $C_{25}H_{27}N_2$ $[M+H]^+$: 355.2169. Found: 355. 2173.

1-Methyl-2-[bis(2,5-dimethylphenyl)methyl]-1*H*-benzimidazole (9j). White solid. M.p. 205-207°C. ^{1}H NMR (500 MHz, $CDCl_3$) δ 2.13 (s, 6H, 2Me), 2.19 (s, 6H, 2Me), 3.55 (s, 3H, NMe),

5.79 (s, 1H, CH), 6.66 (s, $2H_{arom.}$); 7.0 (d, $2H_{arom.}$, J = 7.6 Hz), 7.09 (d, $2H_{arom.}$, J = 7.6 Hz), 7.21-7.24 (m, $1H_{arom.}$), 7.27-7.33 (m, $2H_{arom.}$), 7.78 (d, $1H_{arom.}$, J = 7.6 Hz). ^{13}C NMR (125 MHz, $CDCl_3$) δ 19.0, 21.1, 29.6, 43.8, 108.7, 120.2, 121.4, 122.0, 127.8, 129.5, 130.2, 132.8, 135.7, 135.8, 137.4, 142.7, 155.4. HRMS, m/z calculated for $C_{25}H_{27}N_2$ $[M+H]^+$: 355.2169. Found: 355. 2168.

1-Methyl-2-[bis(3,4-dichlorophenyl)methyl]-1*H*-benzimidazole (9k). White solid. M.p. 147-149°C. ^{1}H NMR (500 MHz, $CDCl_3$) δ 3.65 (s, 3H, Me), 5.62 (s, 1H, CH), 7.09 (dd, $2H_{arom.}$, J = 8.4 Hz), 7.28-7.32 (m, $2H_{arom.}$), 7.33-7.34 (m, $3H_{arom.}$), 7.41 (s, $1H_{arom.}$), 7.42 (s, $1H_{arom.}$), 7.80 (d, $1H_{arom.}$, J = 7.8 Hz). ^{13}C NMR (125 MHz, $CDCl_3$) δ 30.1, 48.0, 109.2, 120.2, 122.4, 123.1, 128.3, 130.8, 130.9, 132.0, 133.1, 135.7, 139.2, 152.8. HRMS, m/z calculated for $C_{21}H_{15}Cl_4N_2$ $[M+H]^+$: 434.9984. Found: 434.9991.

1-Methyl-2-[bis(2,4,5-trimethylphenyl)methyl]-1*H*-benzimidazole (9l). White solid. M.p. 239-241°C. ^{1}H NMR (500 MHz, $CDCl_3$) δ 2.08 (s, 6H, 2Me), 2.10 (s, 6H, 2Me), 2.20 (s, 6H, 2Me), 3.53 (s, 3H, NMe), 5.72 (s, 1H, CH), 6.60 (s, $2H_{arom.}$), 6.96 (s, $2H_{arom.}$), 7.20-7.24 (m, $1H_{arom.}$), 7.27-7.31 (m, $2H_{arom.}$), 7.78 (d, 1H, J = 7.6 Hz). ^{13}C NMR (125 MHz, $CDCl_3$) δ 18.9, 19.3, 19.5, 29.7, 43.3, 108.7, 120.3, 121.4, 122.0, 130.2, 131.9, 133.0, 134.2, 135.0, 135.1, 136.0, 156.0. HRMS, m/z calculated for $C_{27}H_{31}N_2$ $[M+H]^+$: 383.2482. Found: 383.2479.

2-(1,1-Diphenylethyl)-1*H*-benzimidazole (10). White solid. M.p. 228-230°C. ^{1}H NMR (500 MHz, $CDCl_3$) δ 2.30 (s, 3H, Me), 7.12-7.28 (m, $12H_{arom.}$), 7.53 (br.s, $2H_{arom.}$). ^{13}C NMR (125 MHz, $CDCl_3$) δ 28.8, 50.9, 115.1, 122.9, 127.2, 128.0, 128.6, 145.7, 158.6. HRMS, m/z calculated for $C_{21}H_{19}N_2$ $[M+H]^+$: 299.1543. Found: 299.1549.

2-(1-Phenylethenyl)-1*H*-benzimidazole (11). White solid. M.p. 221-223°C. ^{1}H NMR (500 MHz, $CDCl_3$) δ 5.70 (s, 1H, $=CH_2$), 6.40 (s, 1H, $=CH_2$), 7.26 (dd, $2H_{arom.}$, J = 6.0, 3.1 Hz), 7.28-7.29 (m, $3H_{arom.}$), 7.36-7.37 (m, $2H_{arom.}$), 7.58 (dd, $2H_{arom.}$, J = 6.0, 3.1 Hz). HRMS, m/z calculated for $C_{15}H_{13}N_2$ $[M+H]^+$: 221.1073. Found: 221.1076.

2-Benzyl-1*H*-benzimidazole (12a) [10]. M.p. 184-186°C. ^{1}H NMR (500 MHz, $(CD_3)_2CO$) δ 4.25 (s, 2H), 7.14 (td, $2H_{arom.}$, J = 9.0, 3.0 Hz), 7.22 (t, $1H_{arom.}$, J = 7.5 Hz), 7.29 (t, $2H_{arom.}$, J = 7.5 Hz), 7.35 (d, $2H_{arom.}$, J = 7.5 Hz), 7.48 (td, $2H_{arom.}$, J = 9.0, 3.0 Hz). ^{13}C NMR (125 MHz, $(CD_3)_2CO$) δ 36.3, 115.7, 122.5, 127.6, 129.5, 129.9, 138.7, 154.5. HRMS, m/z calculated for $C_{14}H_{13}N_2$ $[M+H]^+$: 209.1073. Found: 209.1066.

2-(3,4-Dichlorobenzyl)-1*H*-benzimidazole (**12b**) and **2-(2,3-dichlorobenzyl)-1*H*-benzimidazole** (**12c**). Compound **12b** and **12c** were obtained as a mixture with M.p. 165-167°C (for the ratio of 1:0.5). Compound **12b**: ^{1}H NMR (500 MHz, $(CD_3)_2CO$, from the spectrum of the mixture) δ 4.28 (s, 2H), 7.14–7.16 (m, $2H_{arom.}$), 7.36–7.33 (m, $1H_{arom.}$), 7.47–7.51 (m, $3H_{arom.}$), 7.60 (m, $1H_{arom.}$). Compound **12c**: ^{1}H NMR (500 MHz, $(CD_3)_2CO$, from the spectrum of the mixture) δ 4.45 (s, 2H), 7.14-7.16 (m, $2H_{arom.}$), 7.31 (t, $1H_{arom.}$, J = 8.0 Hz), 7.39 (d, $1H_{arom.}$, J = 7.5 Hz), 7.47-

7.51 (m, 3H). For the mixture of compounds **12b,c**: ^{13}C NMR (125 MHz, $(CD_3)_2CO$) δ 34.8, 35.1, 122.5, 122.6, 128.8, 129.3,130.1, 130.2, 130.8, 130.9, 131.4, 131.9, 132.6, 138.9, 139.6, 152.5, 153.2. HRMS (for mixture of isomers), m/z calculated for $C_{14}H_{11}Cl_2N_2$ $[M+H]^+$: 277.0294. Found: 277.0298.

2-(2,4-Dibromobenzyl)-1*H*-benzimidazole (12d) and **2-(3,5-dibromobenzyl)-1*H*-benzimidazole (12e)**. Compound **12d** and **12e** were obtained as a mixture with M.p. 204-206°C (for the ratio of 1:0.25). Compound **12d**: ^{1}H NMR (500 MHz, $(CD_3)_2CO$, from the spectrum of the mixture) δ 4.37 (s, 2H), 7.17–7.12 (m, $2H_{arom.}$), 7.37 (d, $1H_{arom.}$, J = 8.5 Hz,), 7.48–7.49 (m, $2H_{arom.}$), 7.54 (dd, $1H_{arom.}$, J = 8.0, 2.0 Hz), 7.81 (d, $1H_{arom.}$, J = 2.0 Hz). Compound **12e**: ^{1}H NMR (500 MHz, $(CD_3)_2CO$, from the spectrum of the mixture) δ 4.69 (s, 2H), 7.11–7.13 (m, $2H_{arom.}$), 7.19 (t, $1H_{arom.}$, J = 8.0Hz), 7.44–7.46 (m, $2H_{arom.}$),7.69 (d, $2H_{arom.}$, J = 8.0Hz). HRMS (for mixture of isomers), m/z calculated for $C_{14}H_{11}Br_2N_2$ $[M+H]^+$: 364.9283. Found: 364.9278.

2-Benzyl-5,7-dimethylbenzimidazole (12f). M.p. 186-188°C.^{1}H NMR (500 MHz, $(CD_3)_2CO$) δ 2.35 (s, 3H, Me), 2.46 (s, 3H, Me), 4.19 (s, 2H), 6.78 (s, $1H_{arom.}$), 7.08 (s, $1H_{arom.}$), 7.20 (t, $1H_{arom.}$, J = 7.0 Hz), 7.27 (t, $2H_{arom.}$, J = 7.5 Hz), 7.32 (d, $2H_{arom.}$, J = 7.0 Hz). ^{13}C NMR (125 MHz, $(CD_3)_2CO$) δ 17.1, 21.8, 36.4, 124.5, 127.5, 129.5, 129.8, 131.9, 139.1.

2-Benzyl-5-chloro-1*H*-benzimidazole (12g) [11]. M.p. 153-155°C.^{1}H NMR (500 MHz, MeOD-d_4) δ 4.19 (s, 2H), 7.15 (dd, $1H_{arom.}$, J = 8.6, 1.8 Hz), 7.19–7.24 (m, $1H_{arom.}$), 7.27–7.29 (m, $4H_{arom.}$), 7.42 (d, $1H_{arom.}$, J = 8.6 Hz), 7.47 (d, $1H_{arom.}$, J = 1.8 Hz). ^{13}C NMR (125 MHz, MeOD-d_4) δ 36.1, 115.5, 116.4, 123.7, 128.1, 128.9, 129.76, 129.8, 137.9, 156.7.

2-(1-Phenylethyl)-1*H*-benzimidazole (12h) [12]. M.p. 206-208°C.^{1}H NMR (500 MHz, $(CD_3)_2CO$) δ 1.77 (d, 3H, J = 7.5 Hz), 4.43 (q, 1H, J = 7.0 Hz), 7.11-7.14 (m, $2H_{arom.}$),7.21 (t, $1H_{arom.}$, J = 7.5 Hz), 7.30 (t, $2H_{arom.}$, J = 7.5 Hz), 7.38-7.33 (m, $2H_{arom.}$), 7.47-7.49 (m, $2H_{arom.}$). ^{13}C NMR (125 MHz, $(CD_3)_2CO$) δ 21.2, 41.1, 122.5, 127.7, 128.5, 129.6.

2-Benzyl-1-methyl-1*H*-benzimidazole (13). Oily compound. ^{1}H NMR (500 MHz, $CDCl_3$) δ 3.55 (s, 3H, NMe), 4.30 (s, 2H, CH_2), 7.19-7.28 (m, $8H_{arom.}$), 7.74-7.76 (m, $1H_{arom.}$). ^{13}C NMR (125 MHz, $CDCl_3$) δ 30.1, 34.3, 109.1, 119.3, 122.1, 122.4, 127.0, 128.5, 128.9, 136.0, 142.2, 153.2. HRMS, m/z calculated for $C_{15}H_{15}N_2$ [M+H]: 223.1230. Found: 223.1237.

1-Methyl-2-(1-phenylethyl)-1*H*-benzimidazole (14). Oily compound. ^{1}H NMR (500 MHz, $CDCl_3$) δ 2.00 (d, 3H, J = 7.2 Hz), 3.94 (s, 3H, NMe), 5.20 (q, 1H, J = 7.2 Hz), 7.21 (d, $2H_{arom.}$, J = 7.6 Hz), 7.35 (t, $1H_{arom.}$, J = 7.6 Hz), 7.41 (t, $2H_{arom.}$, J = 7.6 Hz), 7.62 (dd, $2H_{arom.}$, J = 6.2, 3.0 Hz), 7.71 (dd, $2H_{arom.}$, J = 6.2, 3.0 Hz). ^{13}C NMR (125 MHz, $CDCl_3$) δ 15.9, 32.7, 35.0, 112.7, 126.8, 127.3, 128.6, 129.8, 131.8, 135.9, 154.3. HRMS, m/z calculated for $C_{16}H_{17}N_2$ [M+H]: 237.1386. Found: 237.1395.

2. References

1. M. J. Frisch, G. W. Trucks, H. B. Schlegel, G. E. Scuseria, M. A. Robb, J. R. Cheeseman, G. Scalmani, V. Barone, B. Mennucci, G. A. Petersson, H. Nakatsuji, M. Caricato, X. Li, H. P. Hratchian, A. F. Izmaylov, J. Bloino, G. Zheng, J. L. Sonnenberg, M. Hada, M. Ehara, K. Toyota, R. Fukuda, J. Hasegawa, M. Ishida, T. Nakajima, Y. Honda, O. Kitao, H. Nakai, T. Vreven, J. A. Montgomery Jr., J. E. Peralta, F. Ogliaro, M. Bearpark, J. J. Heyd, E. Brothers, K. N. Kudin, V. N. Staroverov, R.Kobayashi, J. Normand, K. Raghavachari, A. Rendell, J. C. Burant, S. S. Iyengar, J. Tomasi, M. Cossi, N. Rega, J. M. Millam, M. Klene, J. E. Knox, J. B. Cross, V. Bakken, C. Adamo, J. Jaramillo, R. Gomperts, R. E. Stratmann, O. Yazyev, A. J. Austin, R. Cammi, C. Pomelli, J. W. Ochterski, R. L. Martin, K. Morokuma, V. G. Zakrzewski, G. A. Voth, P. Salvador, J. J. Dannenberg, S. Dapprich, A. D. Daniels, Ö. Farkas, J. B. Foresman, J. V. Ortiz, J. Cioslowski, D. J. Fox,Gaussian 09, Revision C.01, Gaussian, Inc., Wallingford CT, **2010**.

2. R. Wang, C. Chen, X. Zhang, C. Zhang, Q. Zhong, G. Chen, Q. Zhang, S. Zheng, G. Wang, Q.-H. Chen. *J. Med. Chem*. **2015**, *58*, 4713–4726.

3. M. J. Plater, P. Barnes, L. K. McDonald, S. Wallace, N. Archer, T. Gelbrich, P. N. Horton, M. B. Hursthouse. *Org. Biomol. Chem*. **2009**, *7*, 1633-1641.

4. V.M. Reddy, K.R. Reddy. *Chemical & Pharmaceutical Bulletin*. **2010**, *58*, 953-956.

5. X. Jing, Q. Zhu, F. Xu, X. Ren, D. Li, C. Yan. *Synthetic Commun.* **2006**, 36, 2597-2601.

6. C. T. Bahner, H. A. Rutter, L. M. Rives. *J. Am. Chem. Soc.* **1952**, *74*, 3689-3690.

7. G. K. Hughes, F. Lions, E. Ritchie. *Journal and Proceedings of the Royal Society of New South Wales*. **1938**, *72*, 209-222.

8. P. K. Dubey, P. V. V. Prasada, R. K. Srinivas. *Synthetic Commun.* **2007**, *37*, 1675-1681.

9. G. Muller, J. P. Riehl, K. J. Schenk, G. Hopfgartner, C. Piguet, J.-C. G. Bünzli. *Eur. J. Inorg. Chem*. **2002**, 3101-3110.

10. A. J. Blacker, M. M. Farah, M. I. Hall, S. P. Marsden, O. Saidi, J. M. J. Williams. *Org. Lett.* **2009**, *11*, 2039-2042.

11. X. Diao, Y. Wang, Y. Jiang, D. Ma. *J. Org. Chem.* **2009**, *74*, 7974-7977.

12. J. Charton, S. Girault-Mizzi, C. Sergheraert. *Chem. Pharm. Bull.* **2005**, *53*, 492-497.

3. ¹H and ¹³C NMR Spectra of compounds 1–14

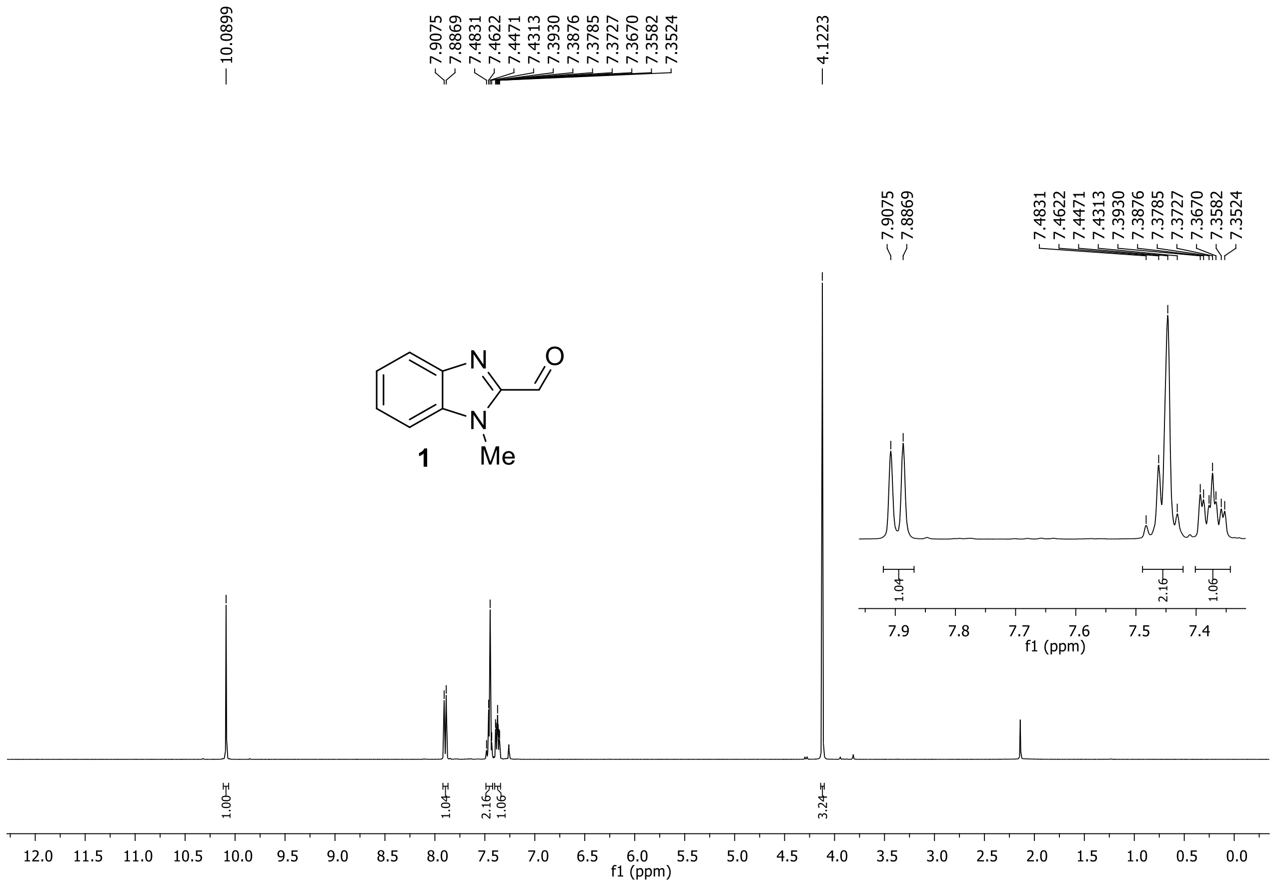

Figure S1. ¹H NMR spectrum of compound **1** (500 MHz, $CDCl_3$).

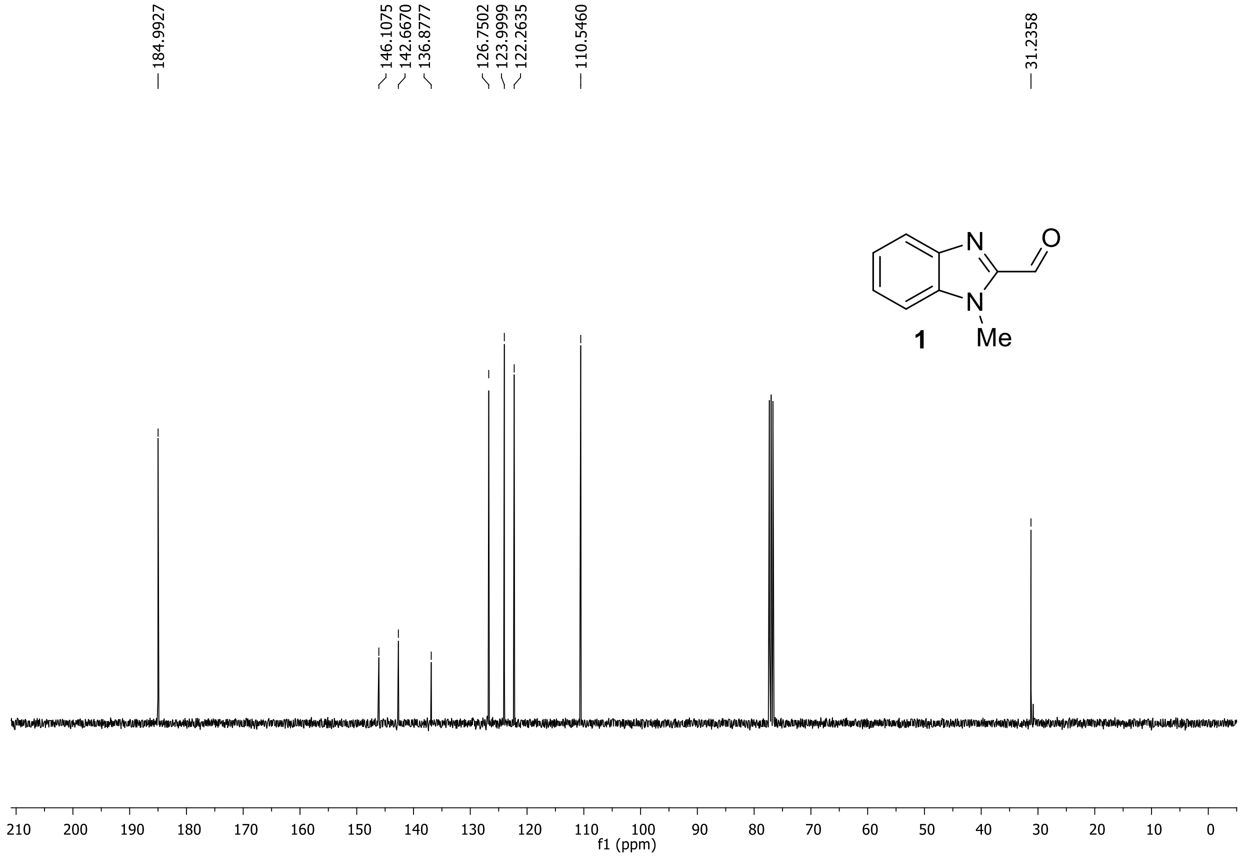

Figure S2. ¹³C NMR spectrum of compound **1** (125 MHz, $CDCl_3$).

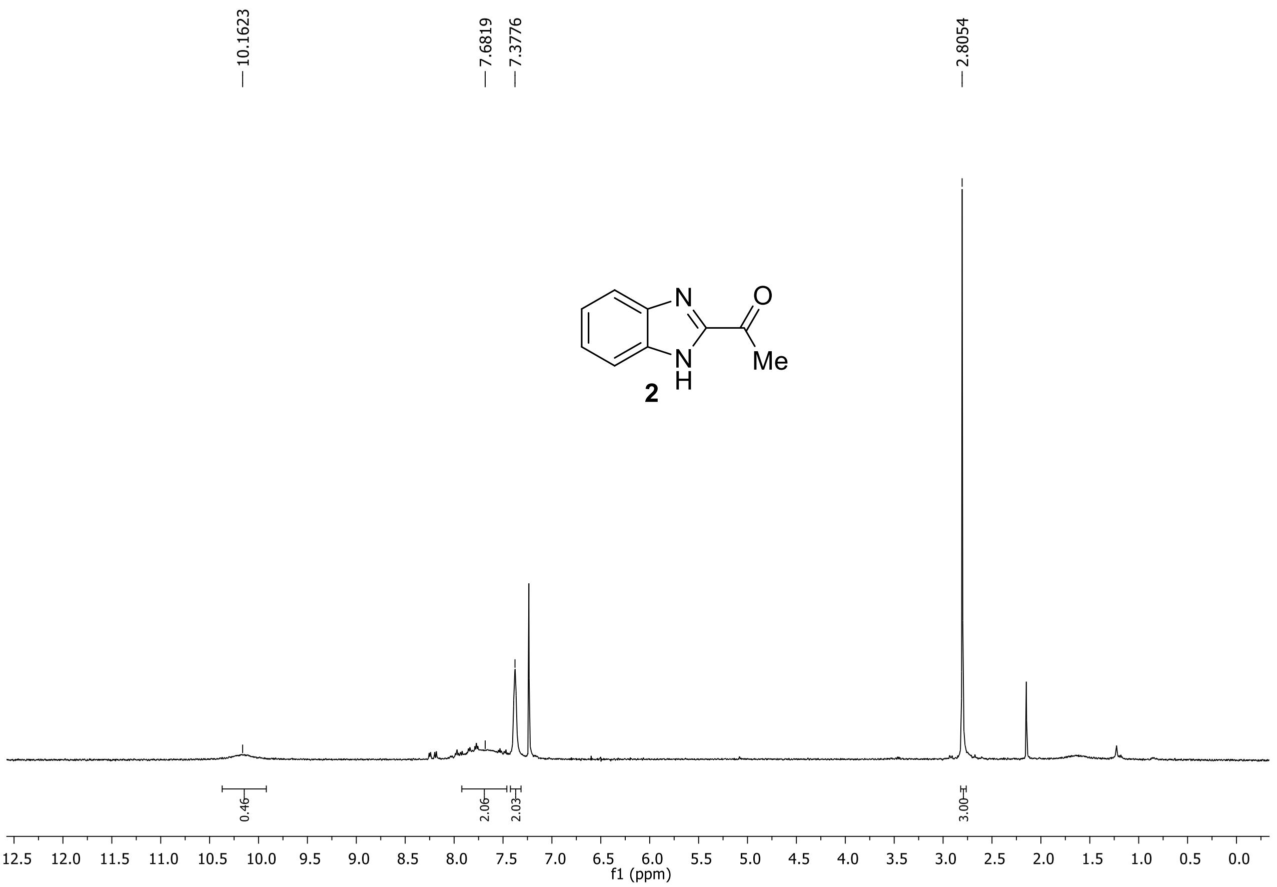

Figure S3. ^{1}H NMR spectrum of compound **2** (500 MHz, $CDCl_3$).

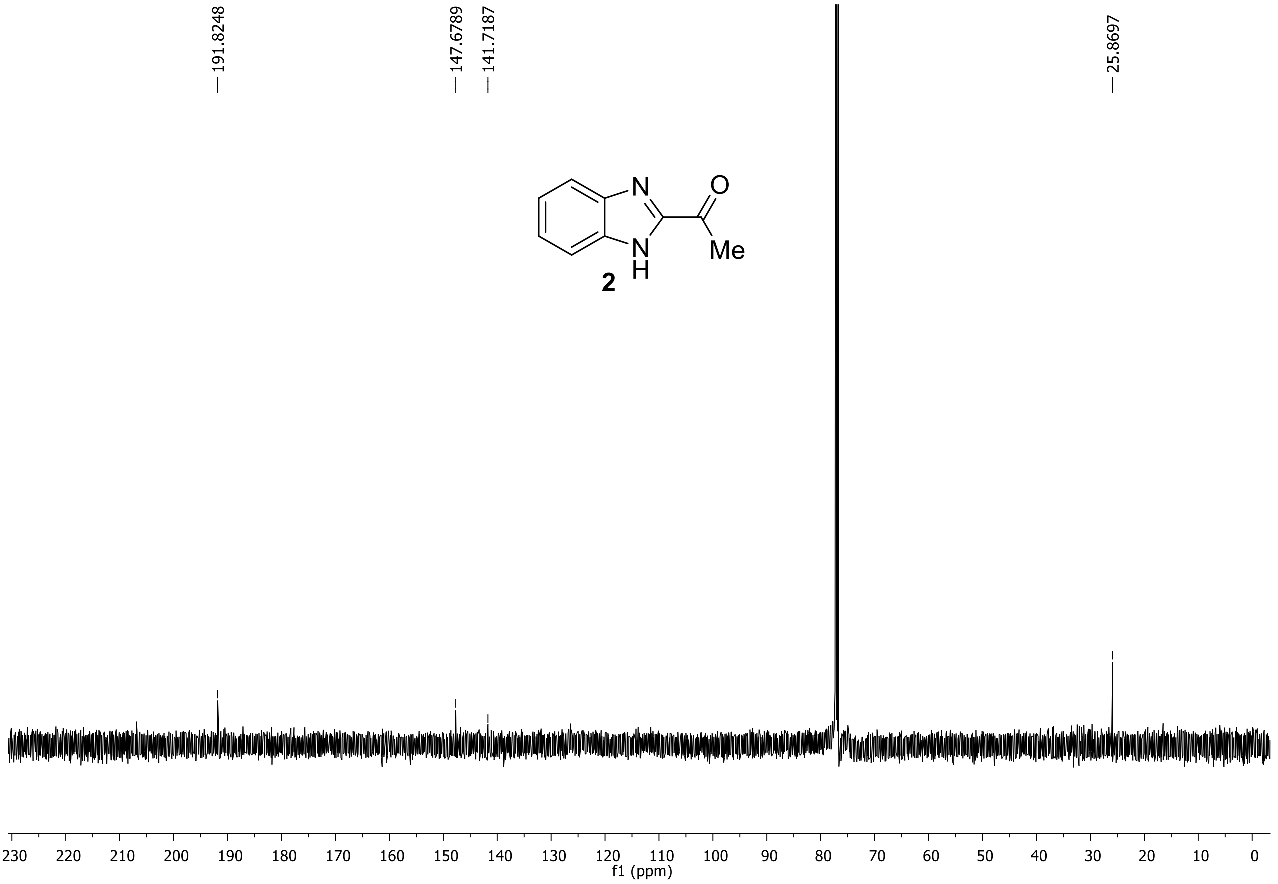

Figure S4. ^{13}C NMR spectrum of compound **2** (125 MHz, $CDCl_3$).

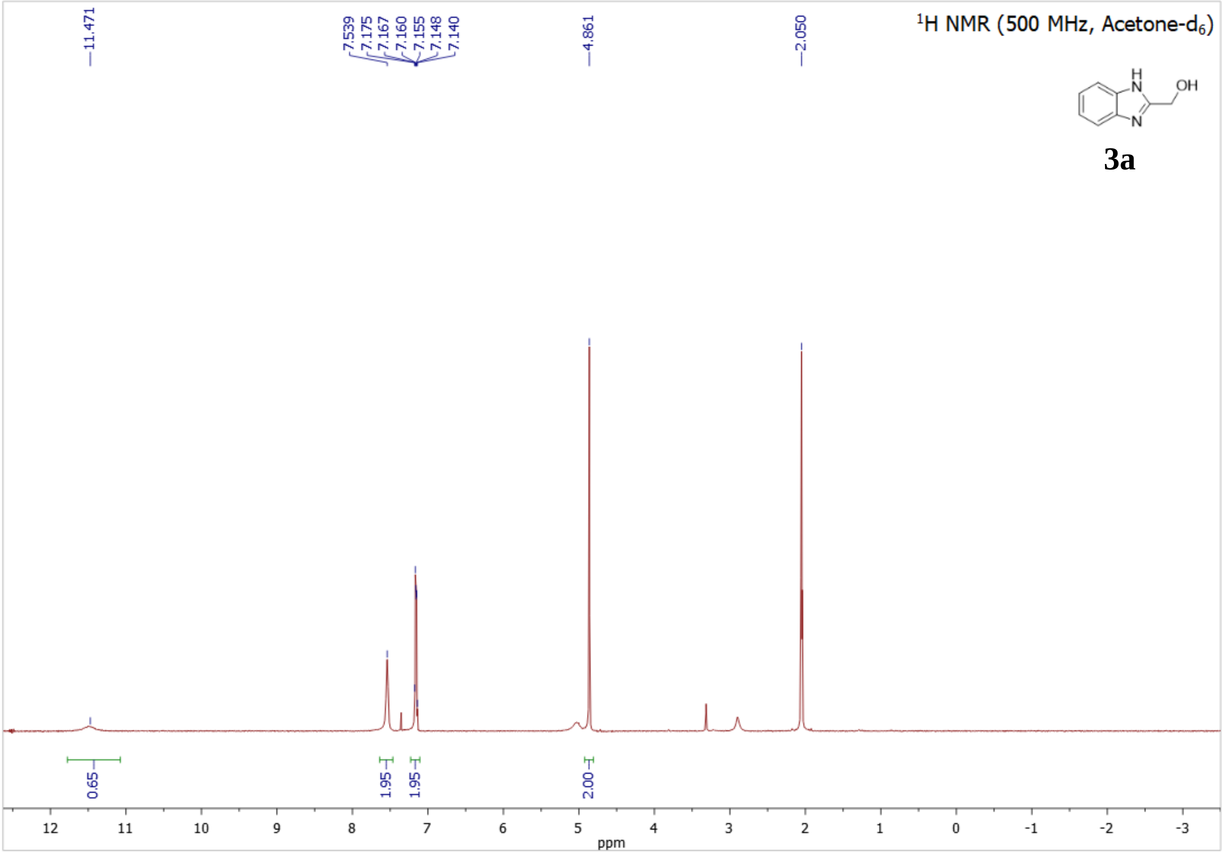

Figure S5. ^{1}H NMR spectrum of compound **3a** (500 MHz, $(CD_3)_2CO$).

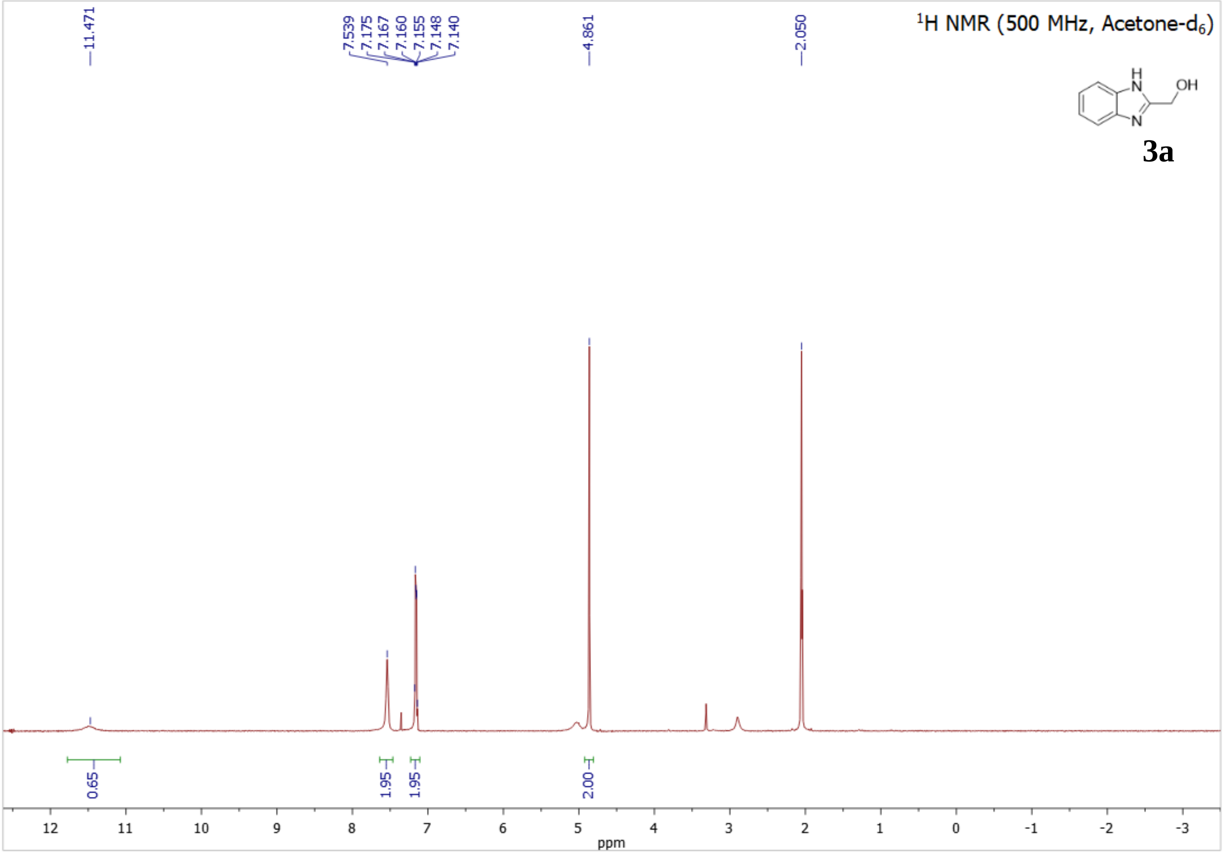

Figure S6. ^{13}C NMR spectrum of compound **3a** (125 MHz, $(CD_3)_2CO$).

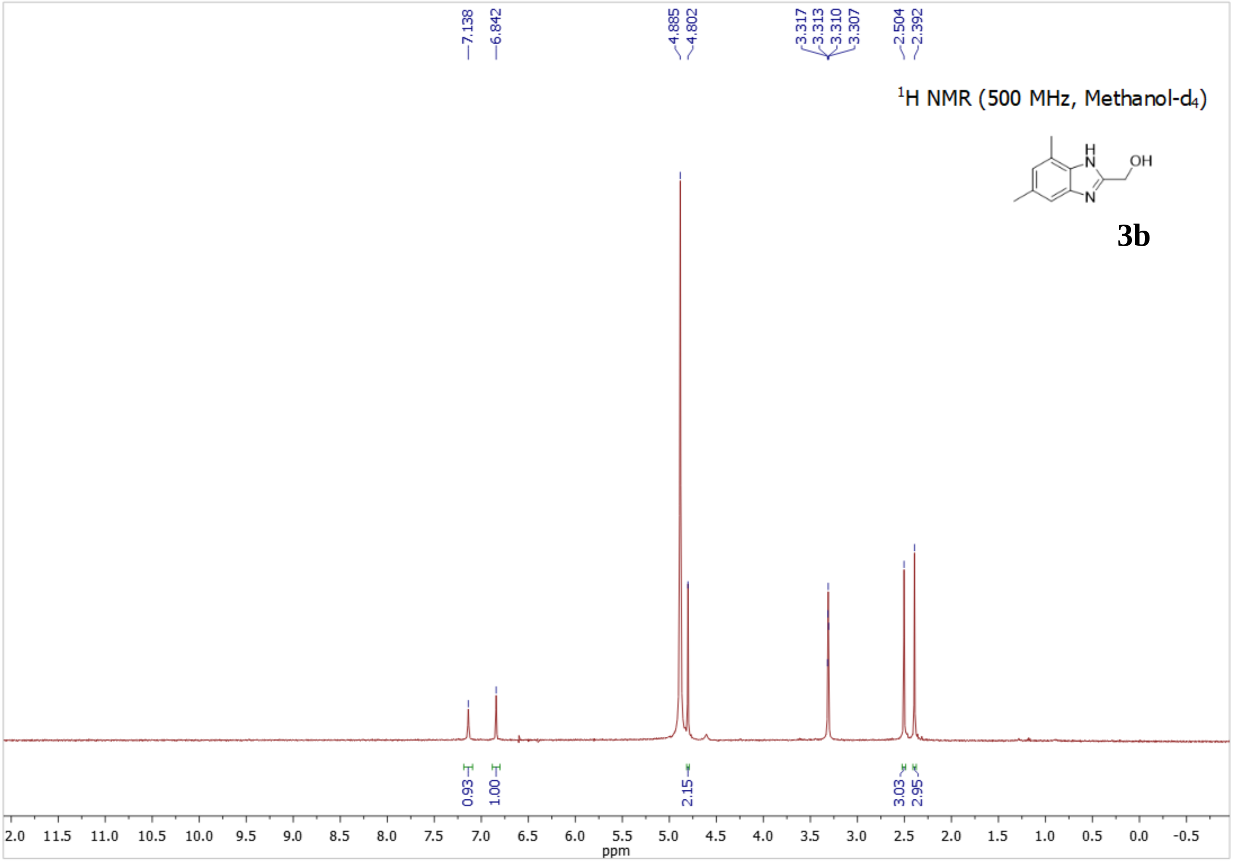

Figure S7. ^{1}H NMR spectrum of compound **3b** (500 MHz, MeOD-d_4).

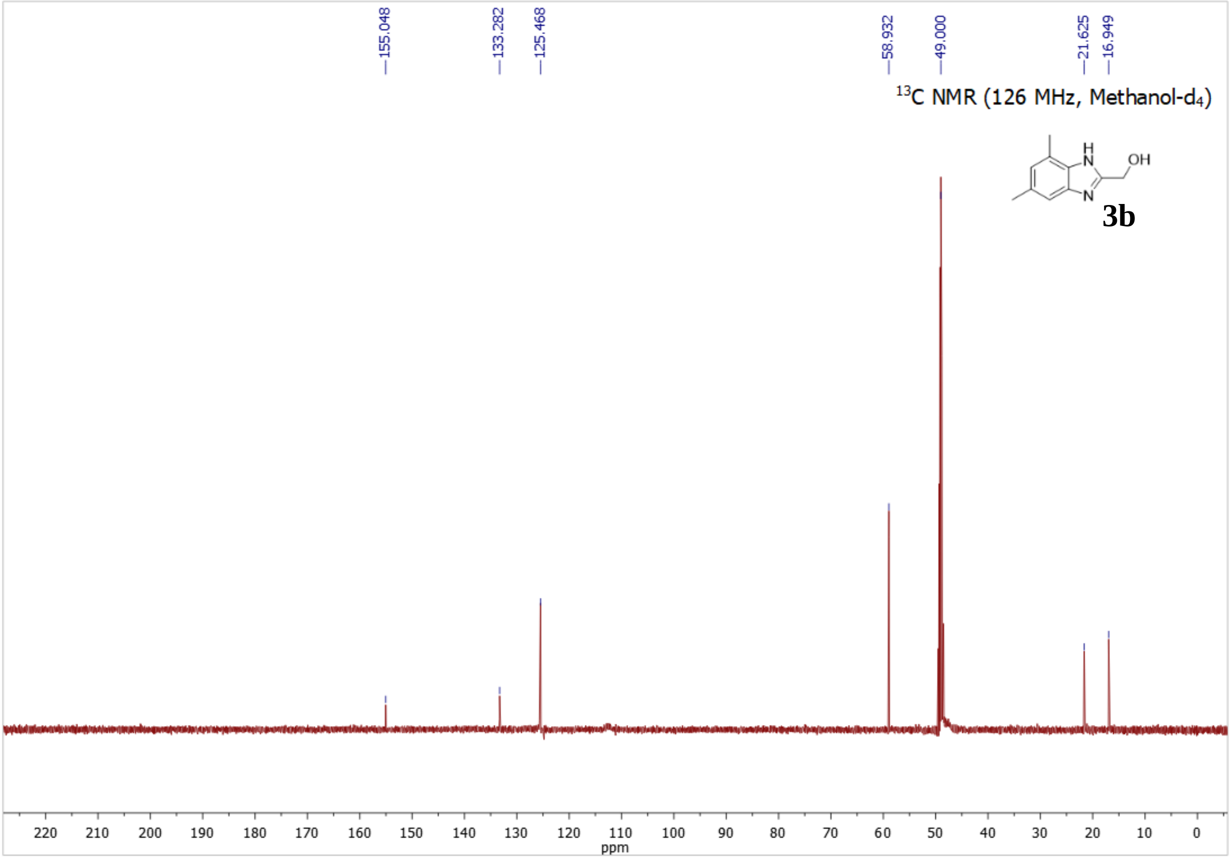

Figure S8. ^{13}C NMR spectrum of compound **3b** (125 MHz, MeOD-d_4).

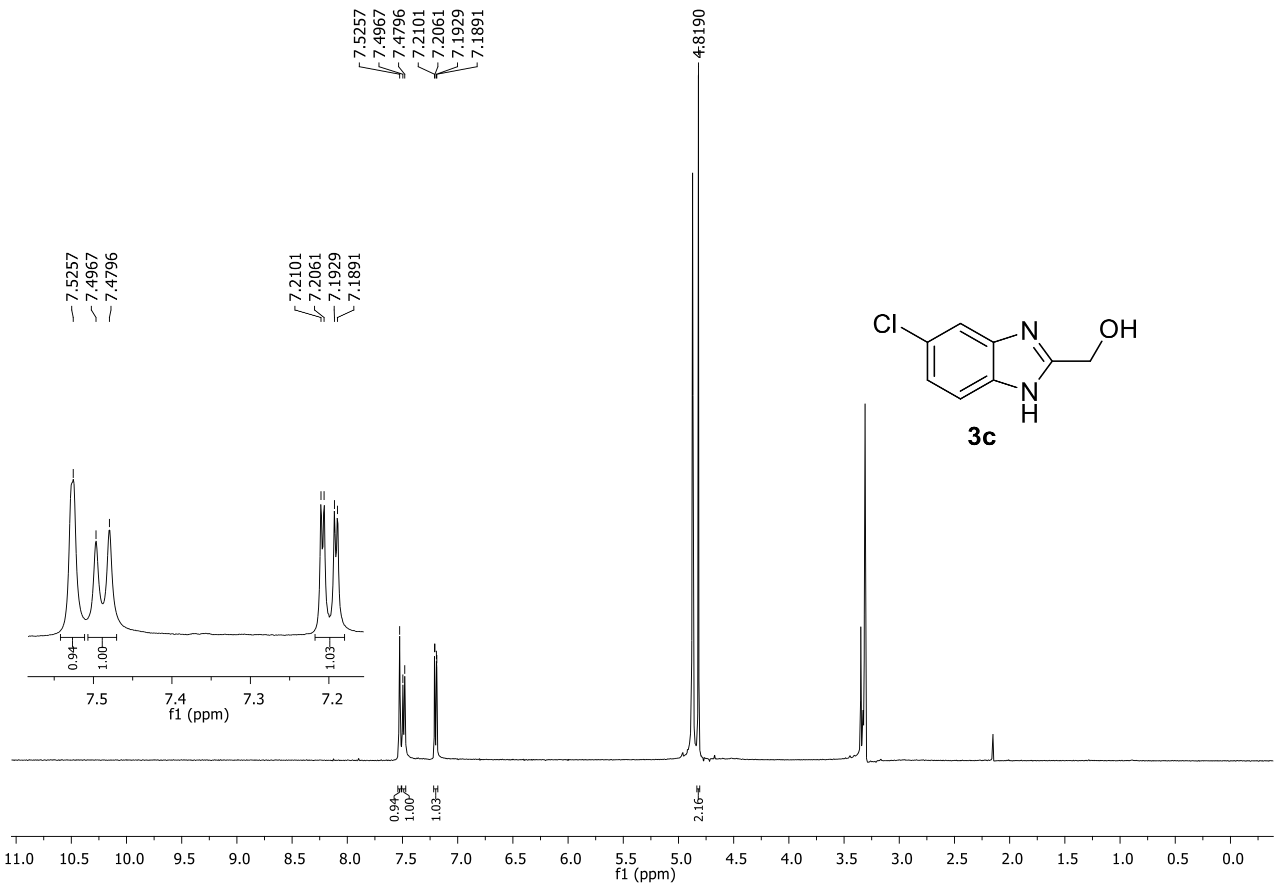

Figure S9. ^{1}H NMR spectrum of compound **3c** (500 MHz, MeOD-d_4).

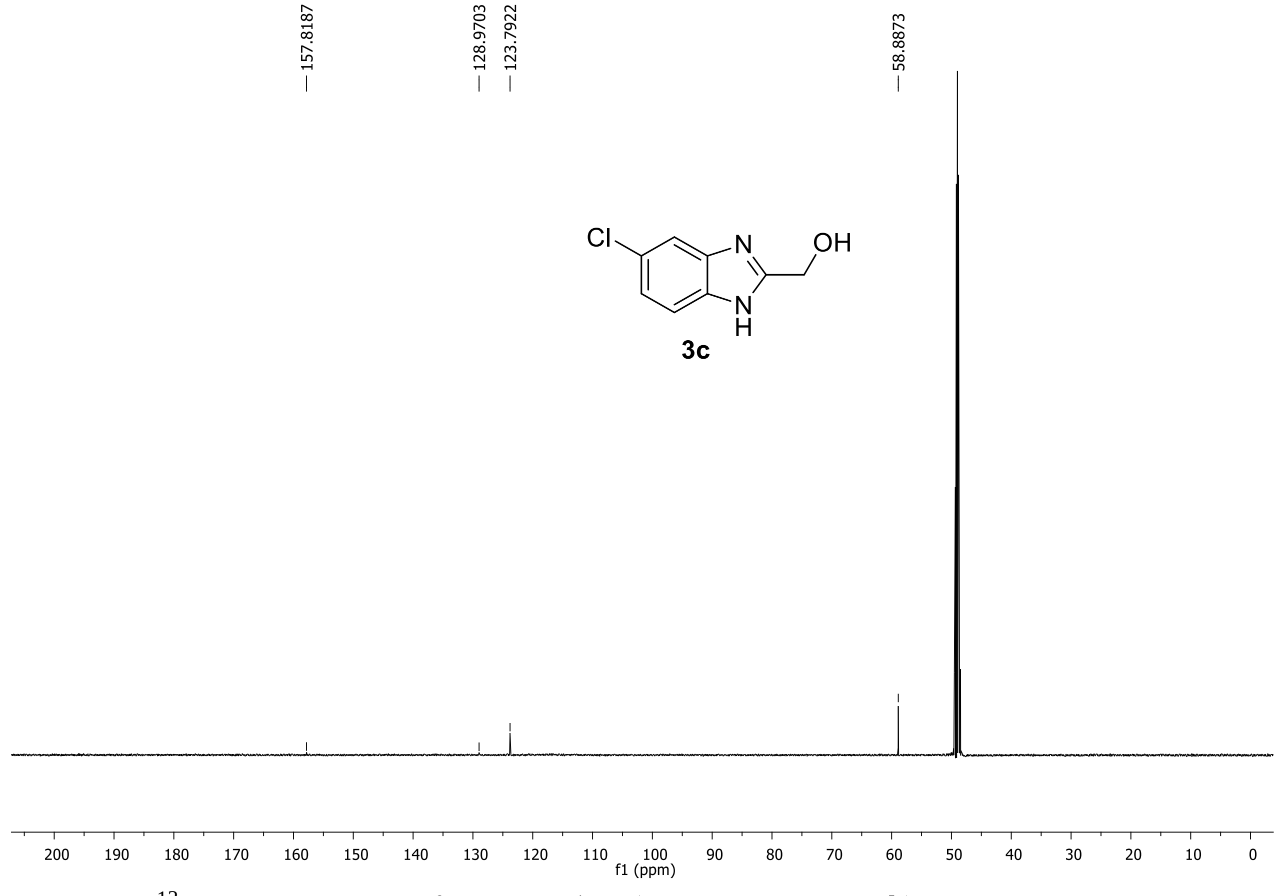

Figure S10. ^{13}C NMR spectrum of compound **3c** (125 MHz, MeOD-d_4).

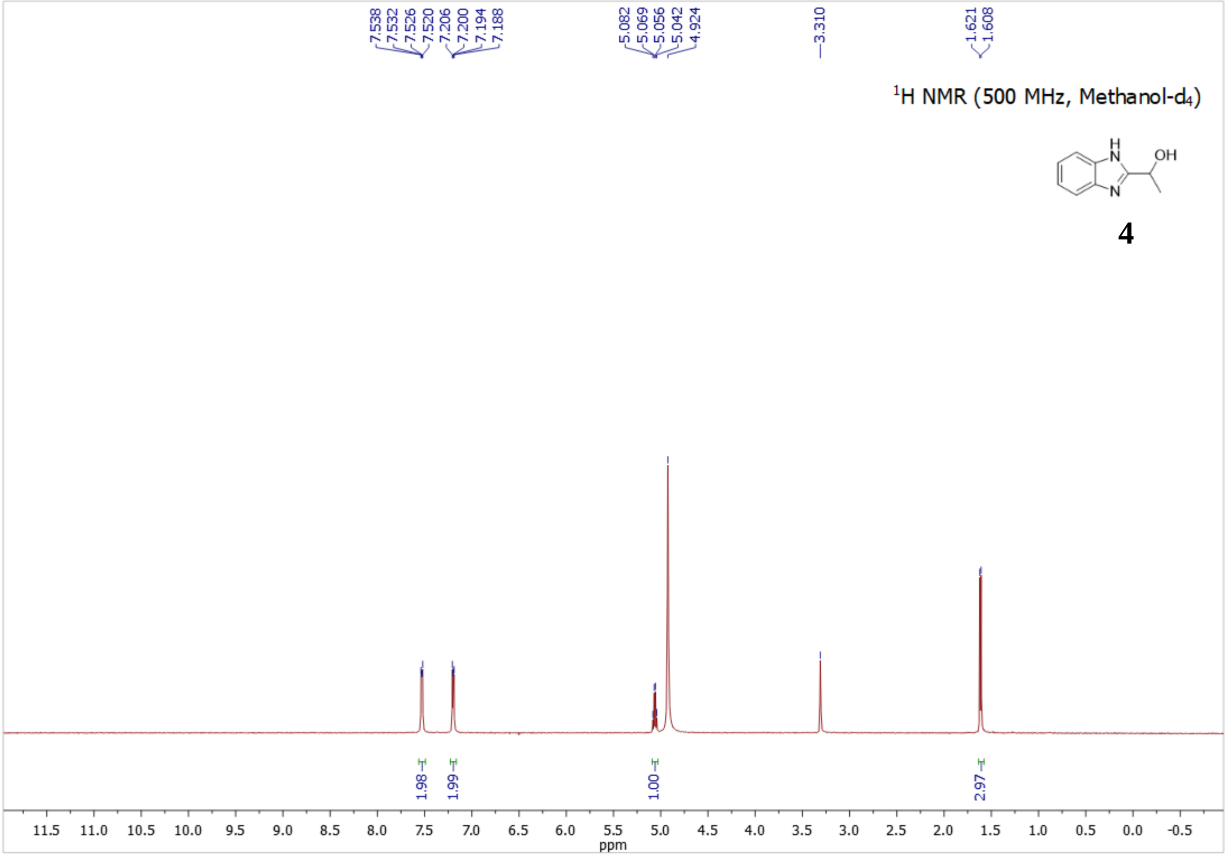

Figure S11. ^{1}H NMR spectrum of compound **4** (500 MHz, MeOD-d_4).

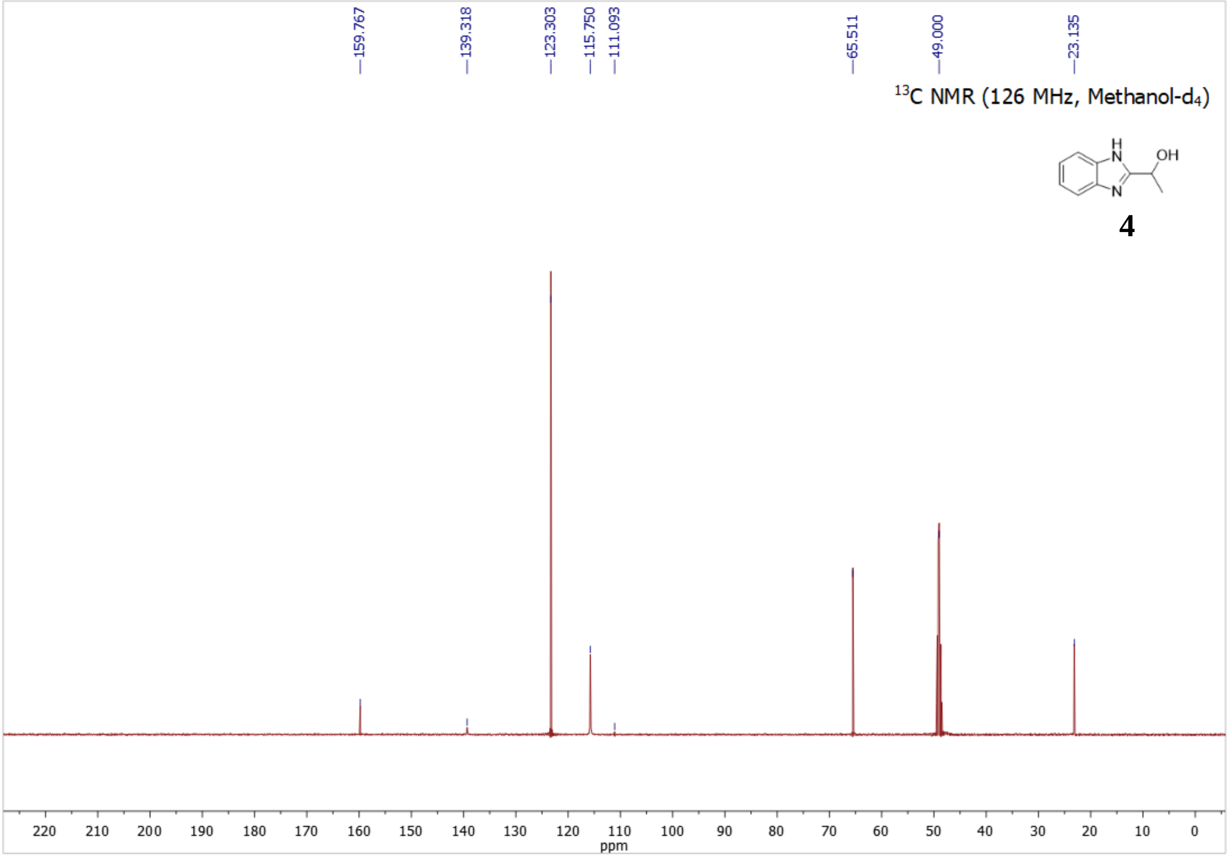

Figure S12. ^{13}C NMR spectrum of compound **4** (125 MHz, MeOD-d_4).

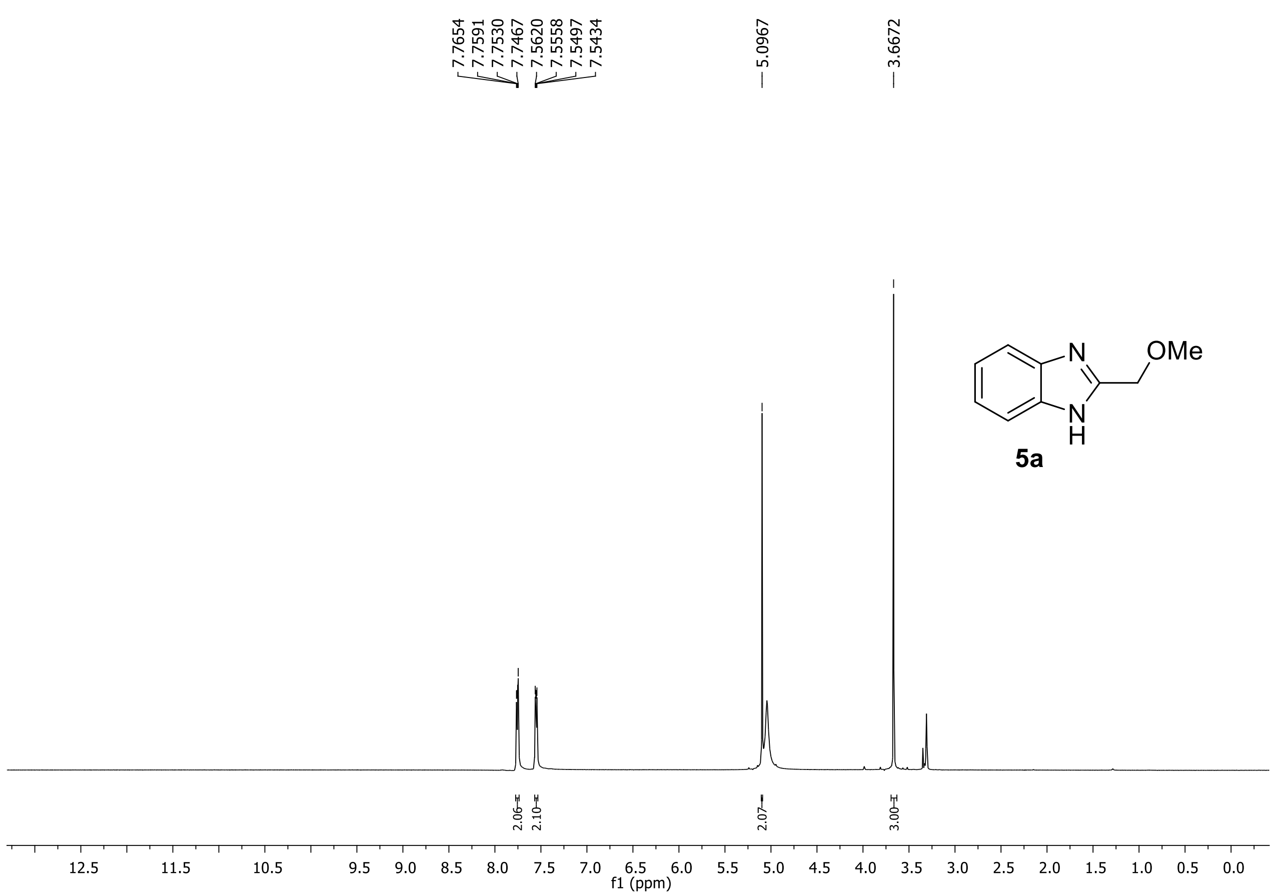

Figure S13. ^{1}H NMR spectrum of compound **5a** (500 MHz, MeOD-d_4).

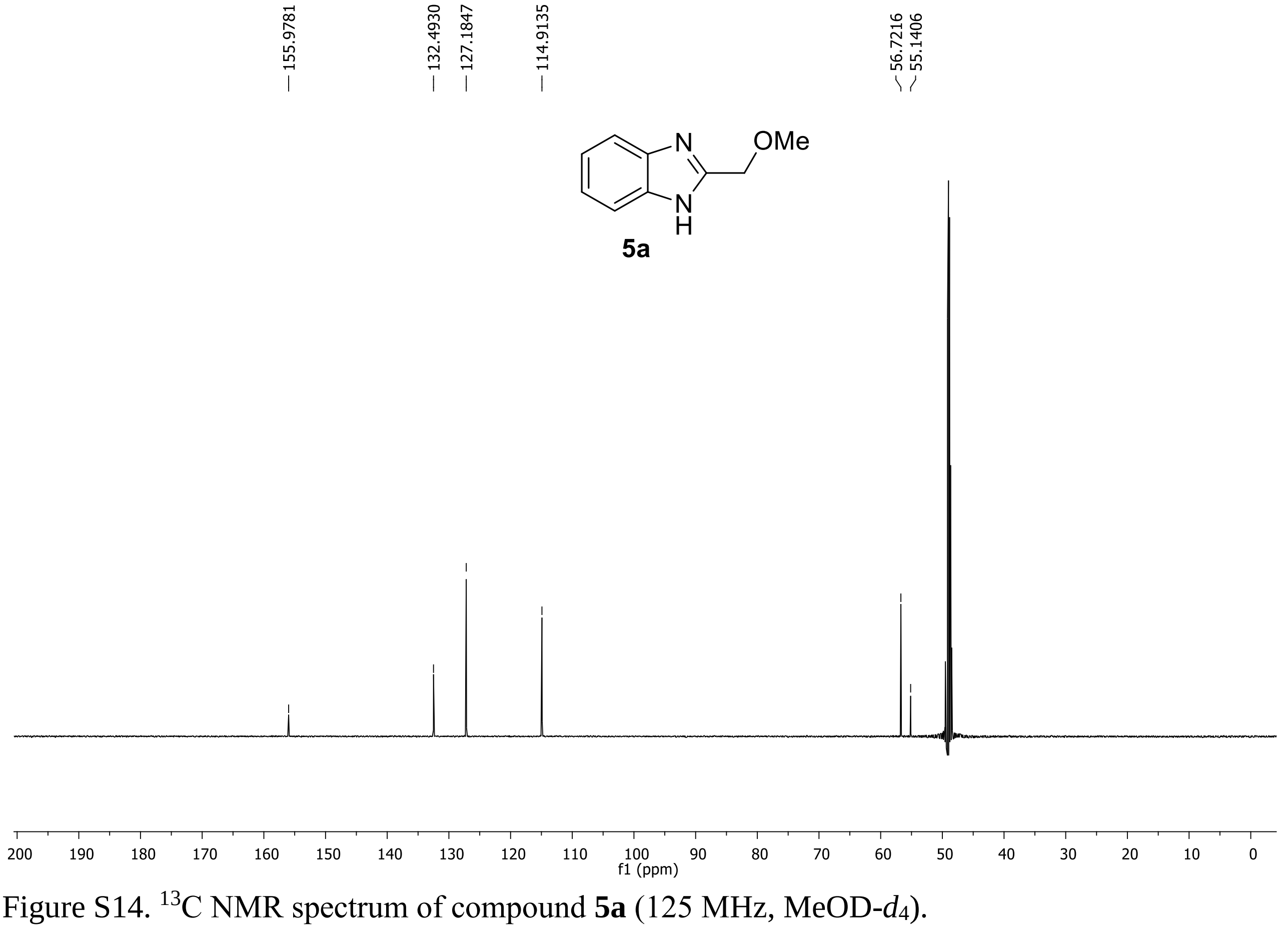

Figure S14. ^{13}C NMR spectrum of compound **5a** (125 MHz, MeOD-d_4).

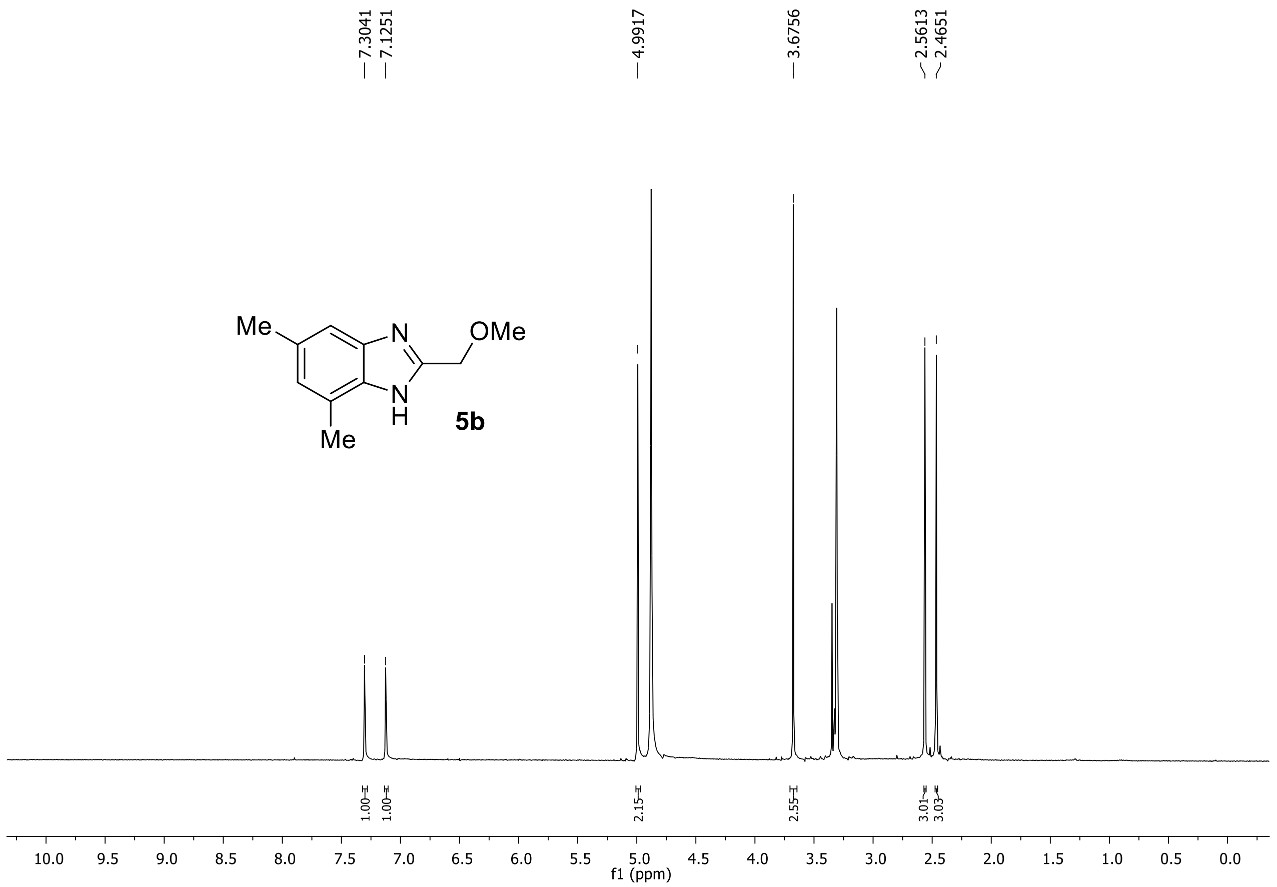

Figure S15. ^{1}H NMR spectrum of compound **5b** (500 MHz, MeOD-d_4).

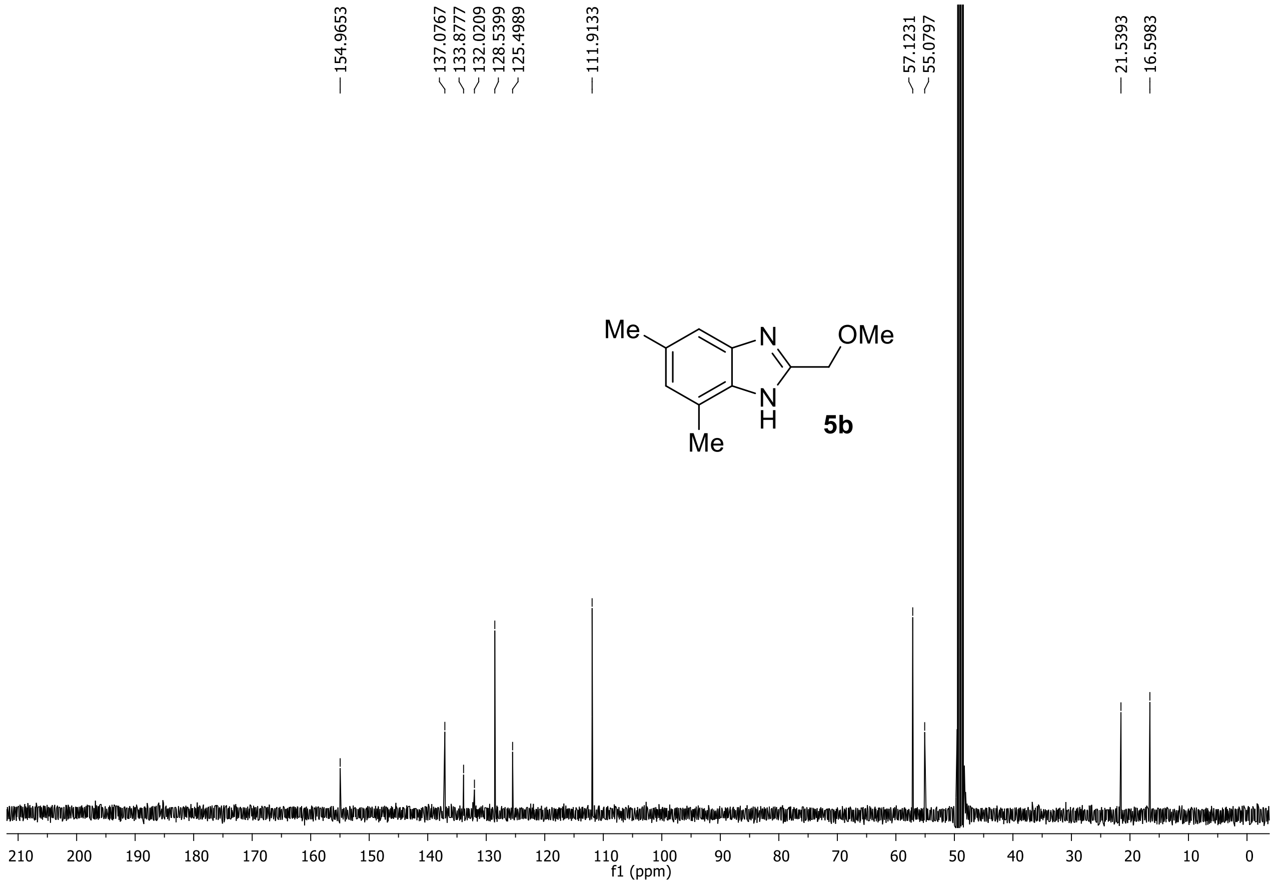

Figure S16. ^{13}C NMR spectrum of compound **5b** (125 MHz, MeOD-d_4).

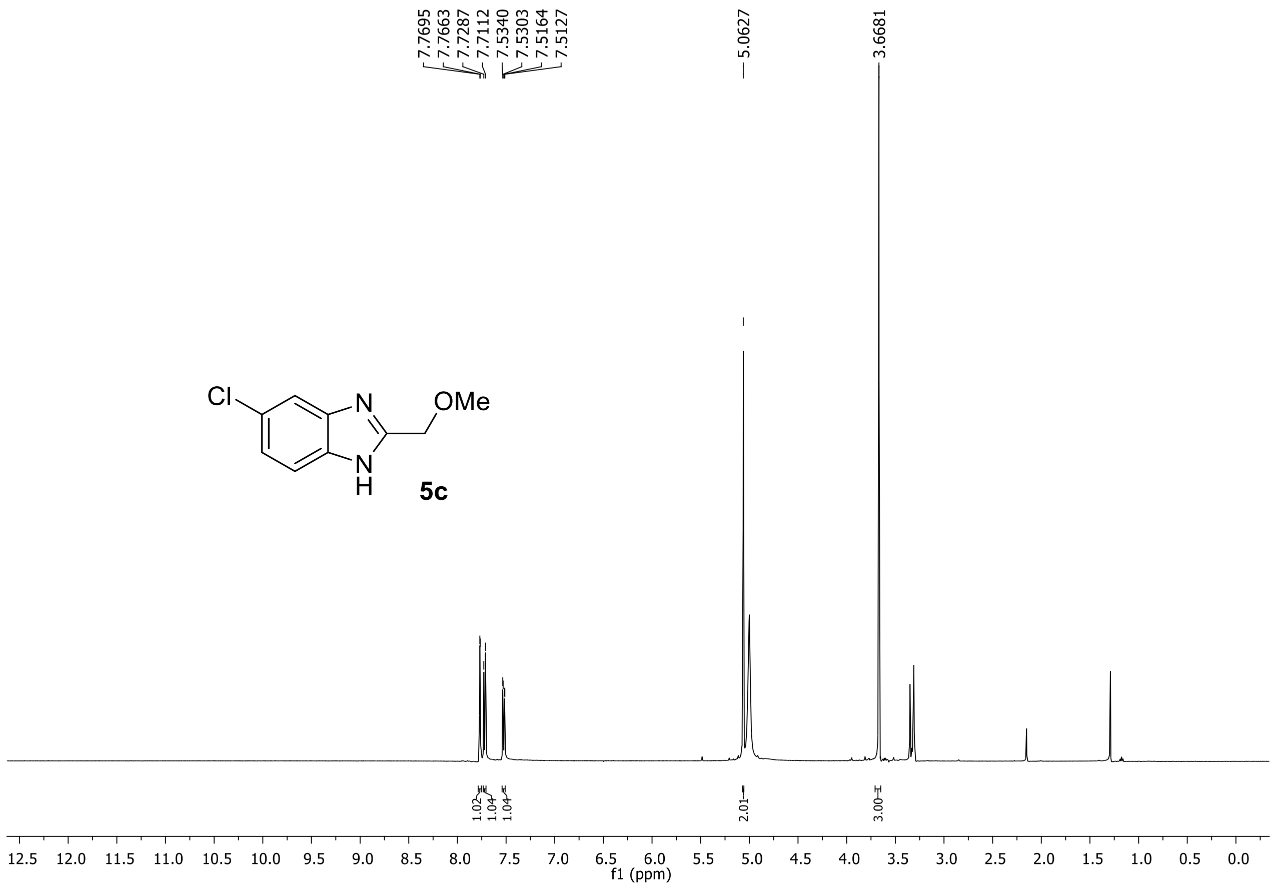

Figure S17. ¹H NMR spectrum of compound **5c** (500 MHz, MeOD-d_4).

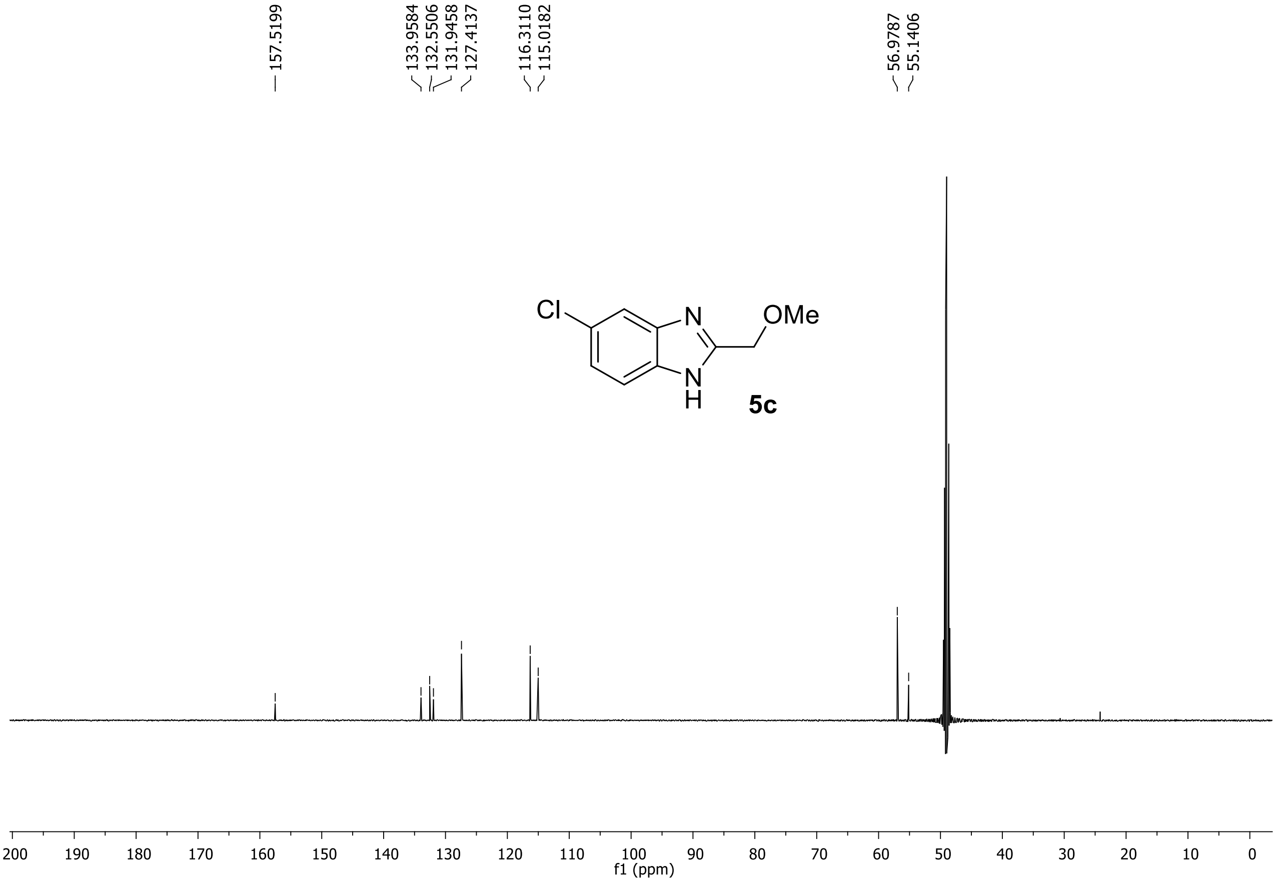

Figure S18. ¹³C NMR spectrum of compound **5c** (125 MHz, MeOD-d_4).

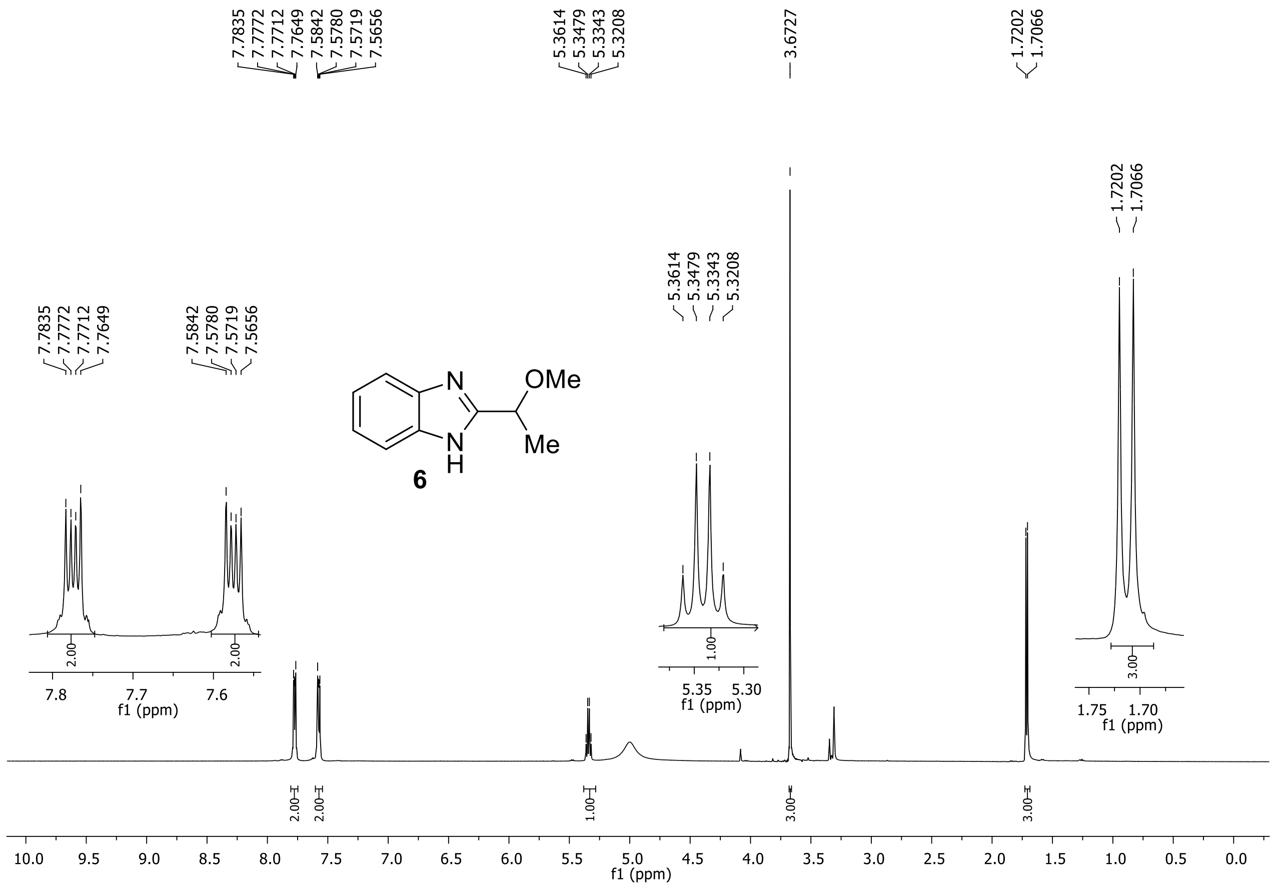

Figure S19. ^{1}H NMR spectrum of compound **6** (500 MHz, MeOD-d_4).

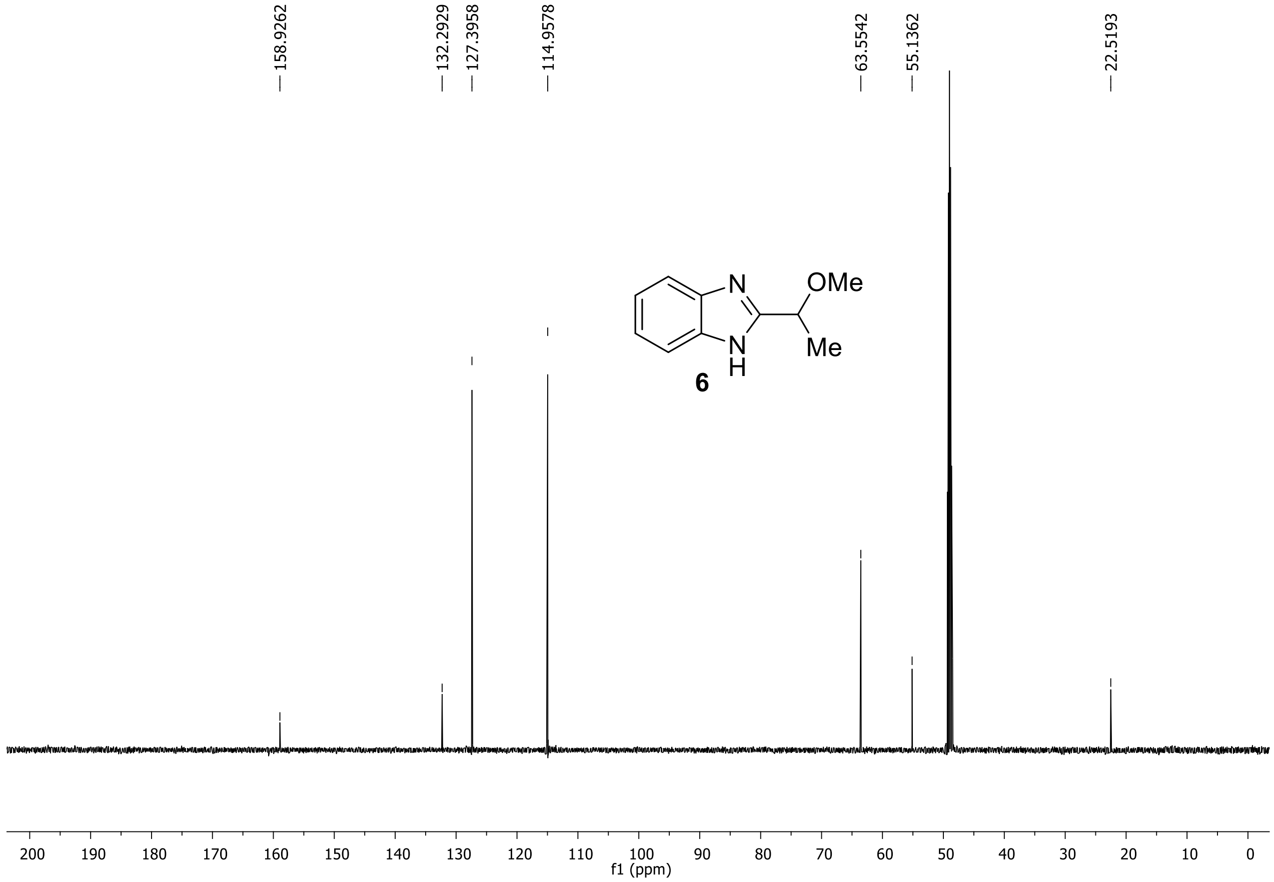

Figure S20. ^{13}C NMR spectrum of compound **6** (125 MHz, MeOD-d_4).

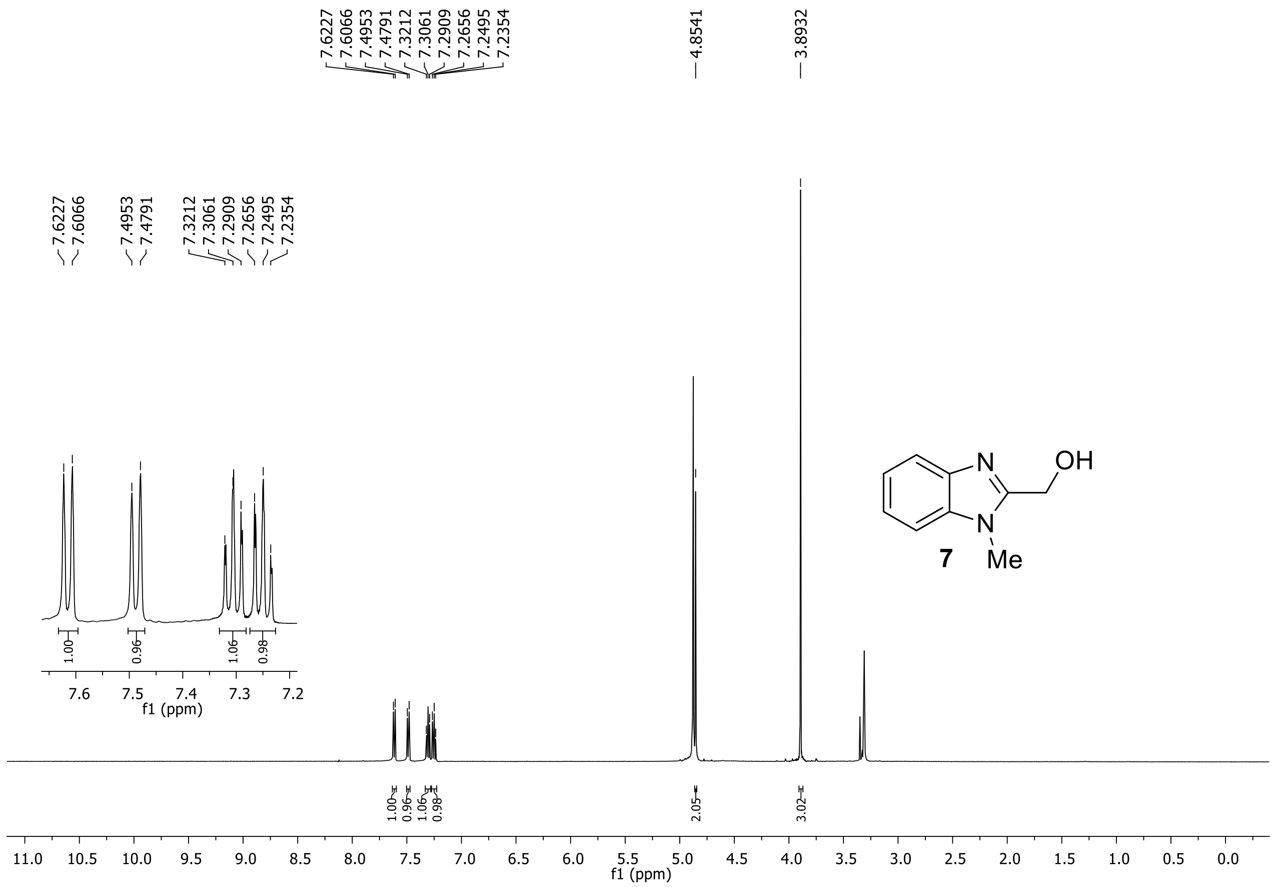

Figure S21. ^{1}H NMR spectrum of compound **7** (500 MHz, MeOD-d_4).

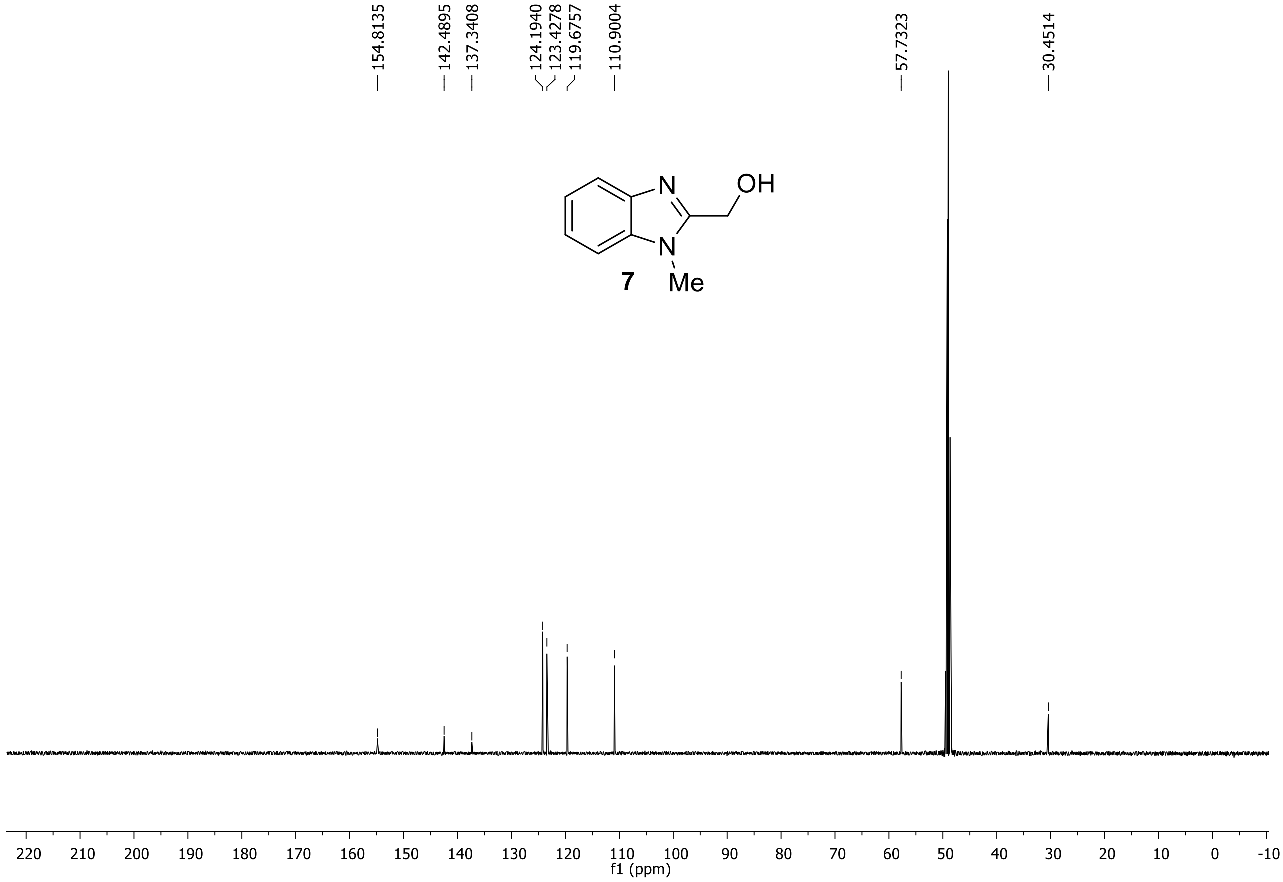

Figure S22. ^{13}C NMR spectrum of compound **7** (125 MHz, MeOD-d_4).

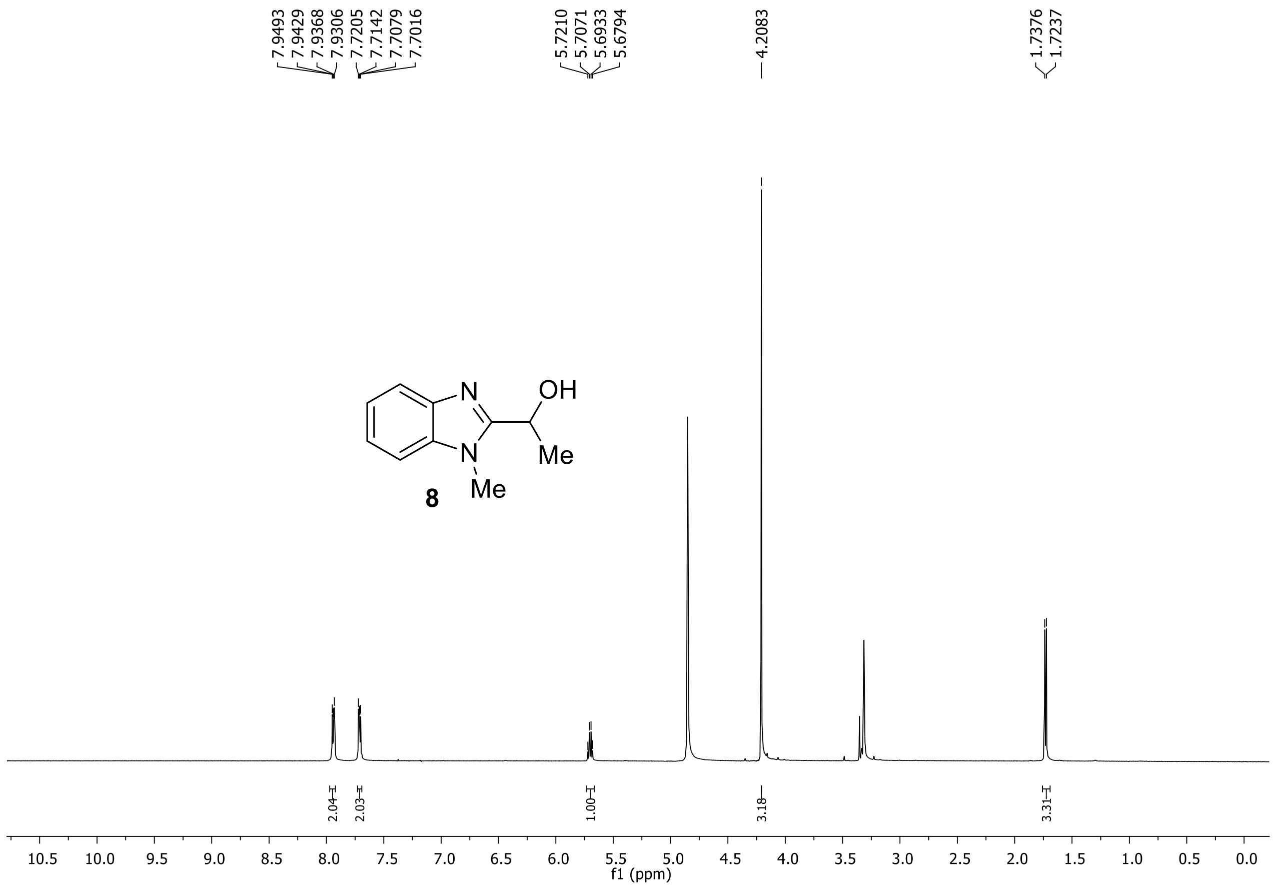

Figure S23. ^{1}H NMR spectrum of compound **8** (500 MHz, MeOD-d_4).

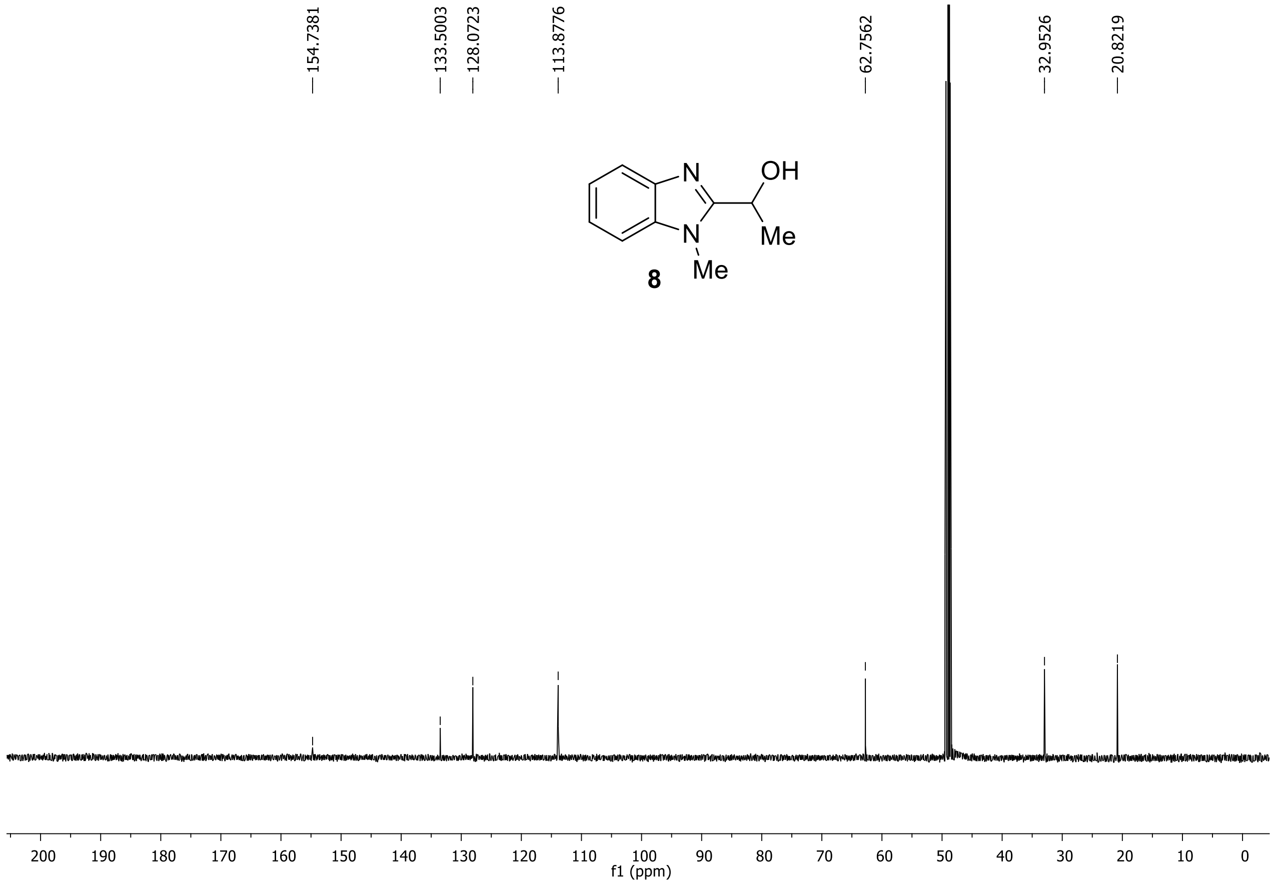

Figure S24. ^{13}C NMR spectrum of compound **8** (125 MHz, MeOD-d_4).

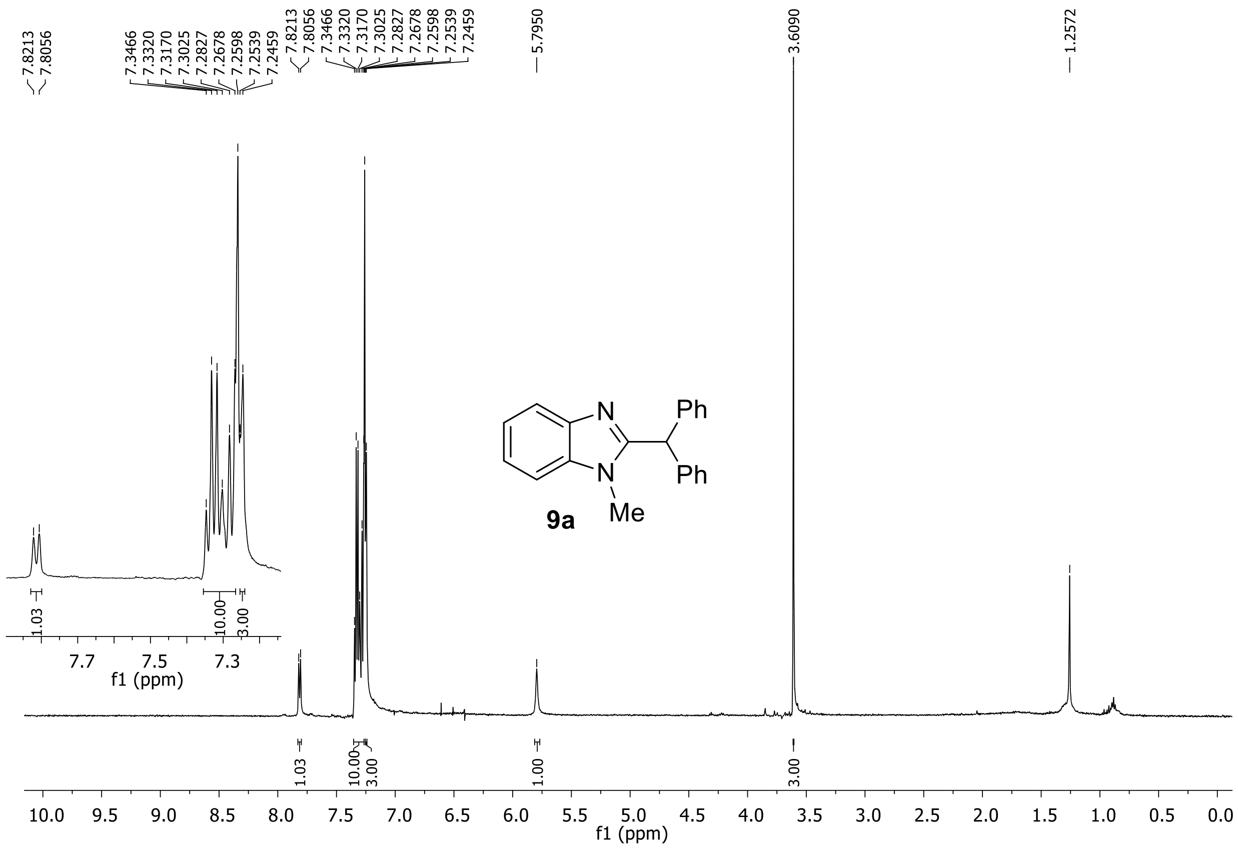

Figure S25. ^{1}H NMR spectrum of compound **9a** (500 MHz, $CDCl_3$).

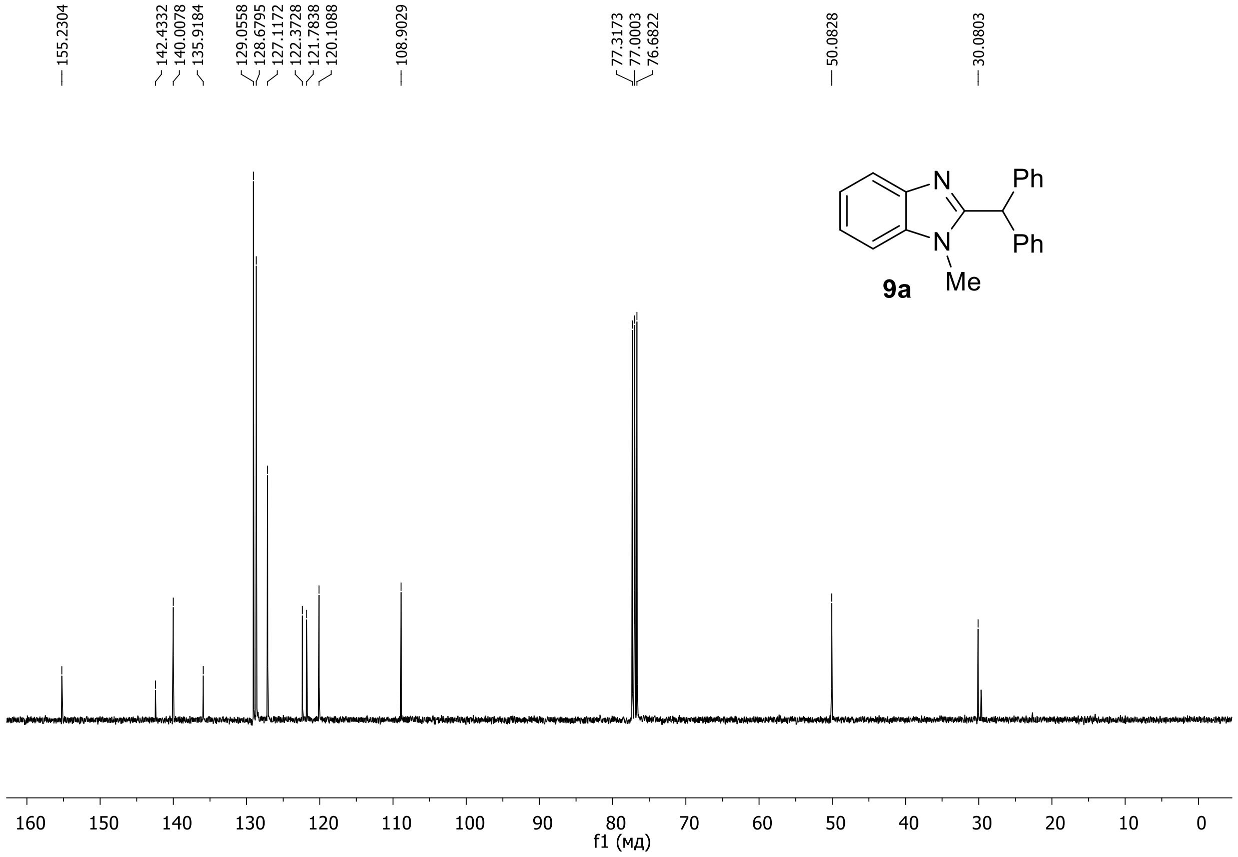

Figure S26. ^{13}C NMR spectrum of compound **9a** (125 MHz, $CDCl_3$).

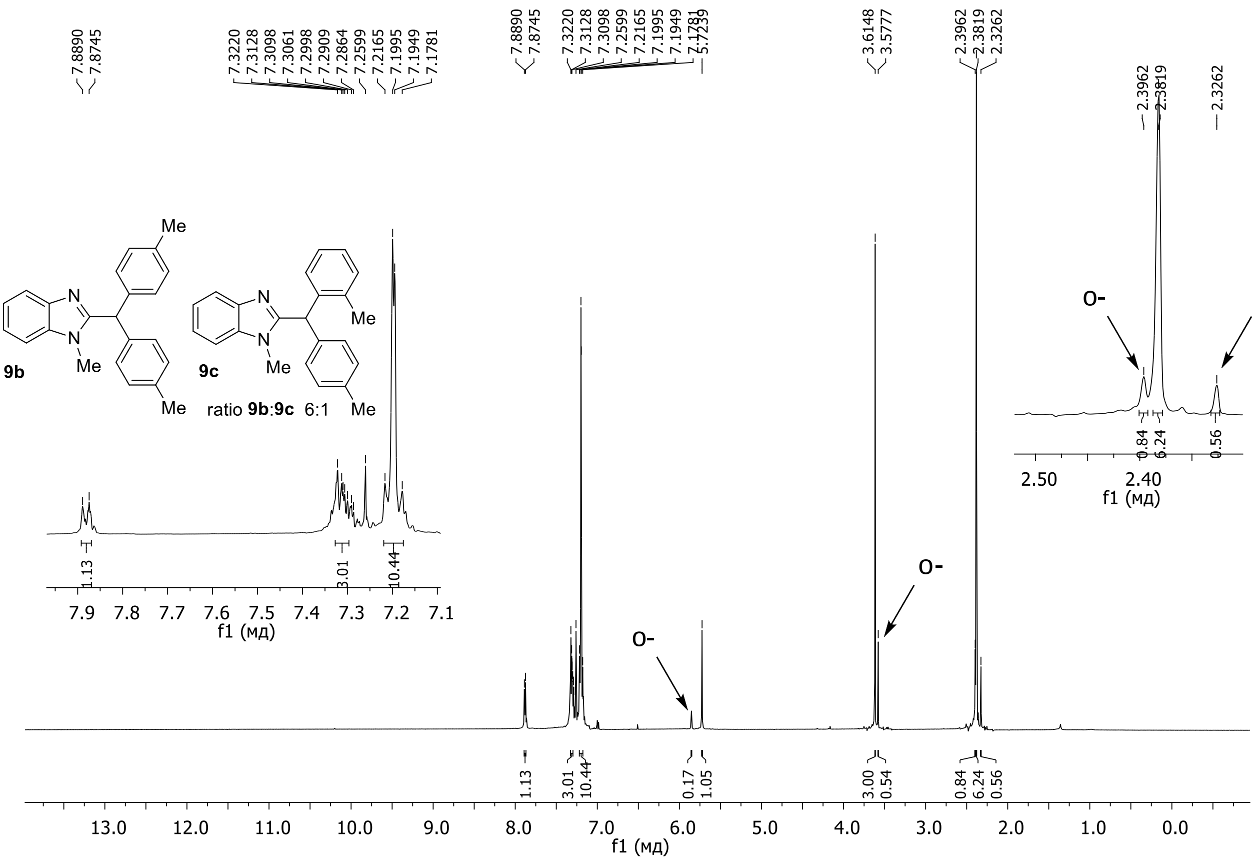

Figure S27. ^{1}H NMR spectrum of mixture of compounds **9b** and **9c** (500 MHz, $CDCl_3$).

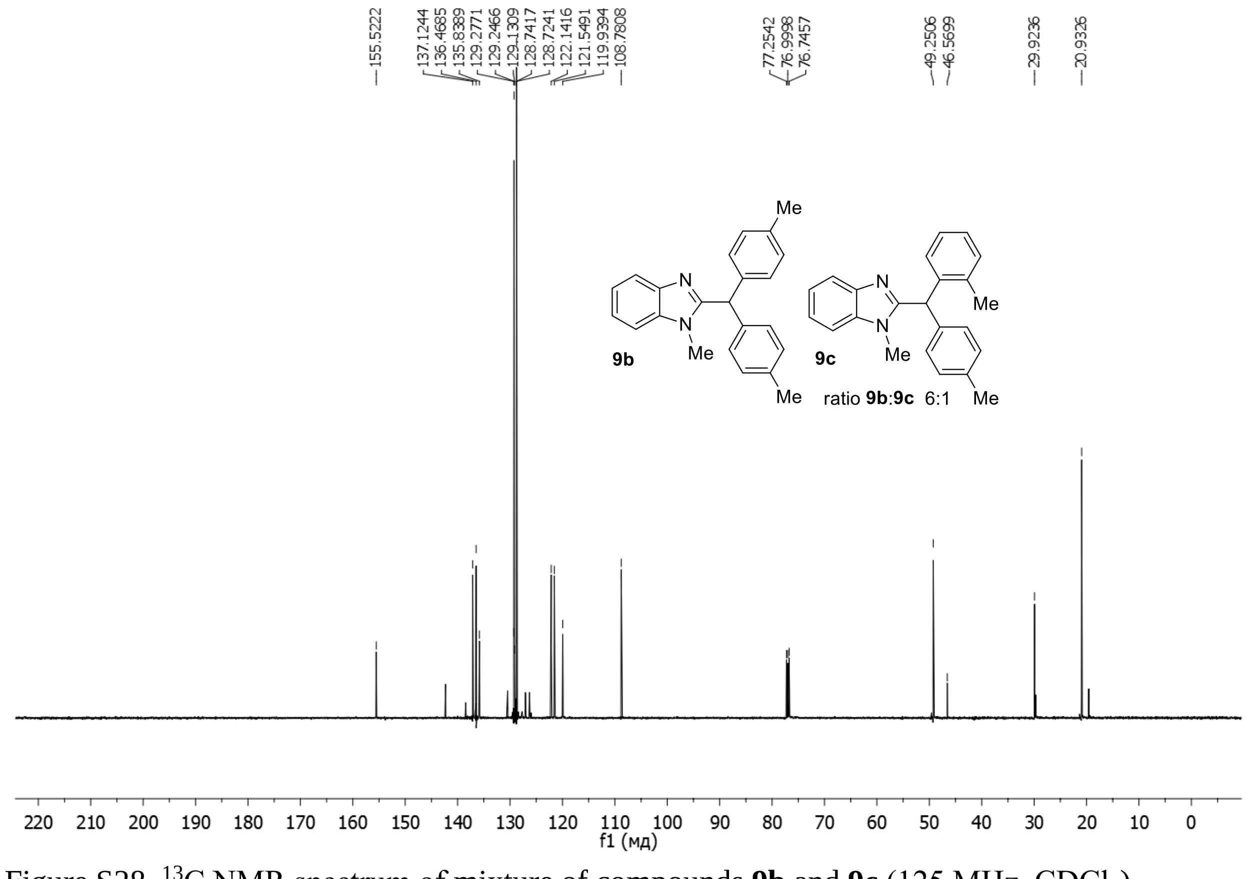

Figure S28. ^{13}C NMR spectrum of mixture of compounds **9b** and **9c** (125 MHz, $CDCl_3$).

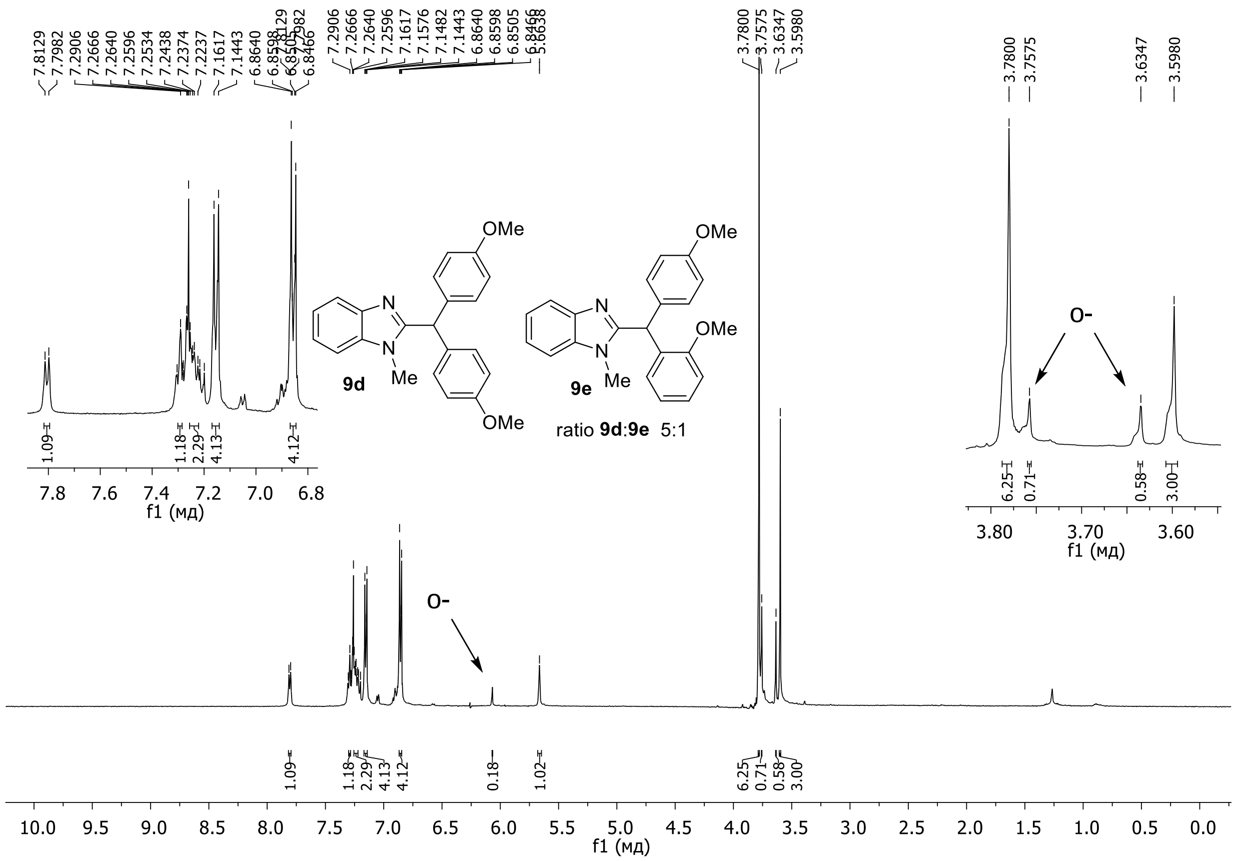

Figure S29. ^{1}H NMR spectrum of mixture of compounds **9d** and **9e** (500 MHz, $CDCl_3$).

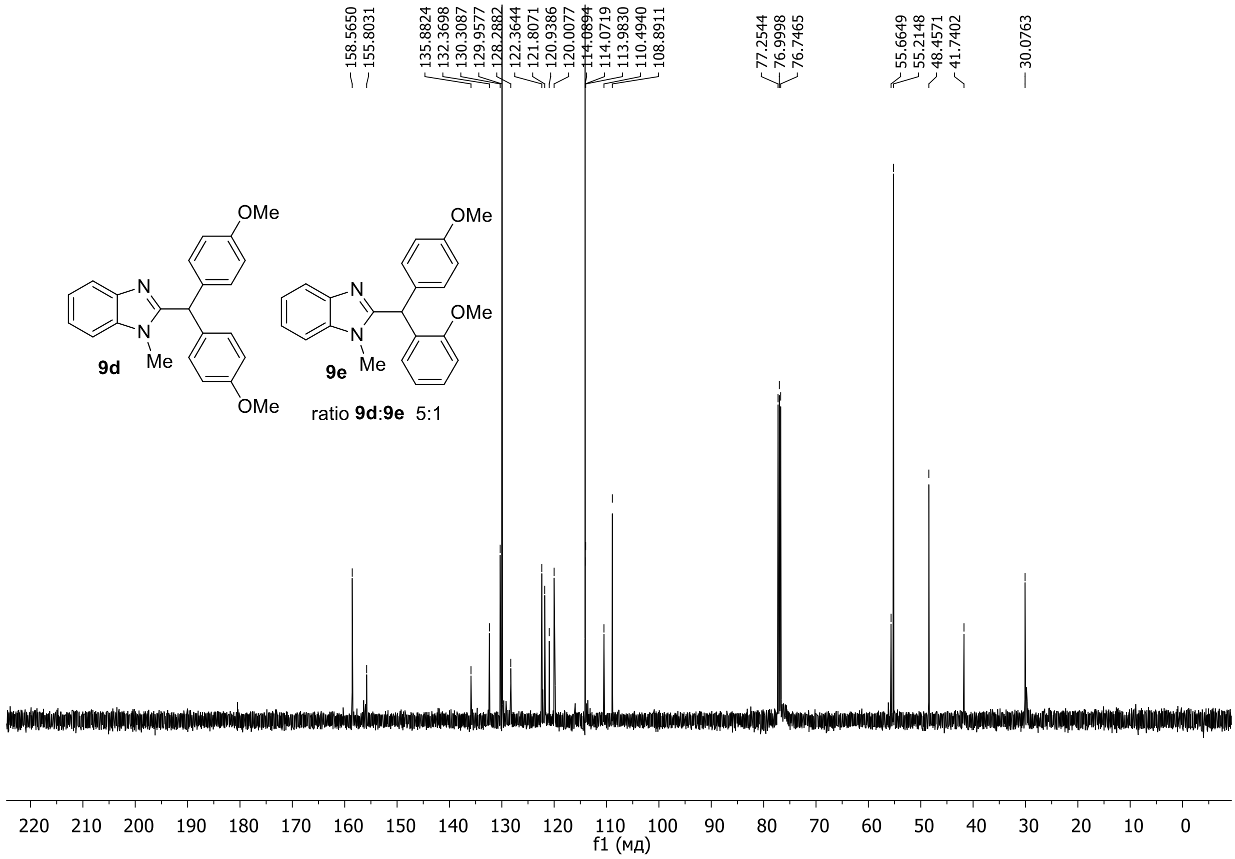

Figure S30. ^{13}C NMR spectrum of mixture of compounds **9d** and **9e** (125 MHz, $CDCl_3$).

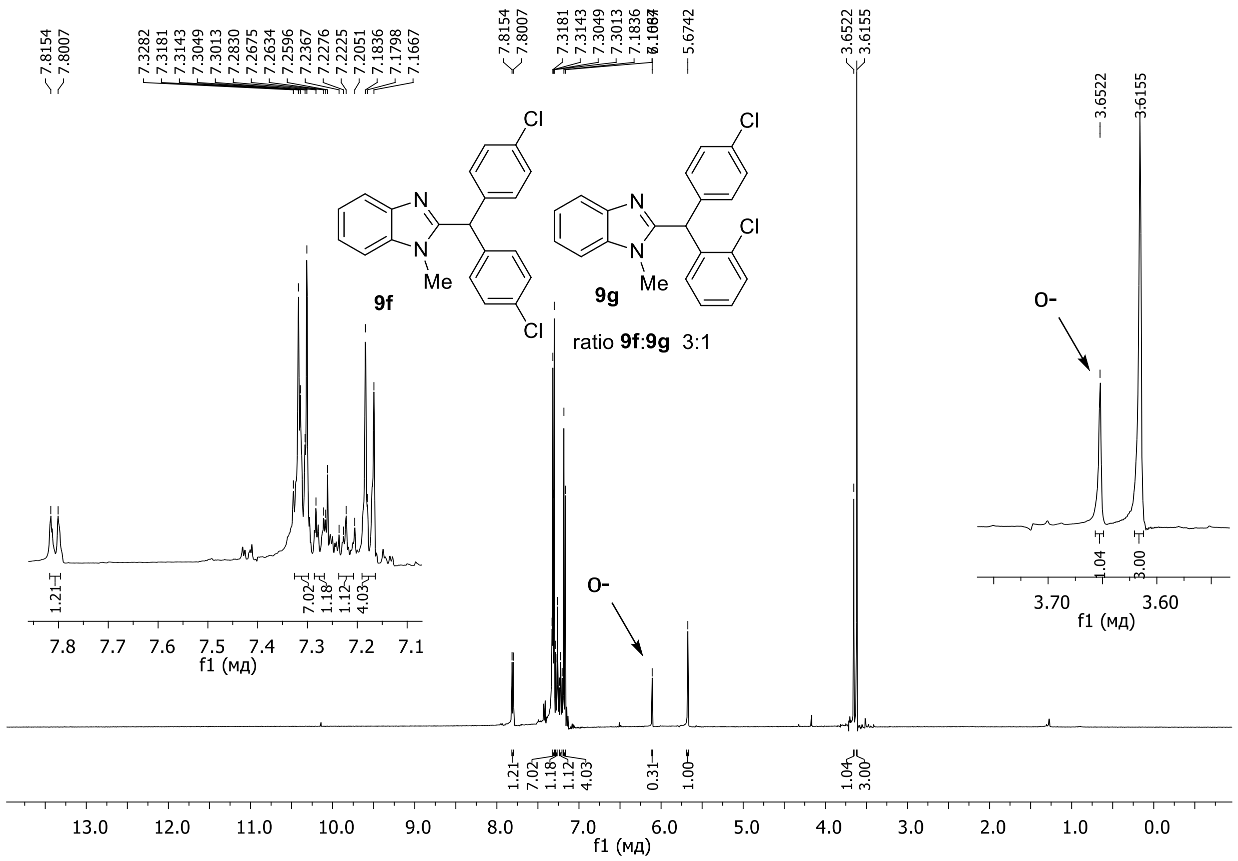

Figure S31. ^{1}H NMR spectrum of mixture of compounds **9f** and **9g** (500 MHz, $CDCl_3$).

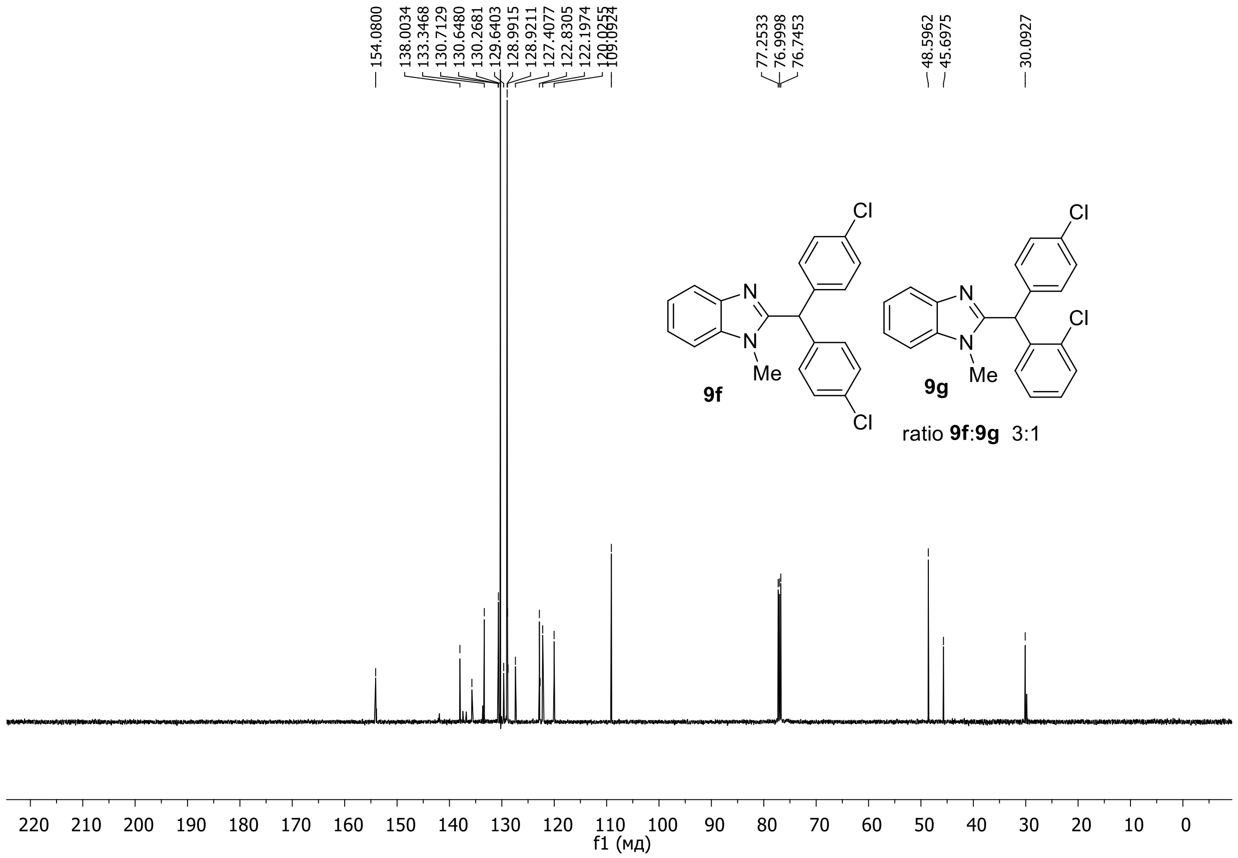

Figure S32. ^{13}C NMR spectrum of mixture of compounds **9f** and **9g** (125 MHz, $CDCl_3$).

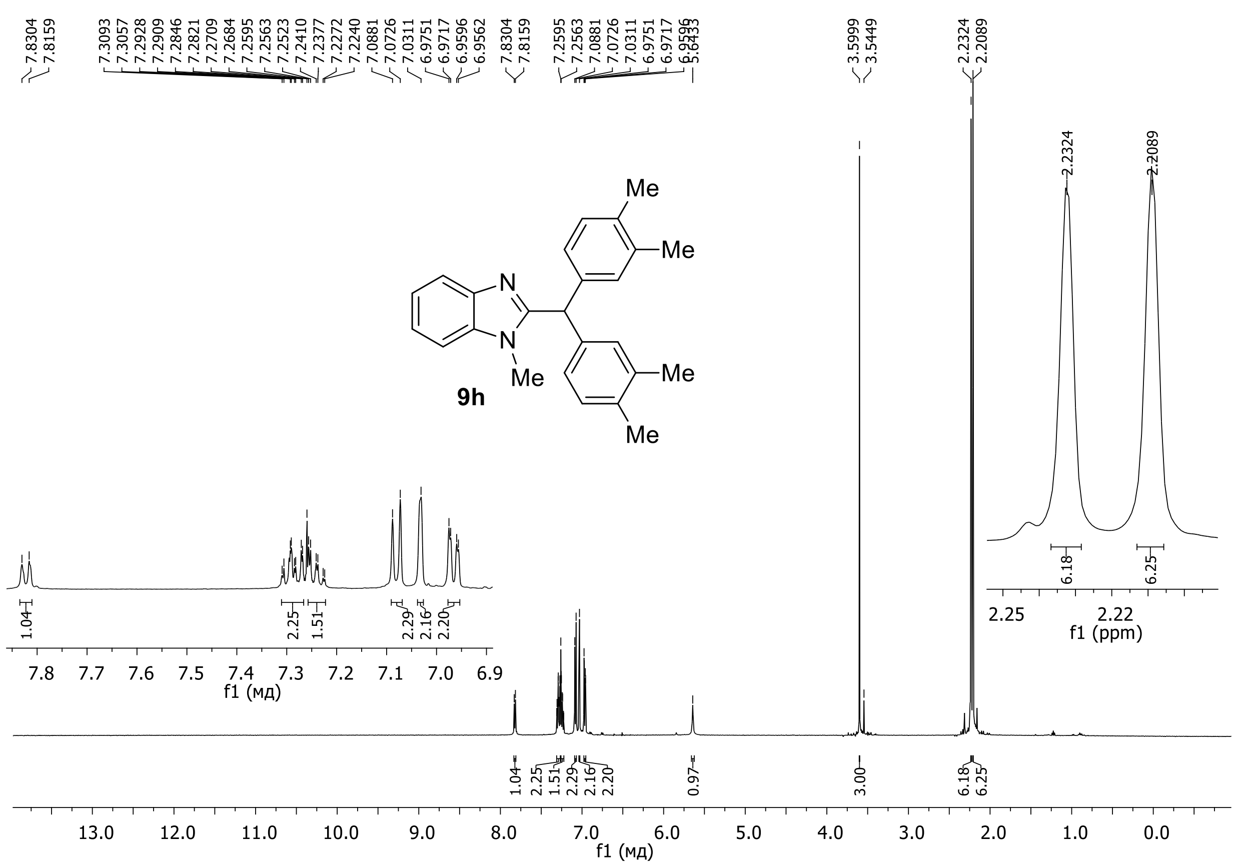

Figure S33. ^{1}H NMR spectrum of compound **9h** (500 MHz, $CDCl_3$).

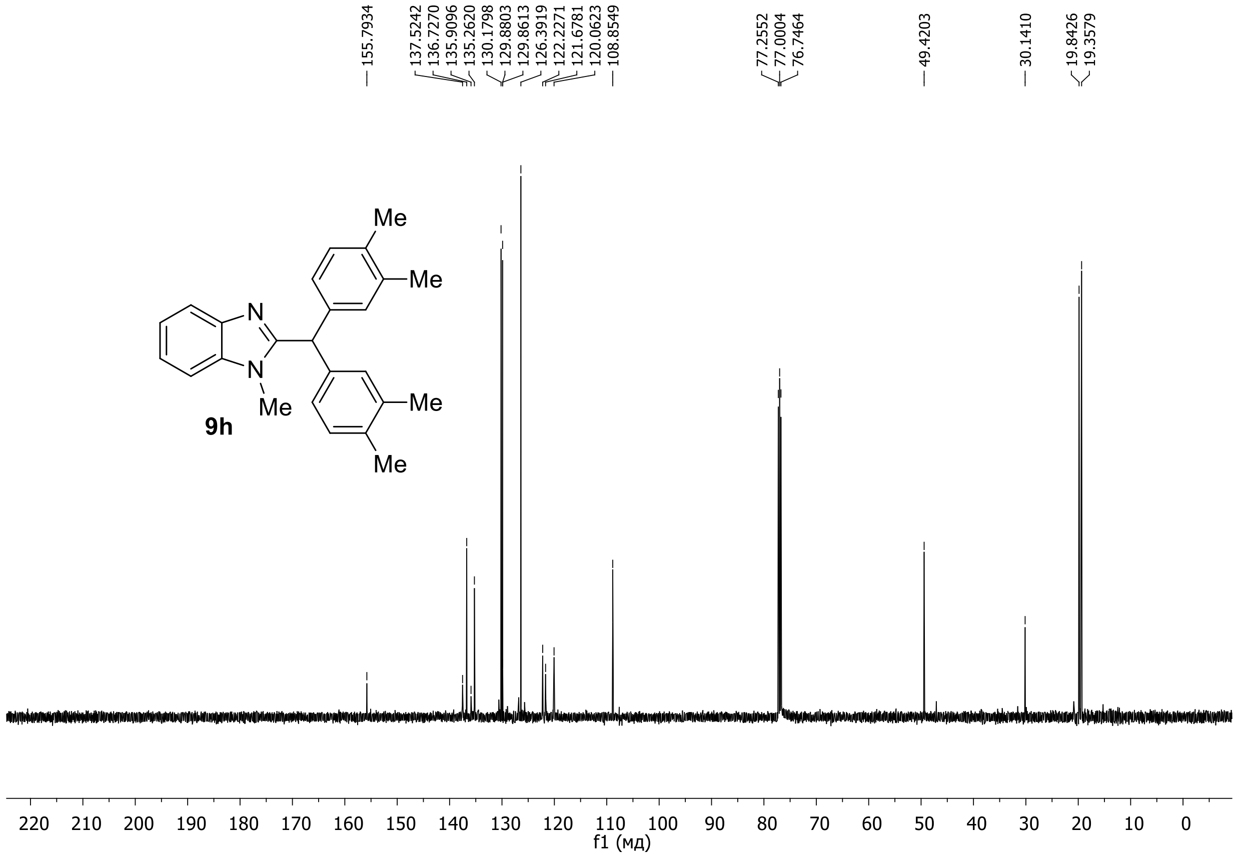

Figure S34. ^{13}C NMR spectrum of compound **9h** (125 MHz, $CDCl_3$).

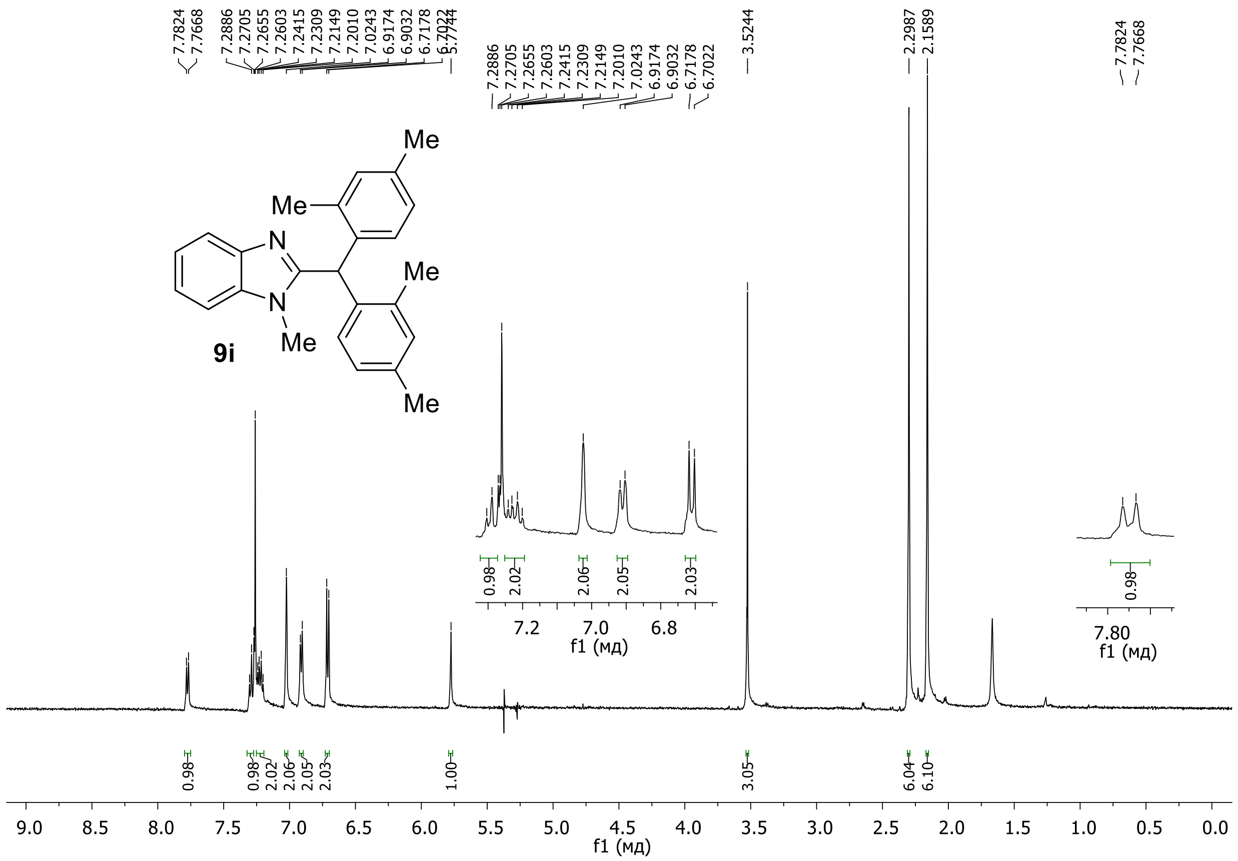

Figure S35. ^{1}H NMR spectrum of compound **9i** (500 MHz, $CDCl_3$).

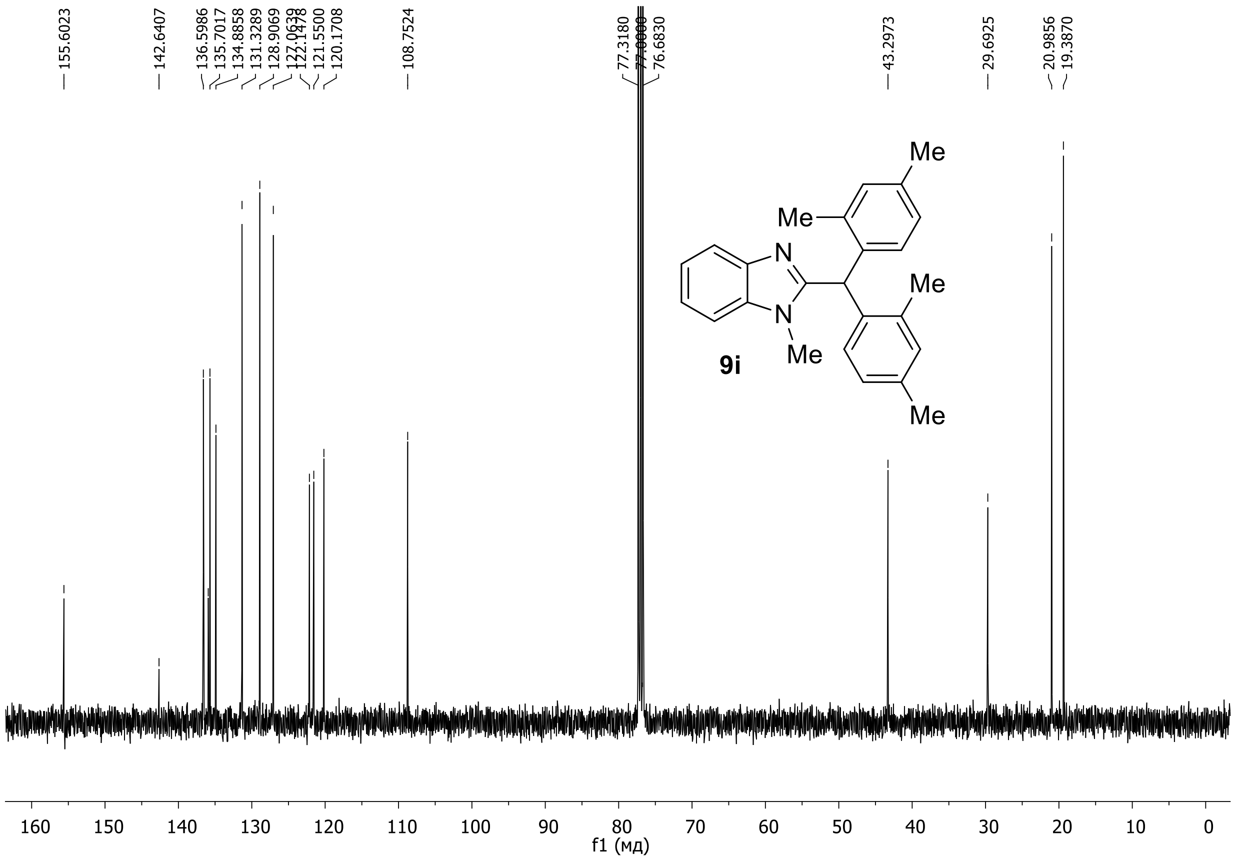

Figure S36. ^{13}C NMR spectrum of compound **9i** (125 MHz, $CDCl_3$).

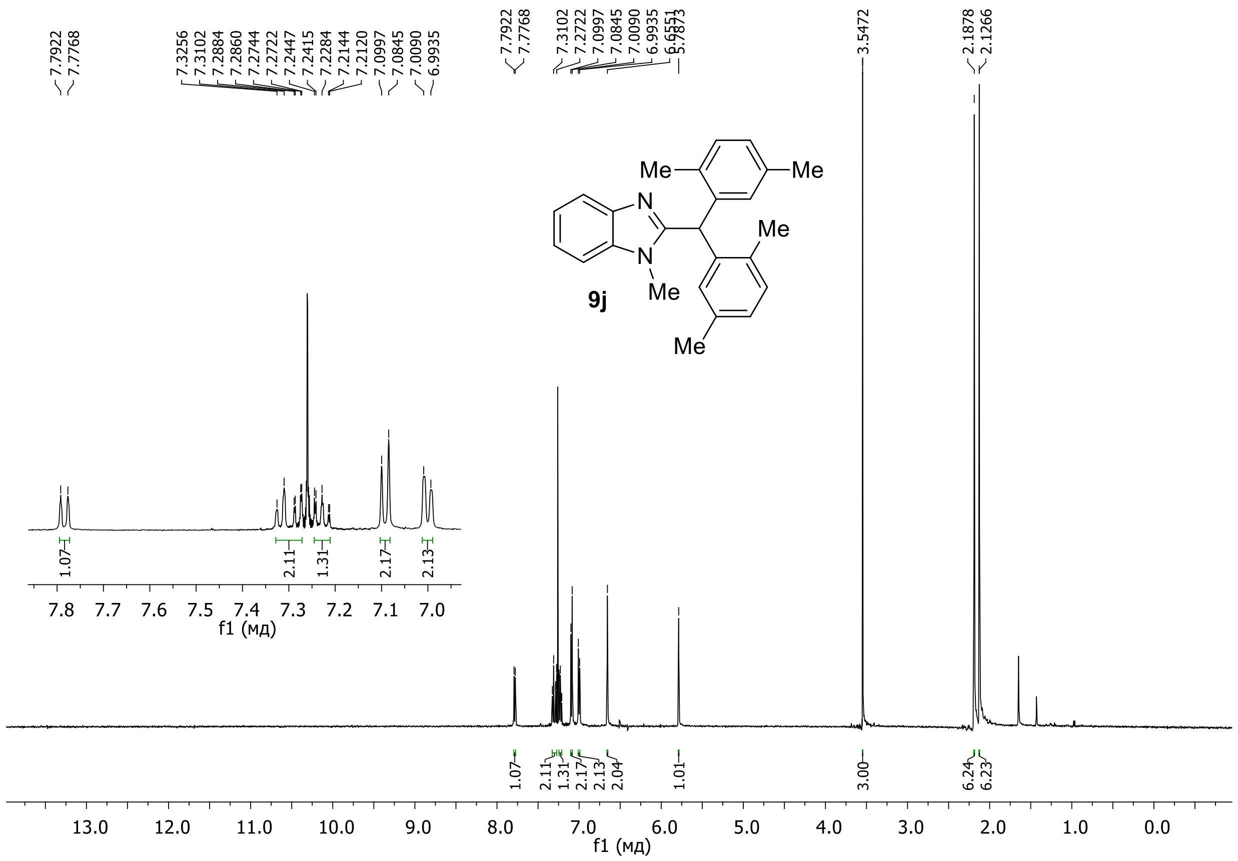

Figure S37. ^{1}H NMR spectrum of compound **9j** (500 MHz, $CDCl_3$).

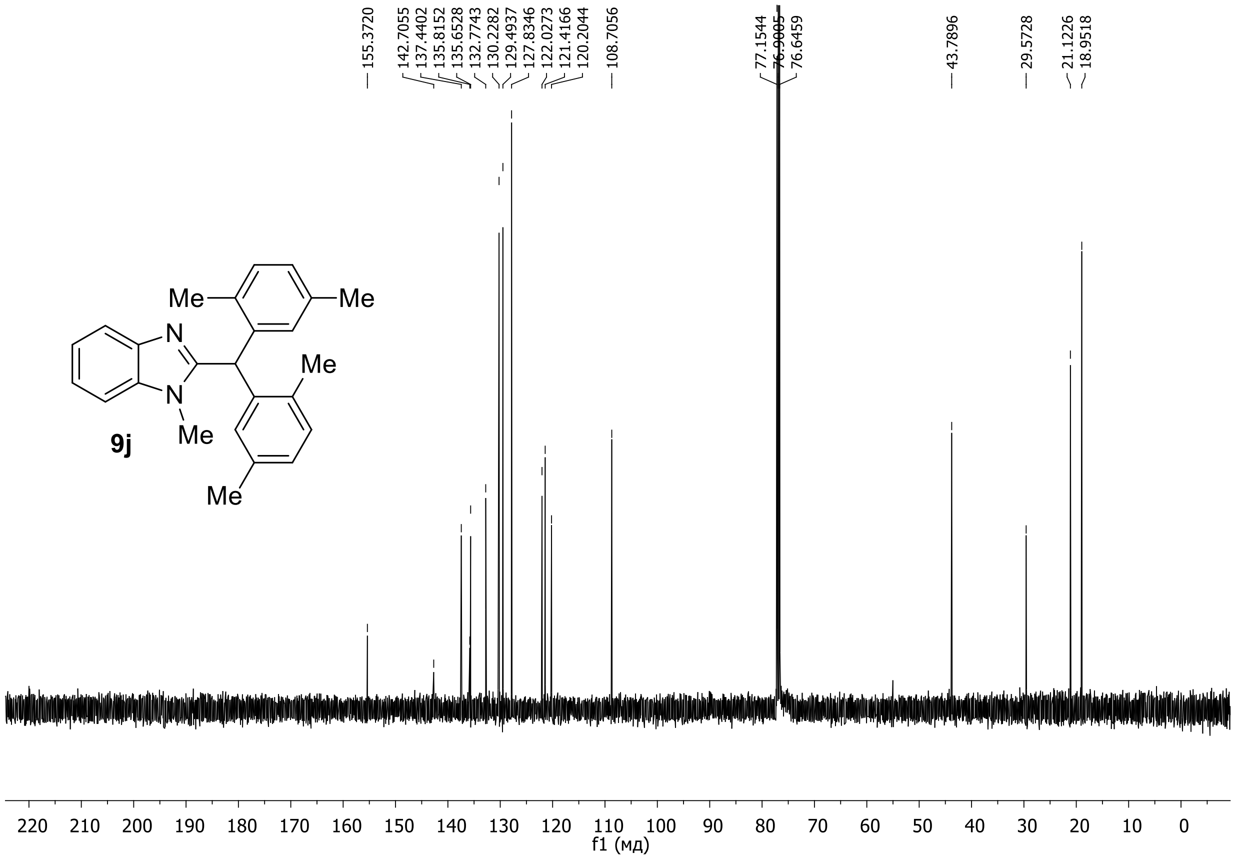

Figure S38. ^{13}C NMR spectrum of compound **9j** (125 MHz, $CDCl_3$).

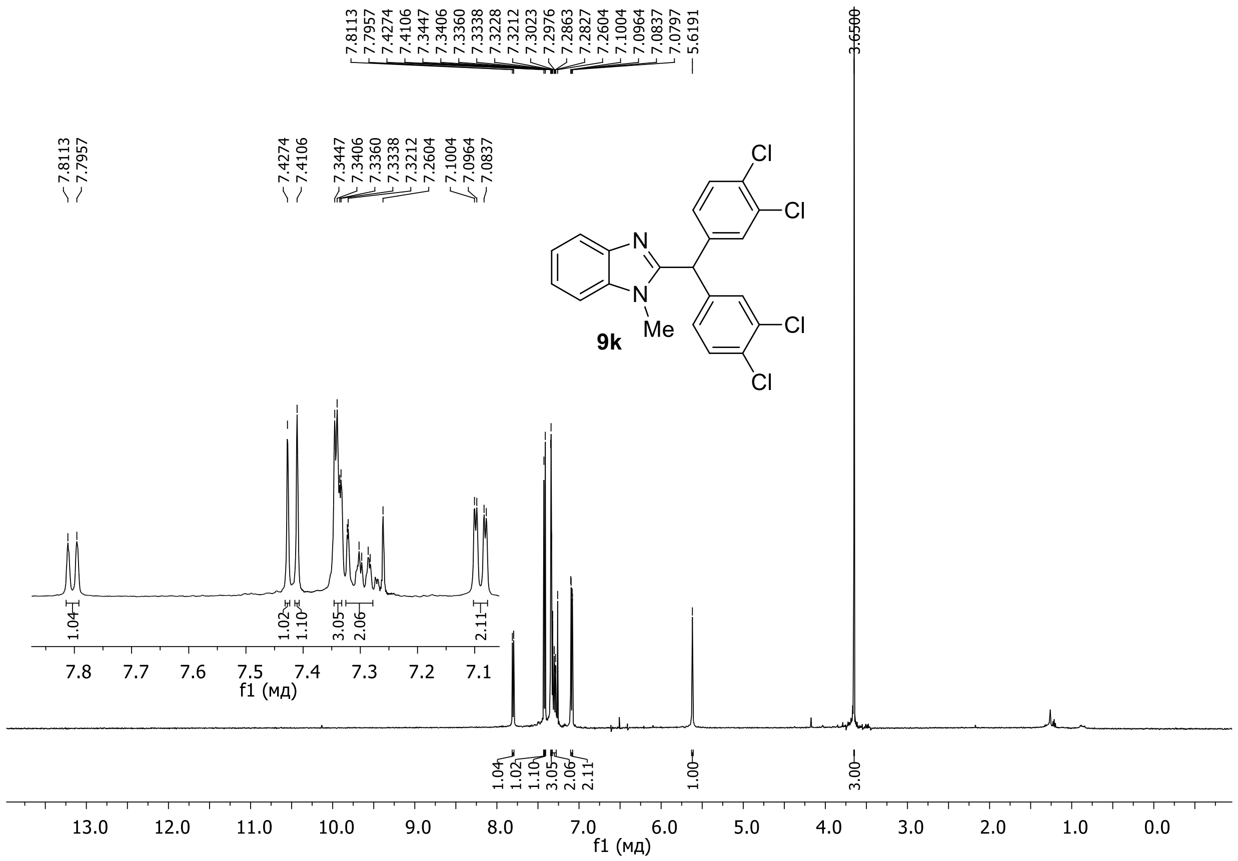

Figure S39. ^{1}H NMR spectrum of compound **9k** (500 MHz, $CDCl_3$).

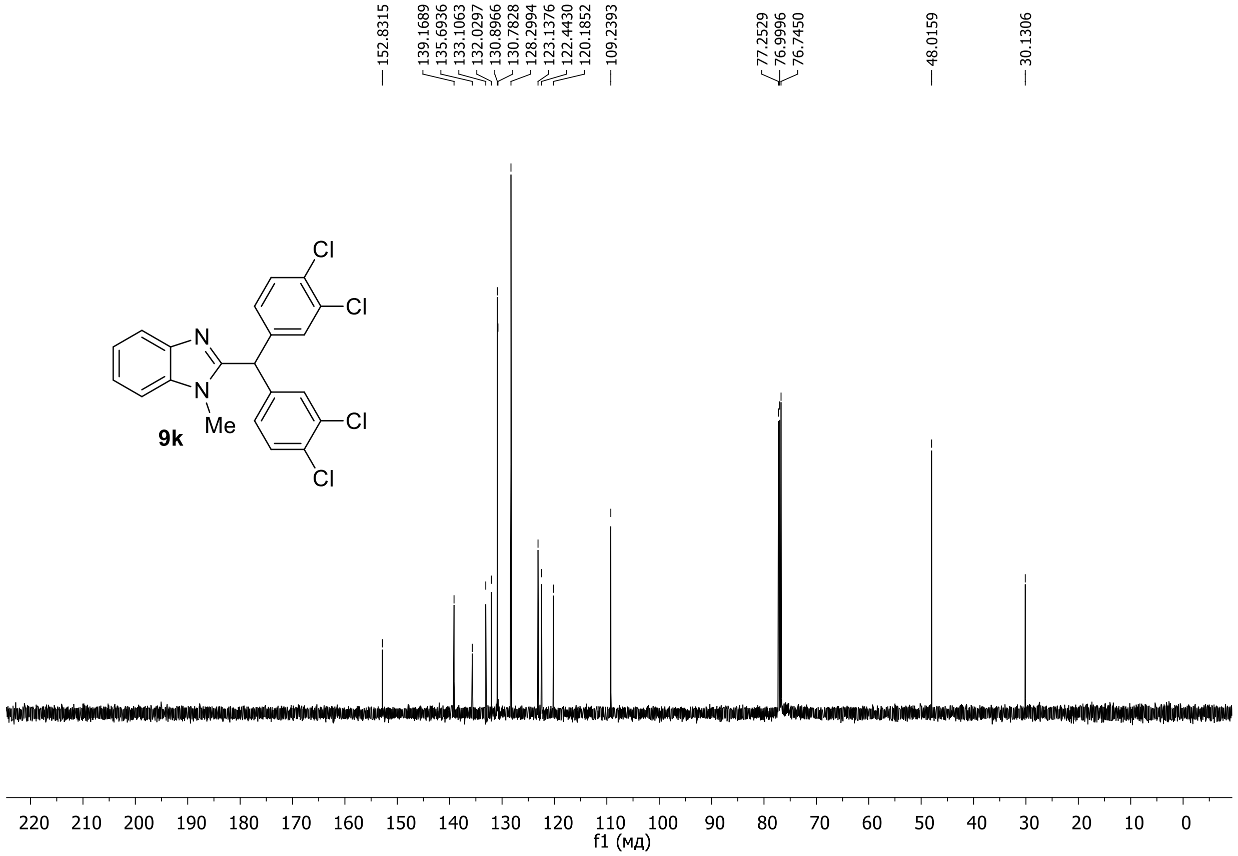

Figure S40. ^{13}C NMR spectrum of compound **9k** (125 MHz, $CDCl_3$).

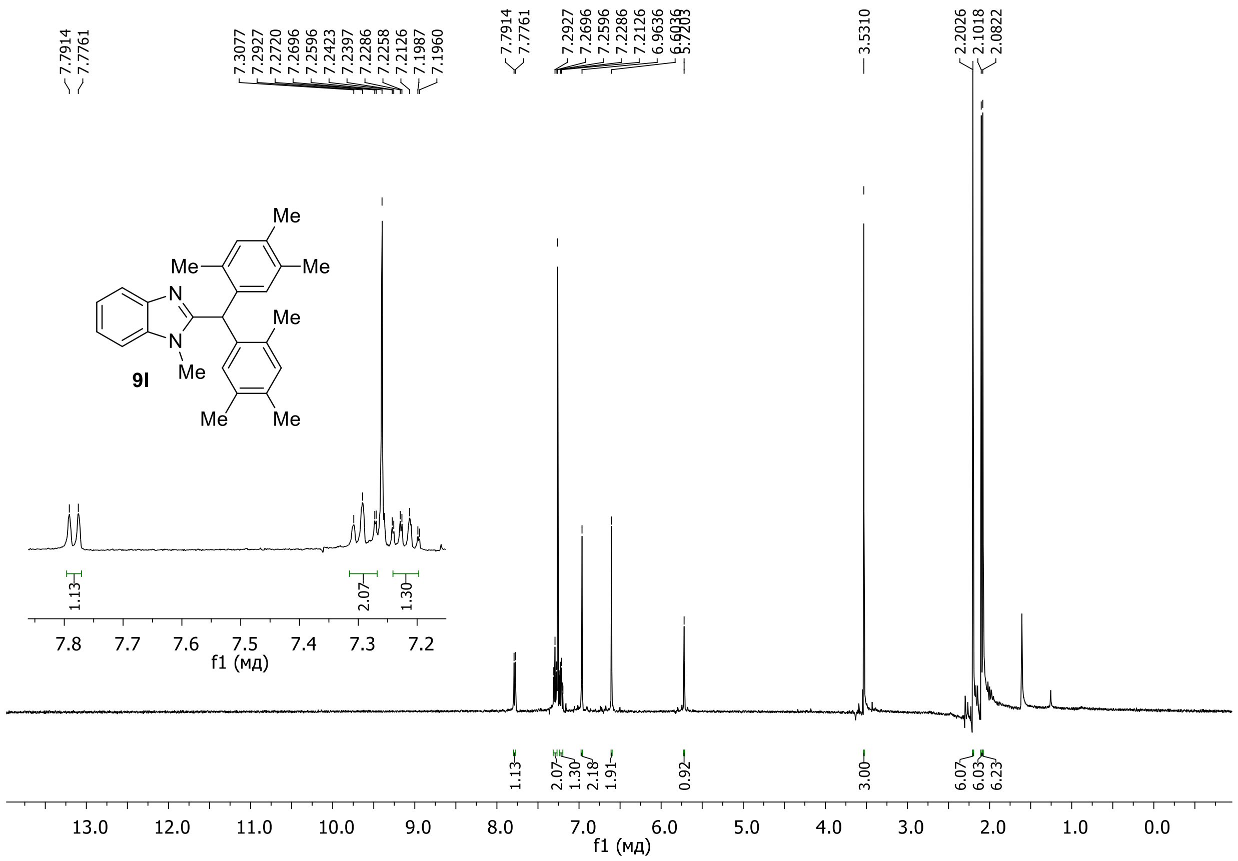

Figure S41. ^{1}H NMR spectrum of compound **9l** (500 MHz, $CDCl_3$).

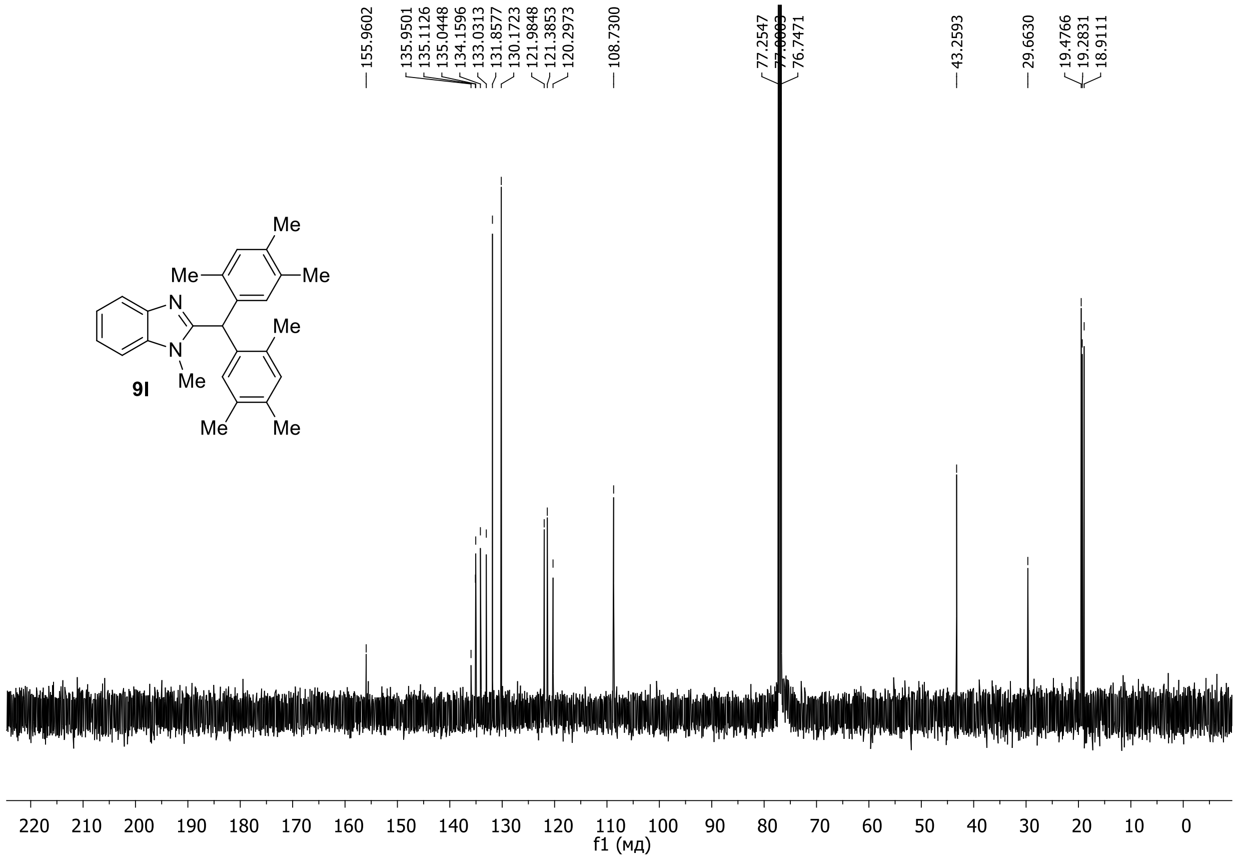

Figure S42. ^{13}C NMR spectrum of compound **9l** (125 MHz, $CDCl_3$).

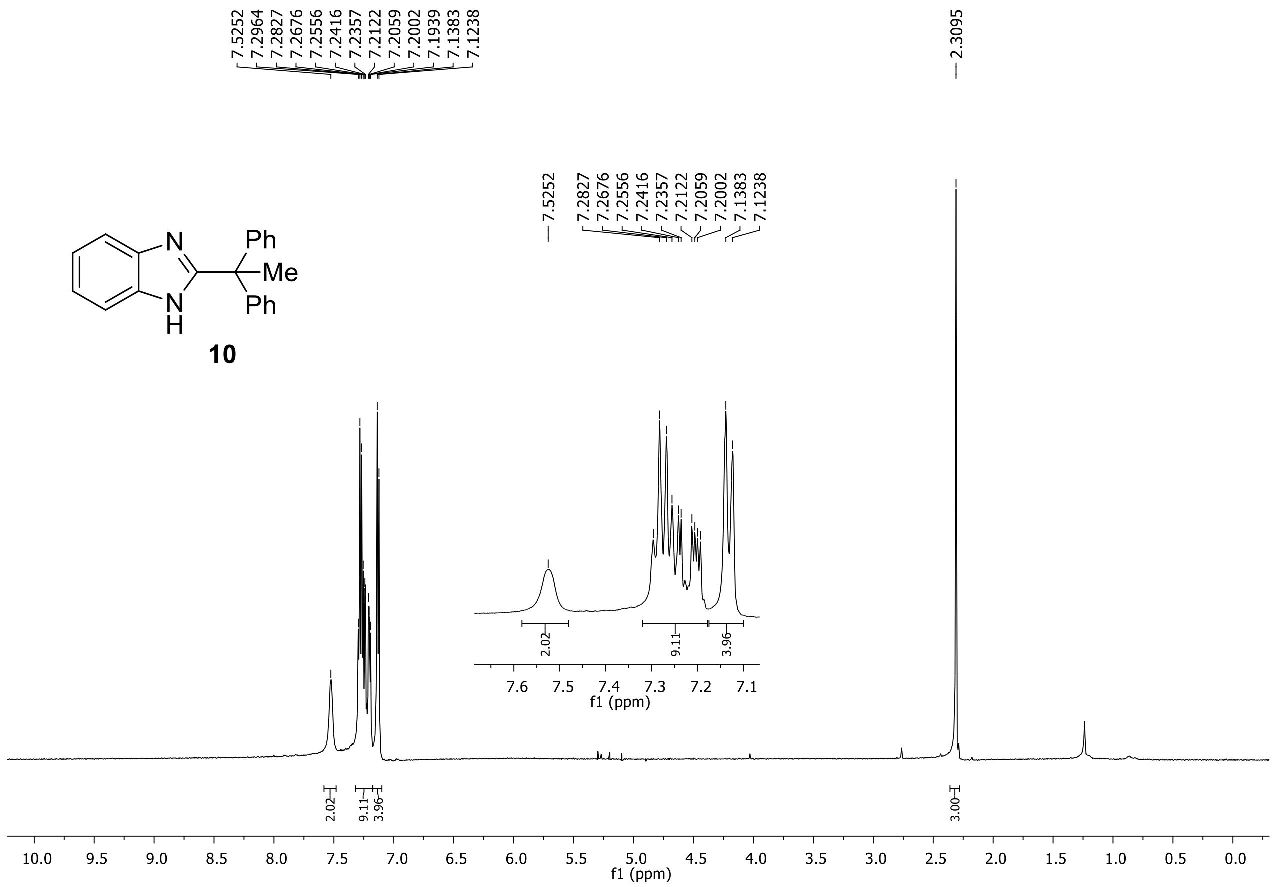

Figure S43. ^{1}H NMR spectrum of compound **10** (500 MHz, $CDCl_3$).

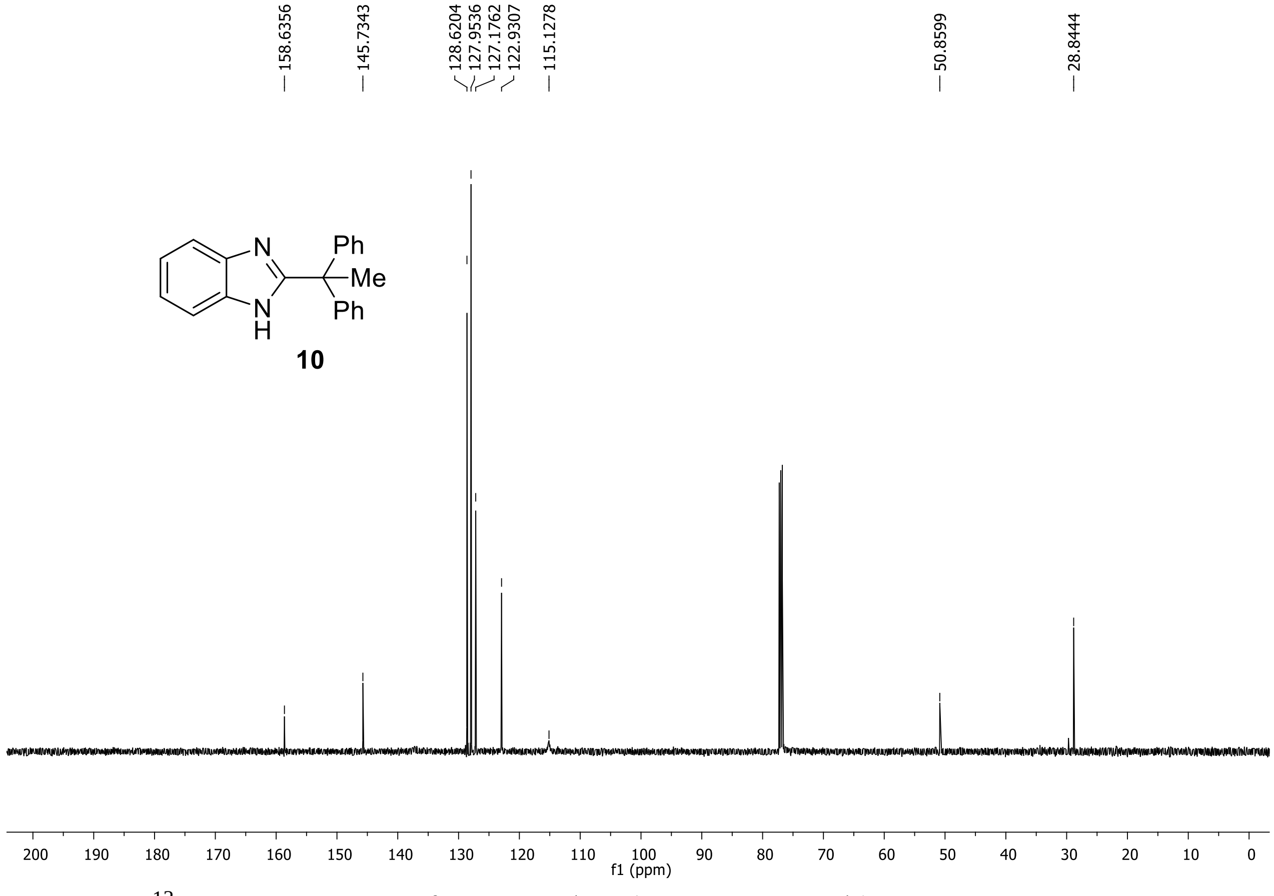

Figure S44. ^{13}C NMR spectrum of compound **10** (125 MHz, $CDCl_3$).

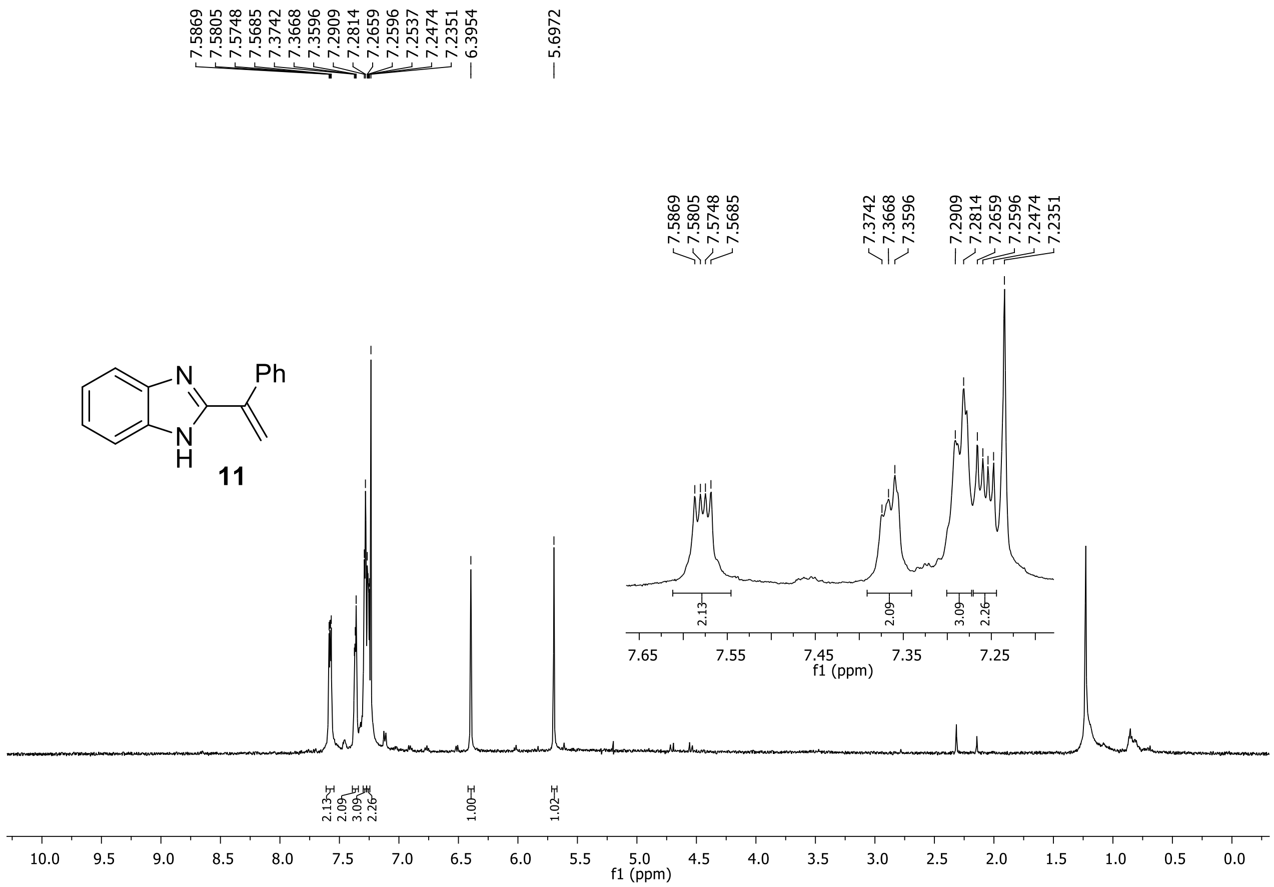

Figure S45. ^{1}H NMR spectrum of compound **11** (500 MHz, $CDCl_3$).

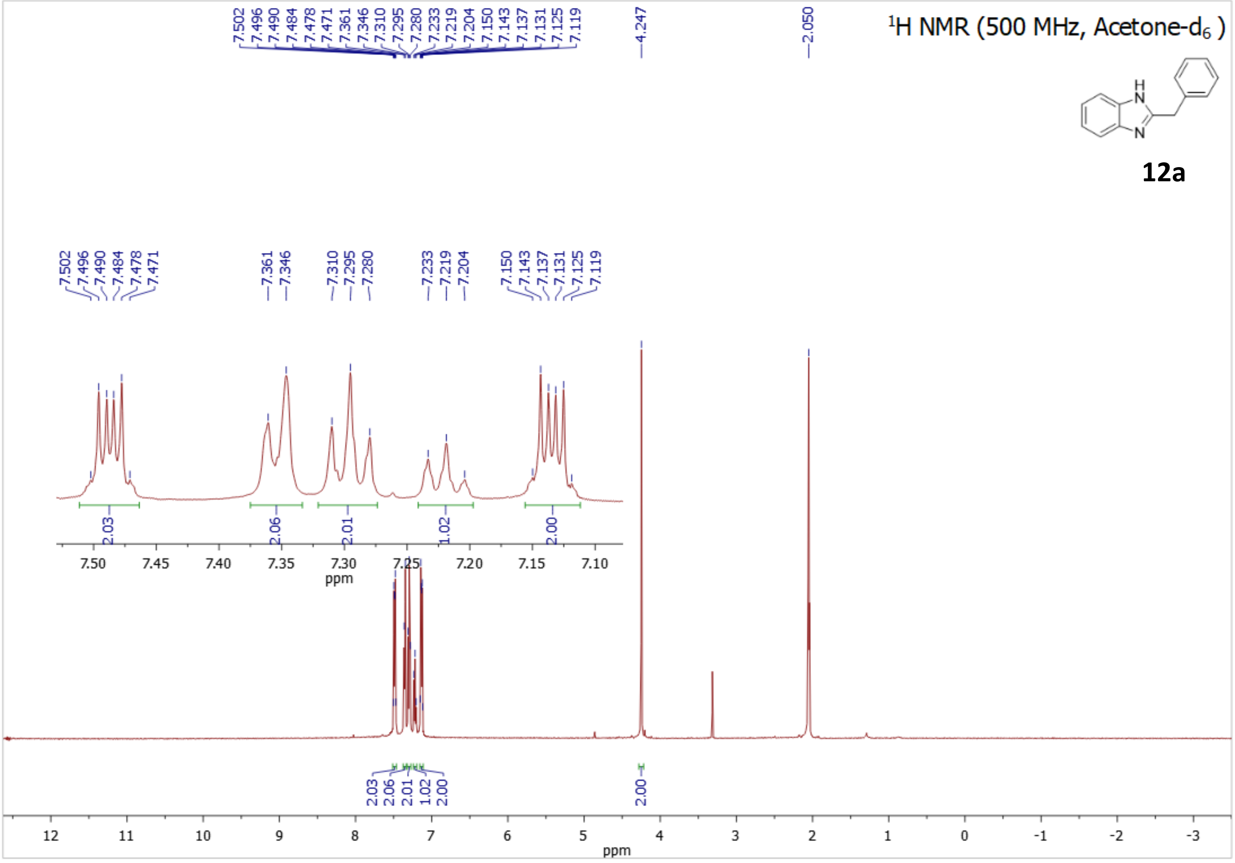

Figure S46. ^{1}H NMR spectrum of compound **12a** (500 MHz, $(CD_3)_2CO$).

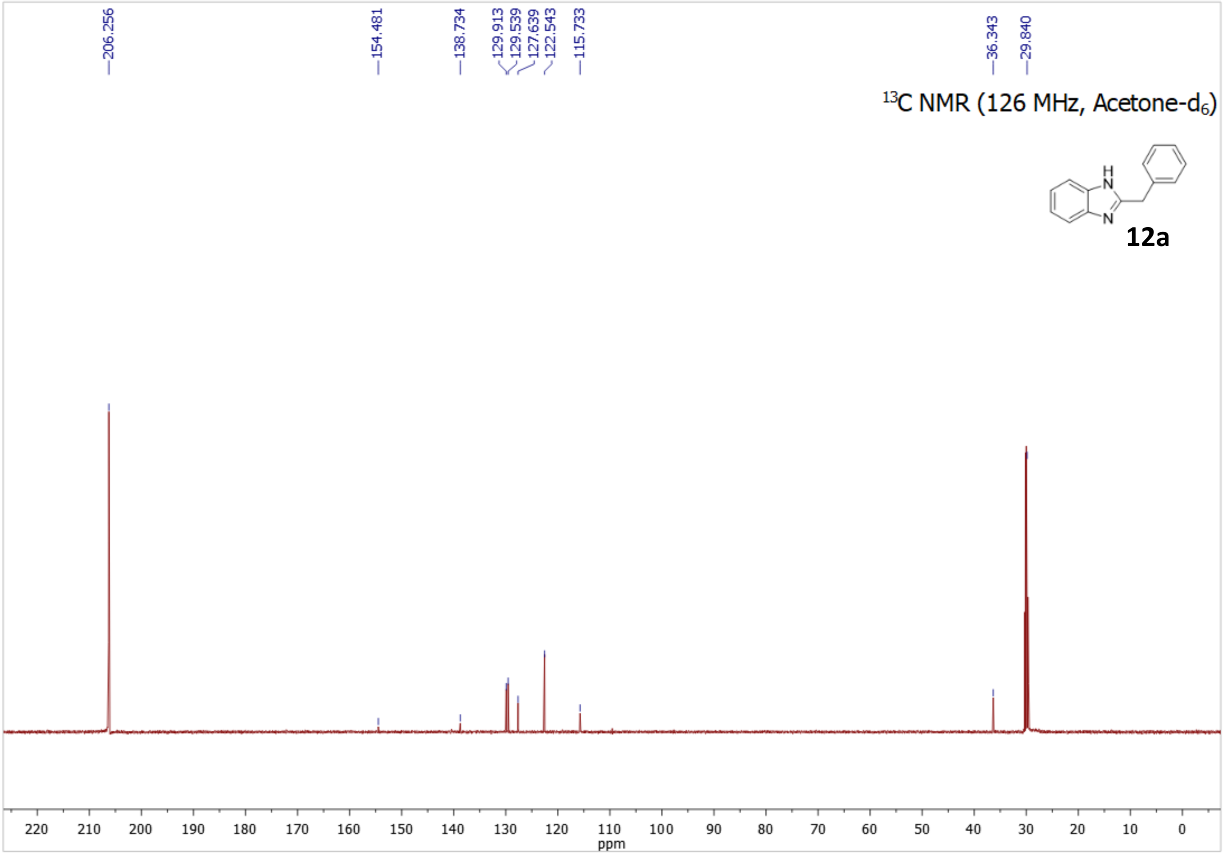

Figure S47. ^{13}C NMR spectrum of compound **12a** (125 MHz, $(CD_3)_2CO$).

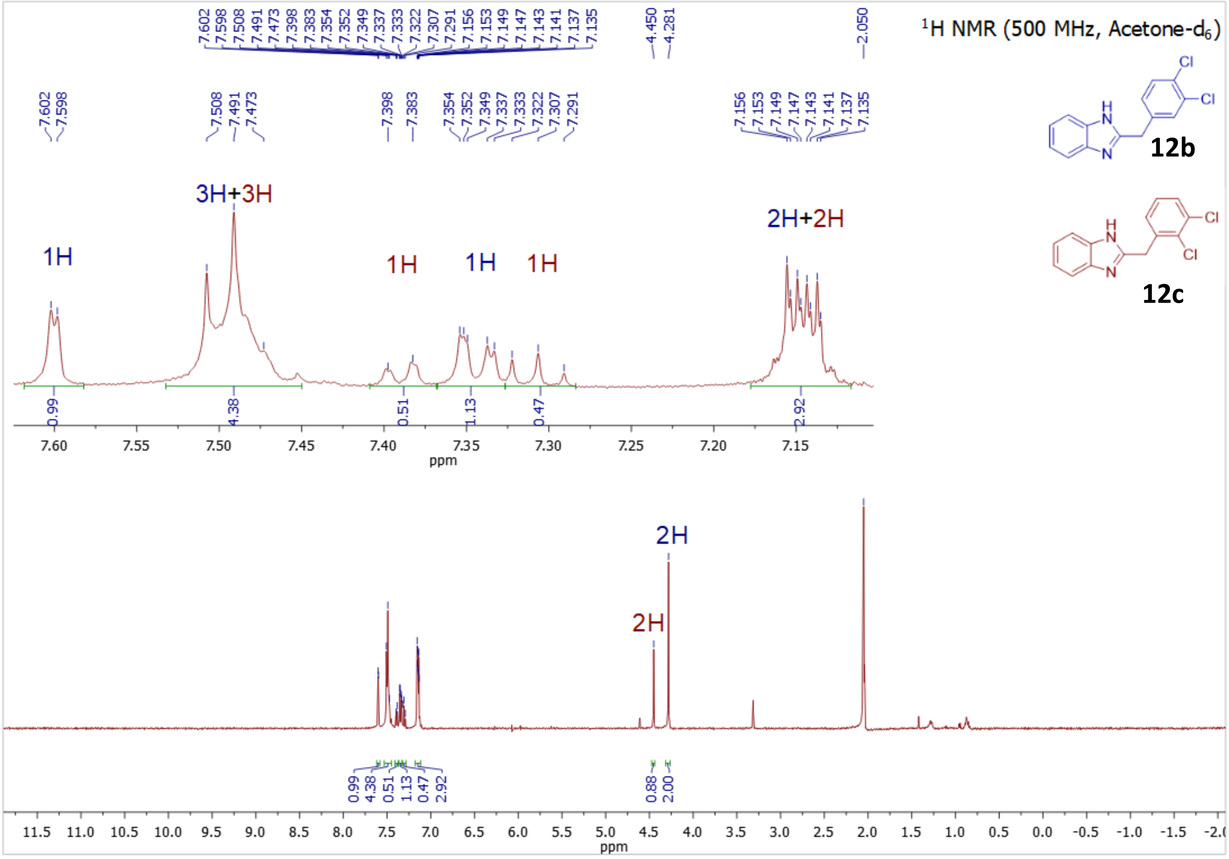

Figure S48. ^{1}H NMR spectrum of mixture of compounds **12b** and **12c** (500 MHz, $(CD_3)_2CO$).

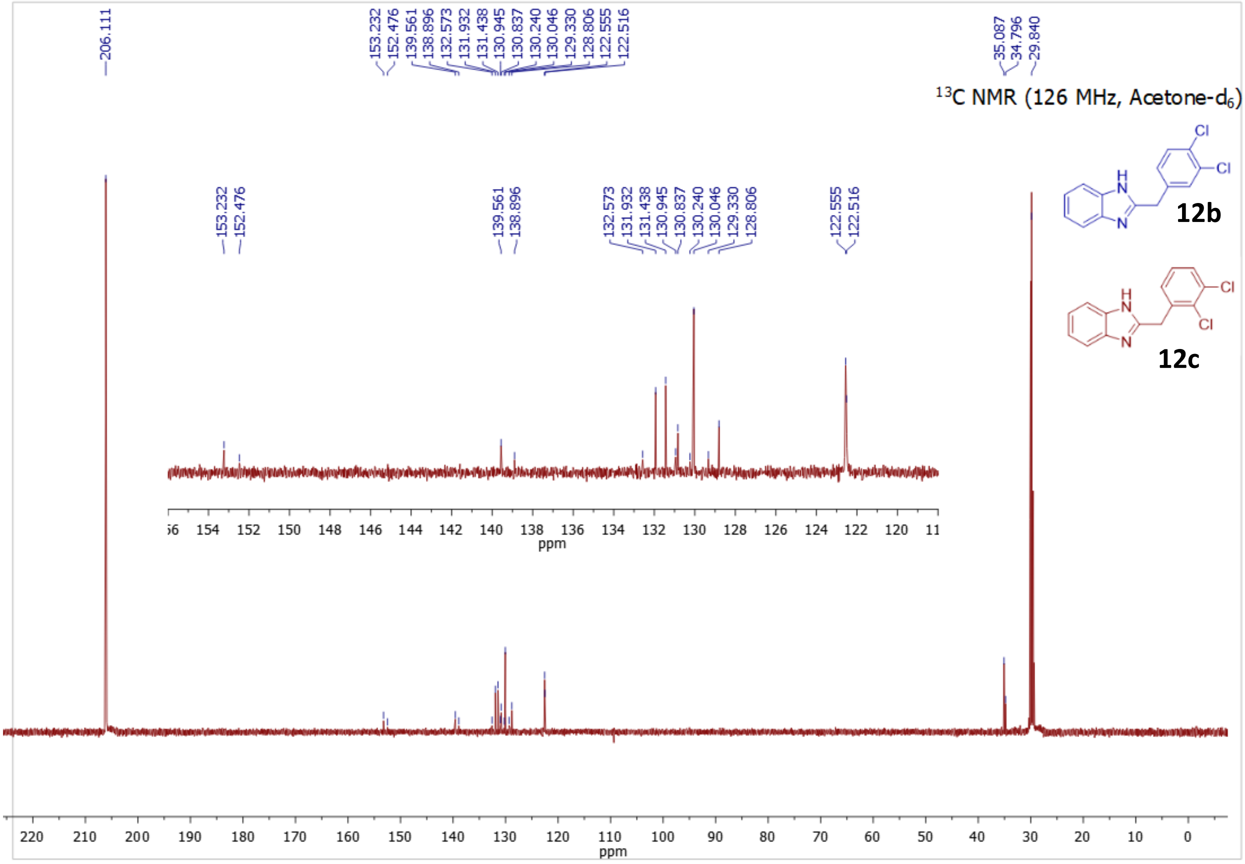

Figure S49. ^{13}C NMR spectrum of mixture of compounds **12b** and **12c** (125 MHz, $(CD_3)_2CO$).

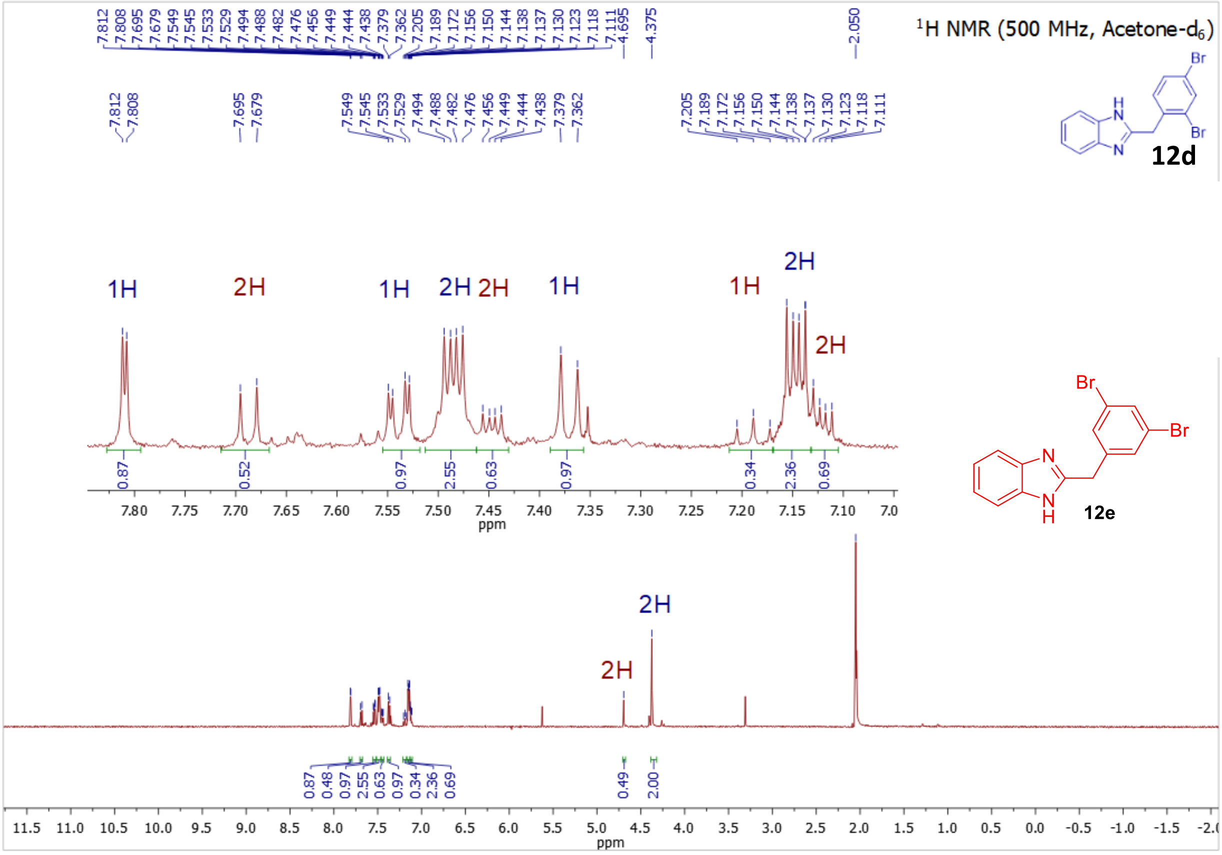

Figure S50. ^{1}H NMR spectrum of mixture of compounds **12d** and **12e** (500 MHz, $(CD_3)_2CO$).

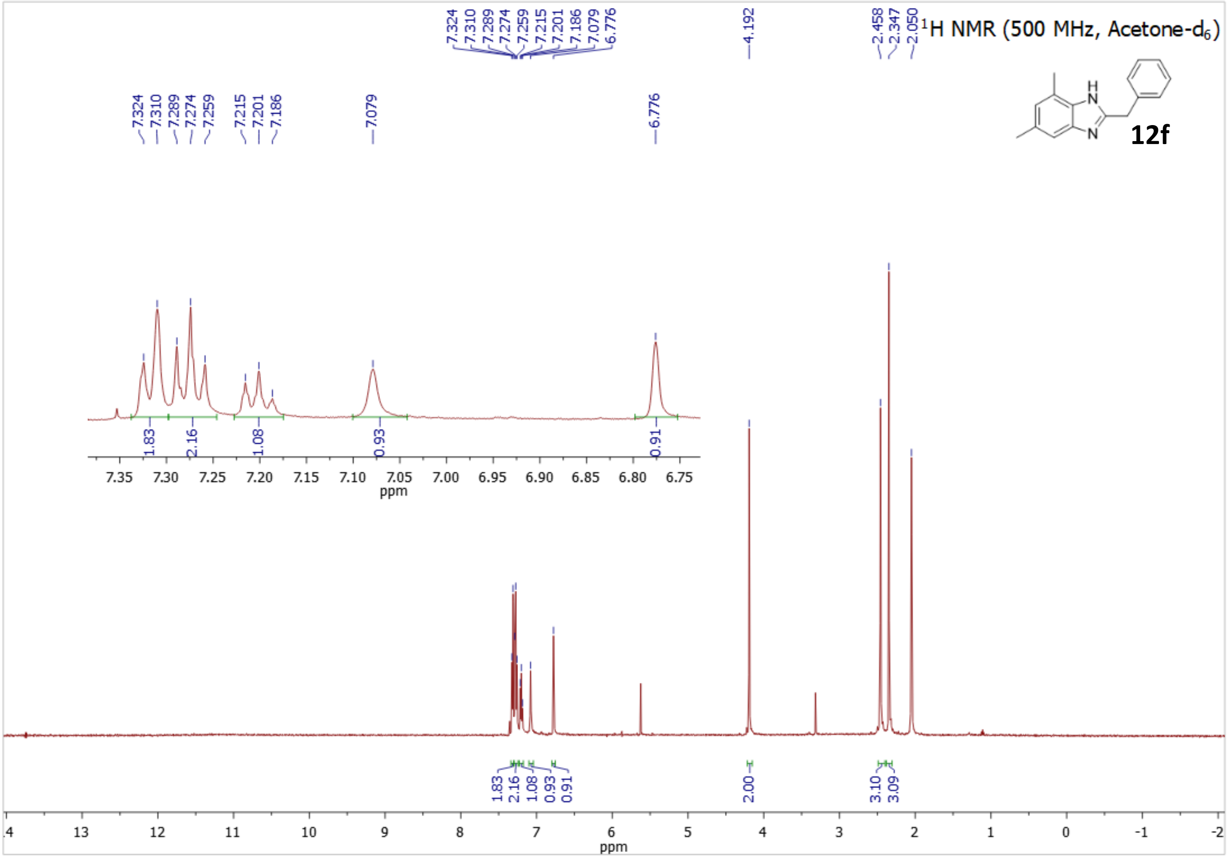

Figure S51. ^{1}H NMR spectrum of compound **12f** (500 MHz, $(CD_3)_2CO$).

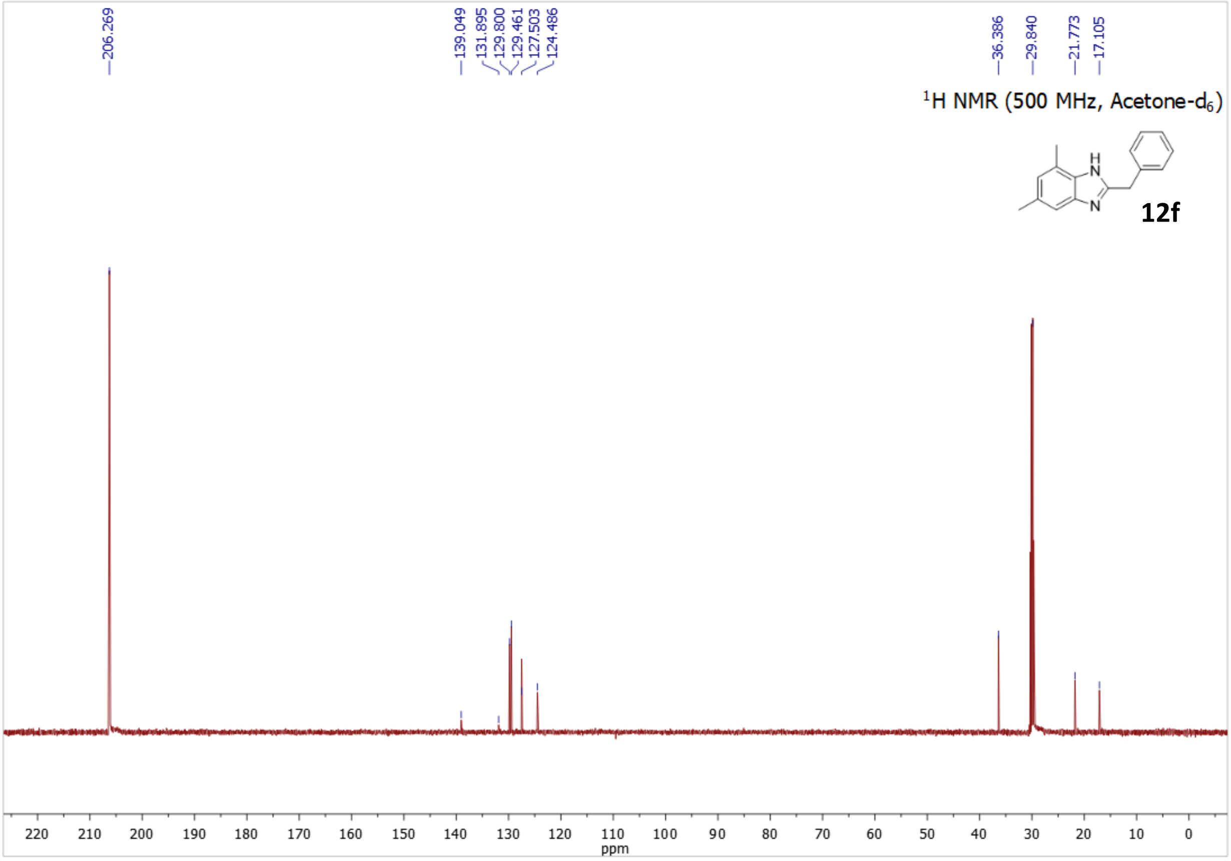

Figure S52. ^{13}C NMR spectrum of compound **12f** (125 MHz, $(CD_3)_2CO$).

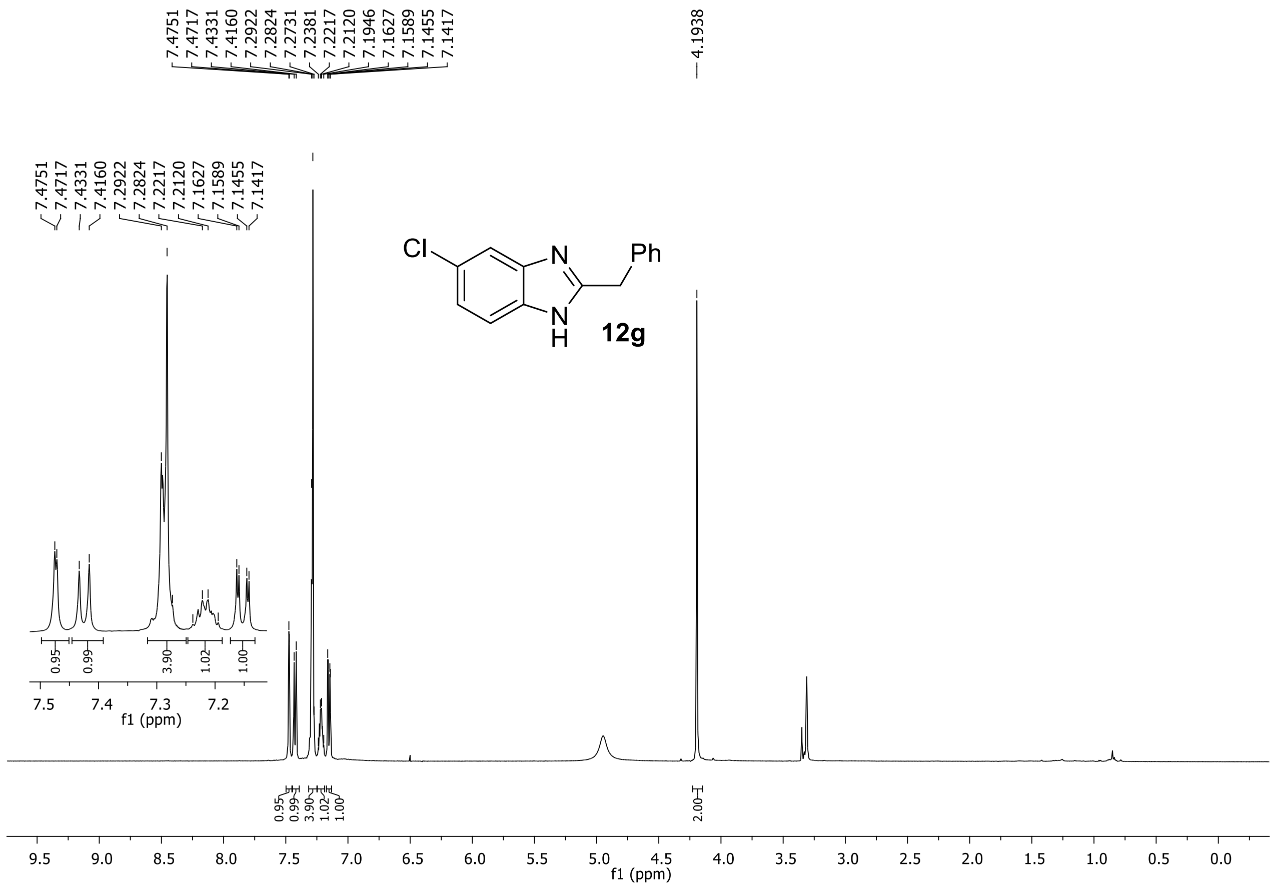

Figure S53. ^{1}H NMR spectrum of compound **12g** (500 MHz, $(CD_3)_2CO$).

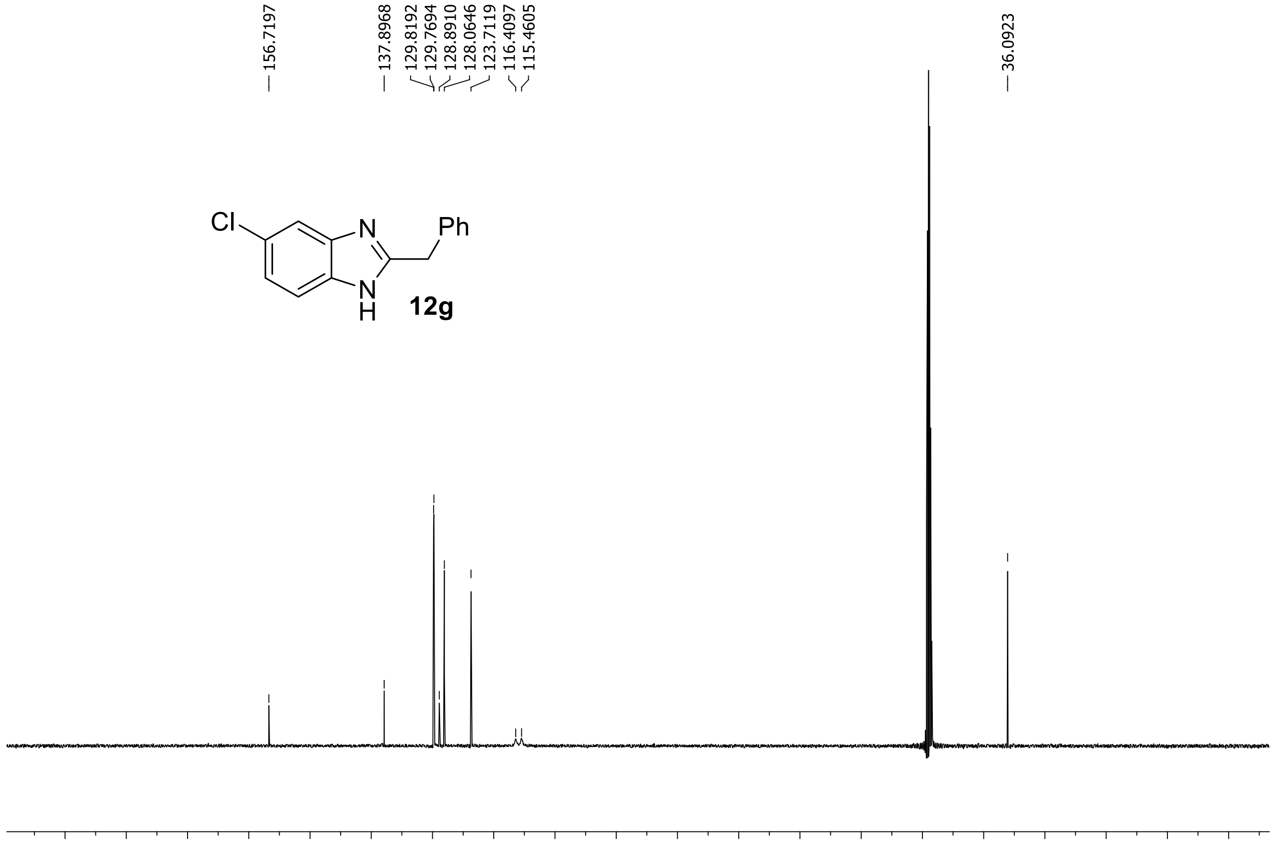

Figure S54. ^{13}C NMR spectrum of compound **12g** (125 MHz, $(CD_3)_2CO$).

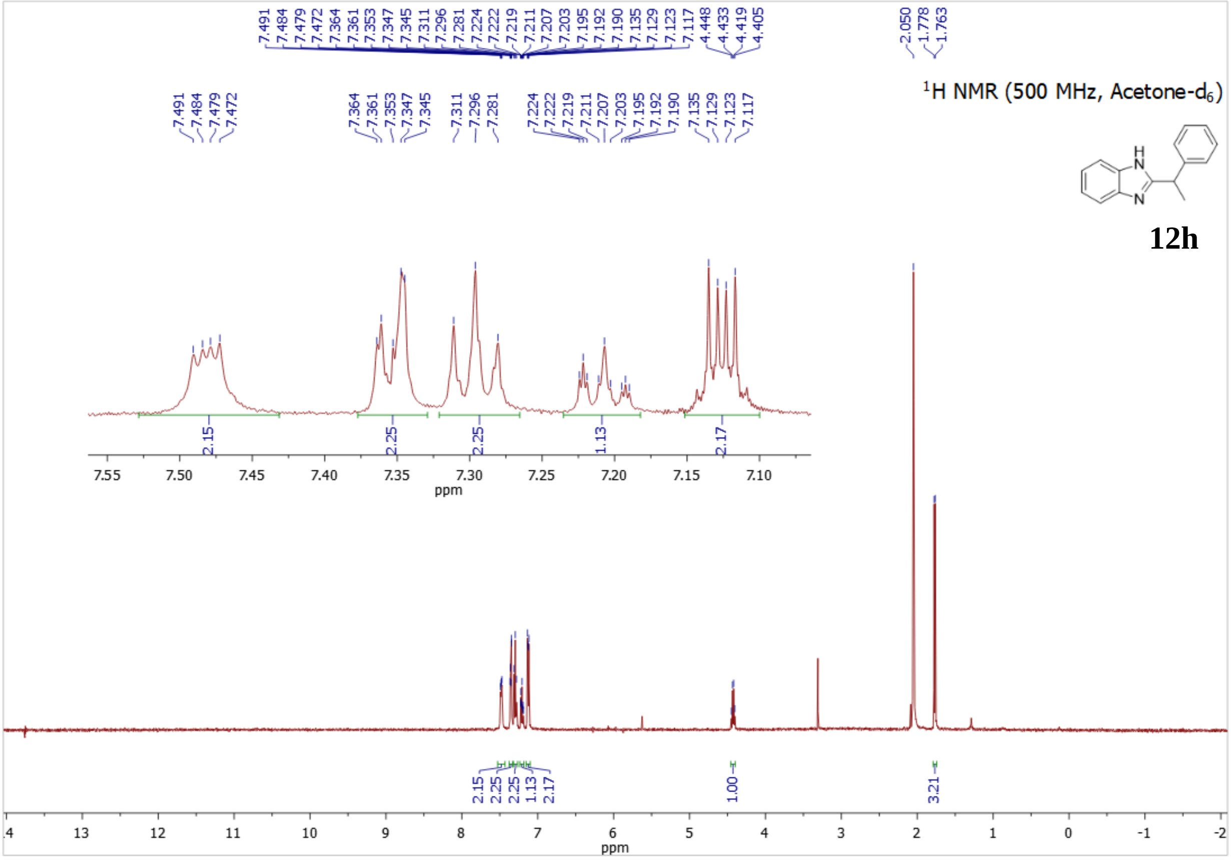

Figure S55. ^{1}H NMR spectrum of compound **12h** (500 MHz, $(CD_3)_2CO$).

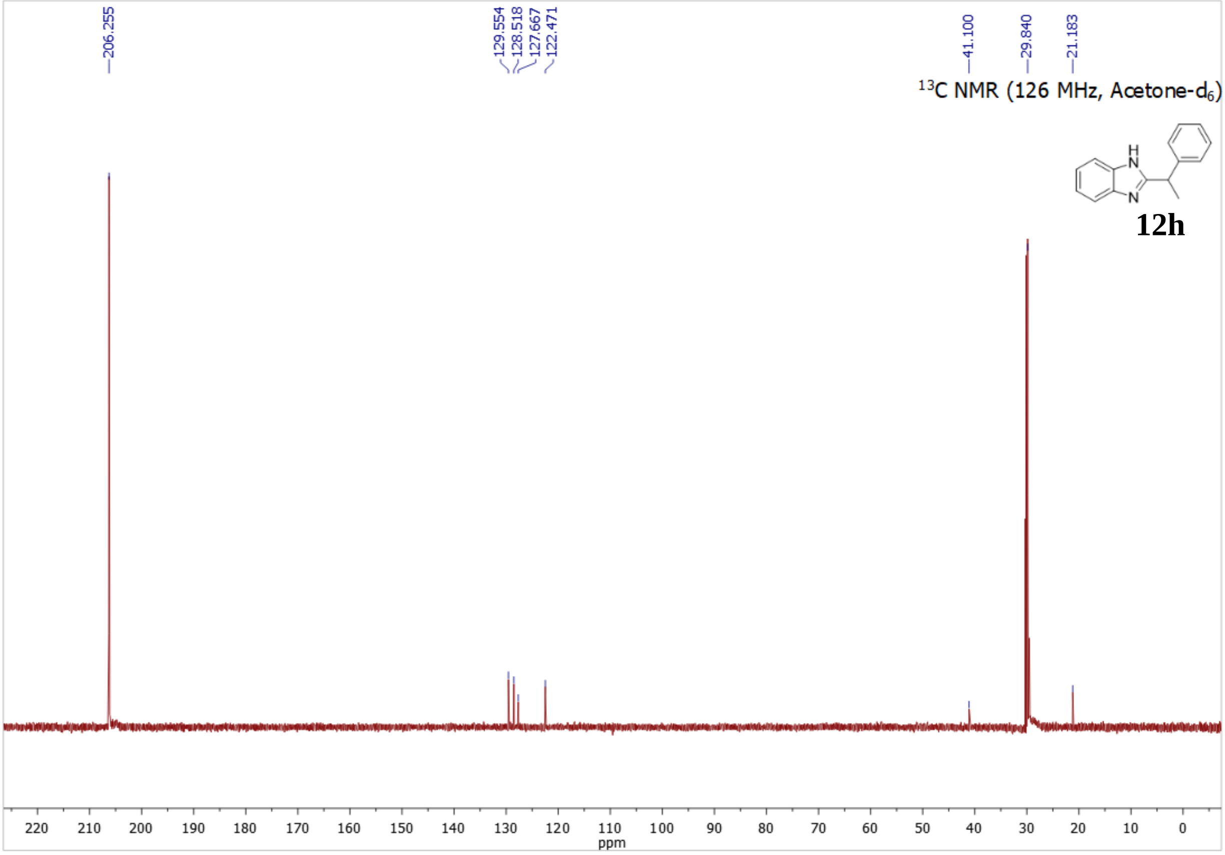

Figure S56. ^{13}C NMR spectrum of compound **12h** (125 MHz, $(CD_3)_2CO$).

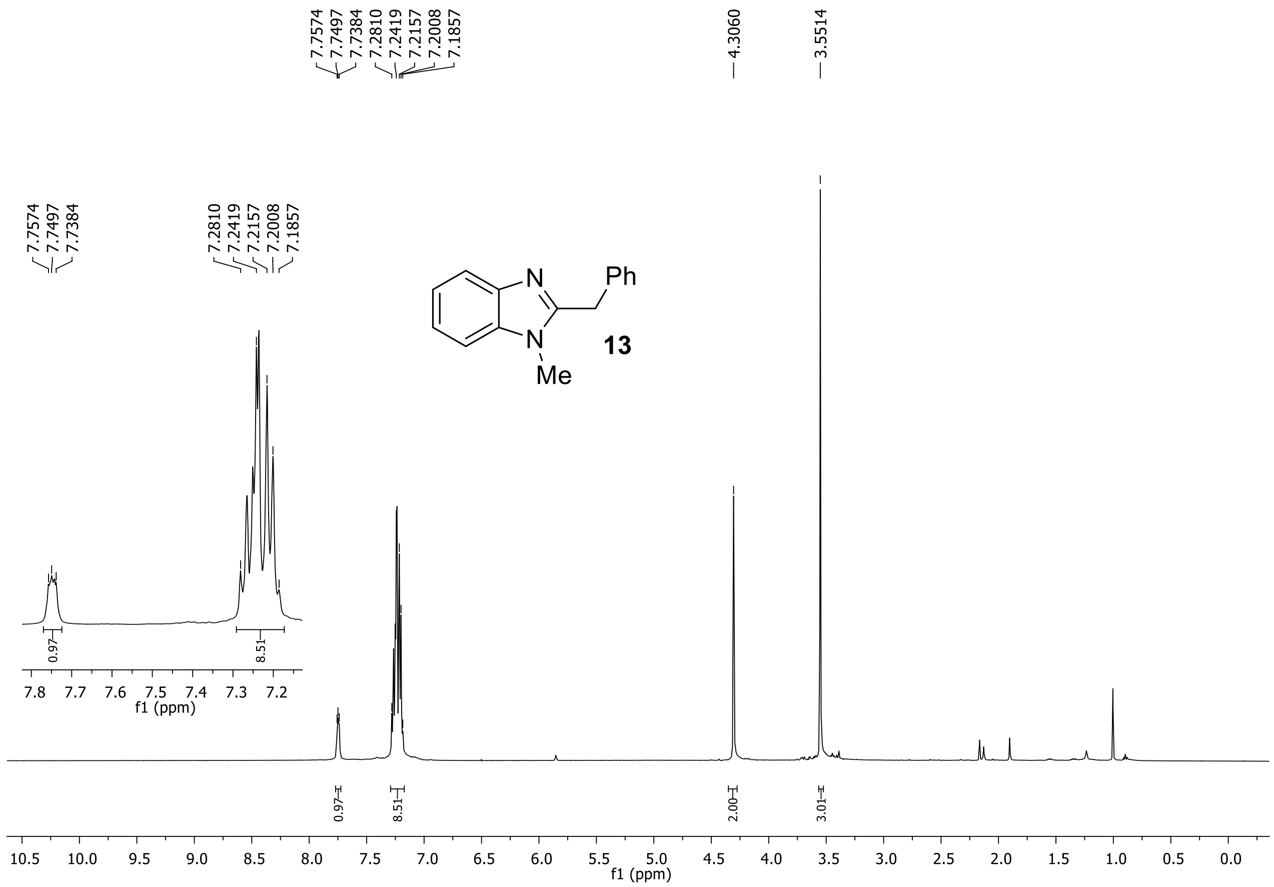

Figure S57. ^{1}H NMR spectrum of compound **13** (500 MHz, $CDCl_3$).

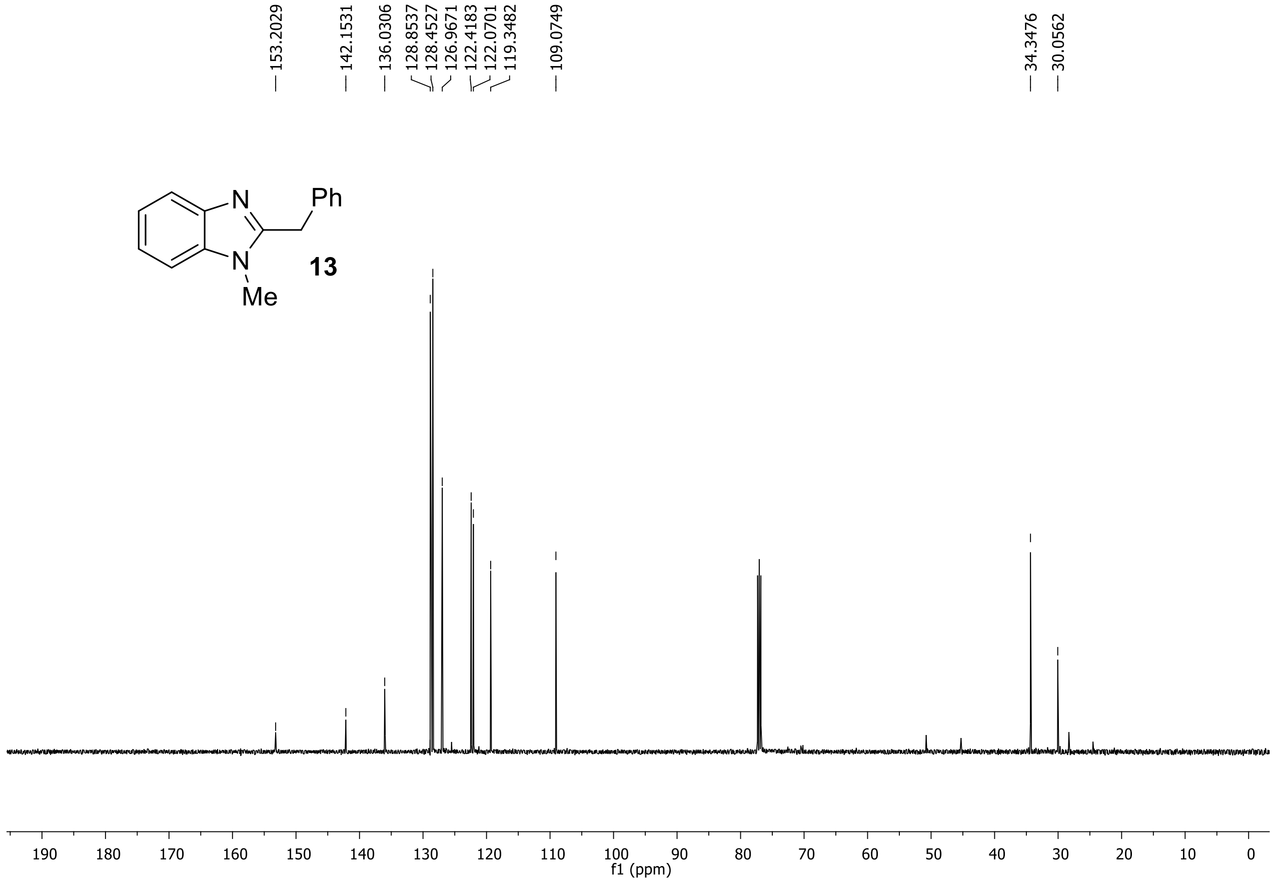

Figure S58. ^{13}C NMR spectrum of compound **13** (125 MHz, $CDCl_3$).

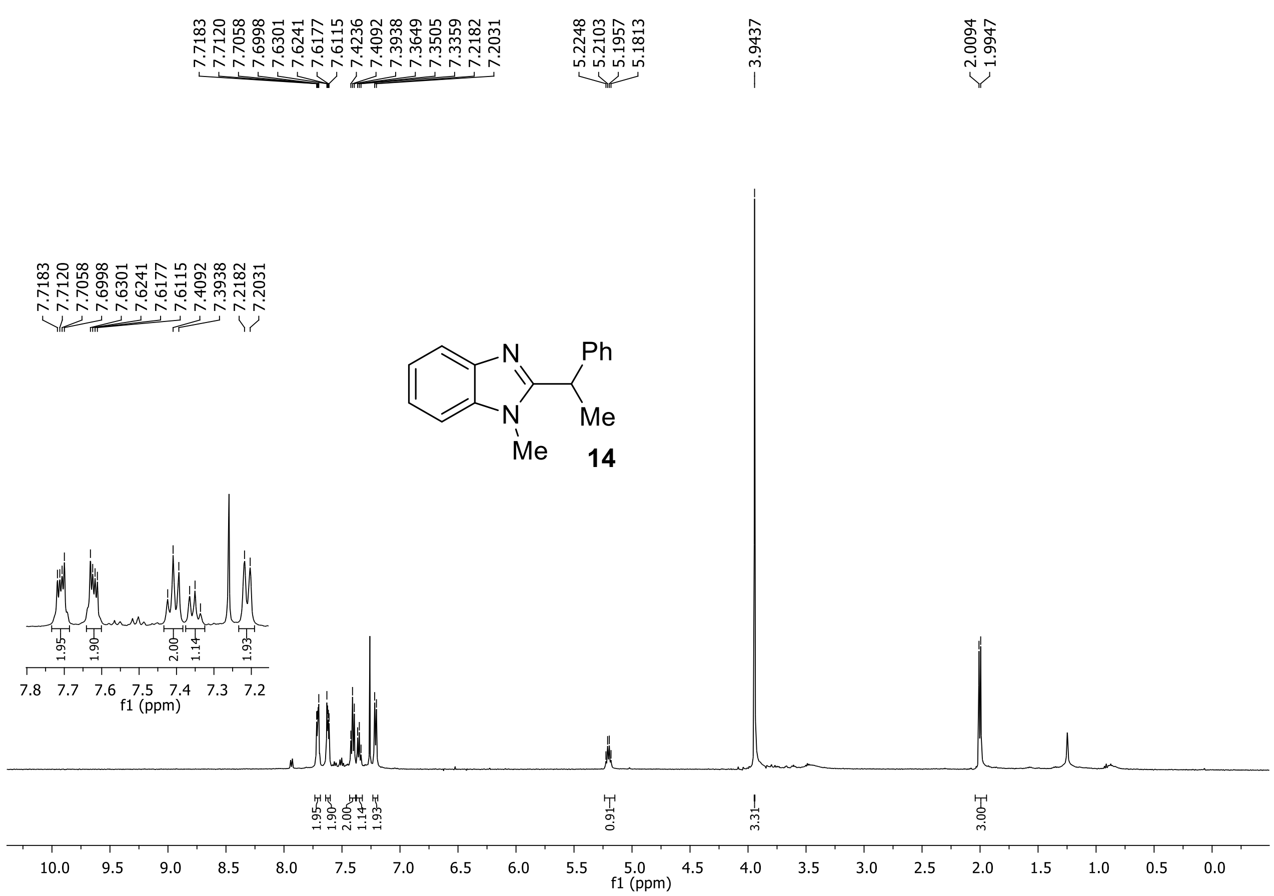

Figure S59. ^{1}H NMR spectrum of compound **14** (500 MHz, $CDCl_3$).

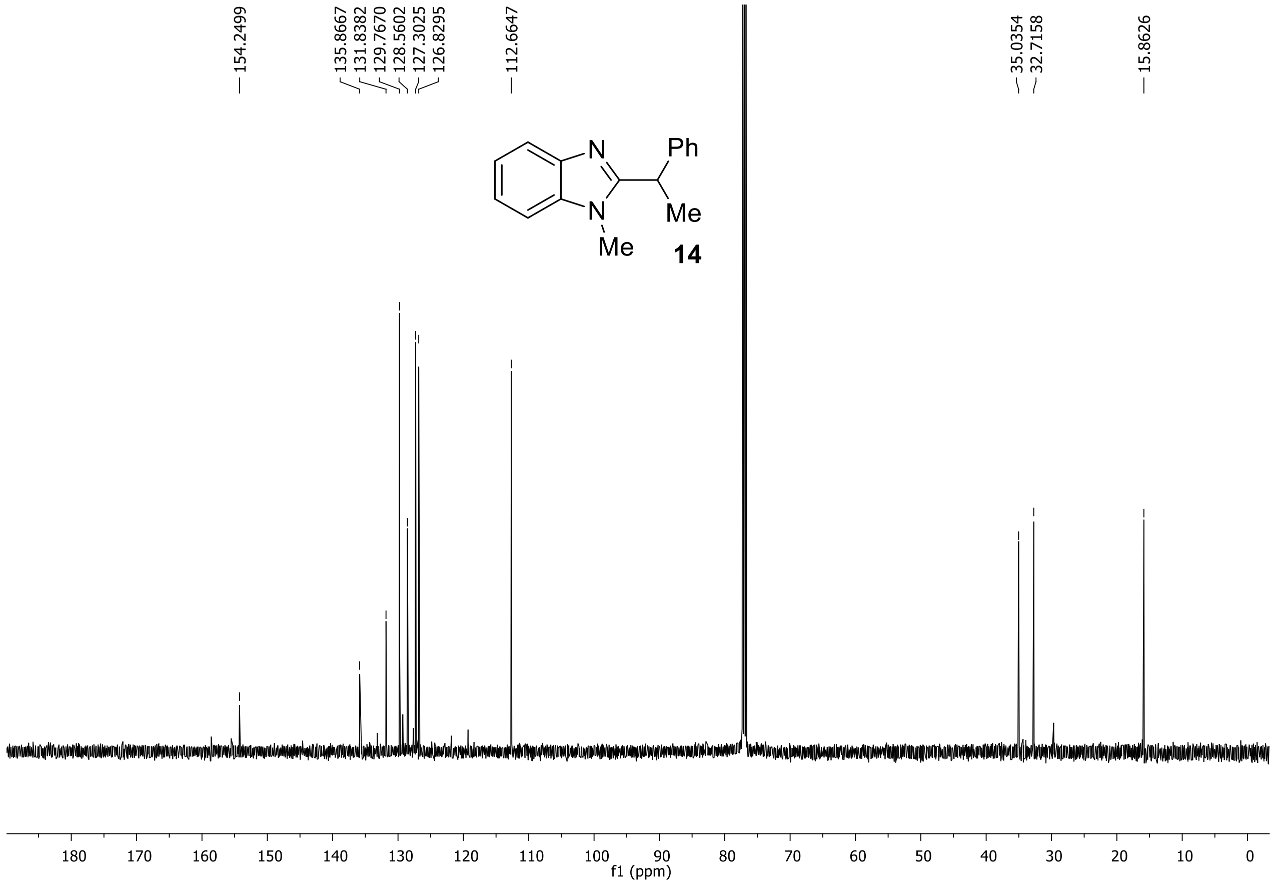

Figure S60. ^{13}C NMR spectrum of compound **14** (125 MHz, $CDCl_3$).

4. ^{1}H, ^{15}N and ^{13}C NMR spectra of cations I, III, VIII in TfOH.

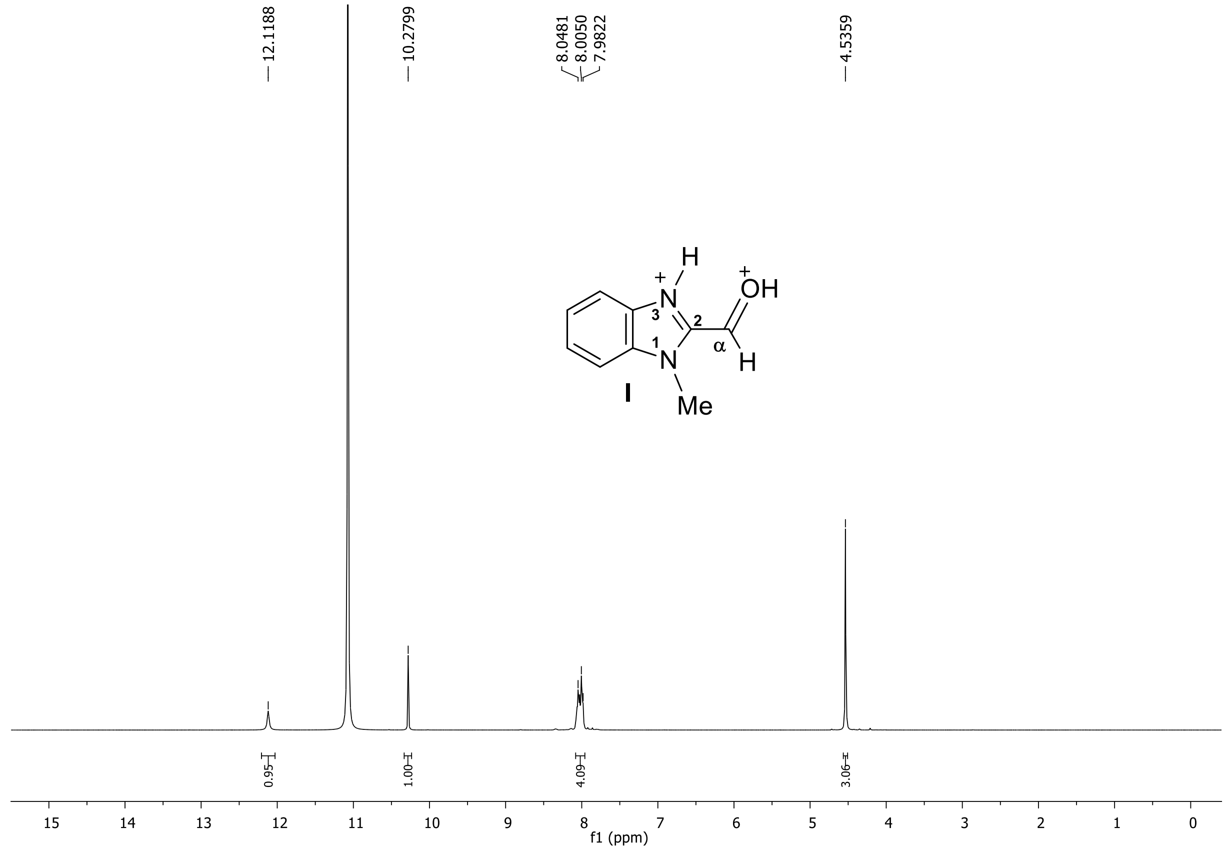

Figure S61. ^{1}H NMR spectrum of cations **I** (400 MHz, TfOH).

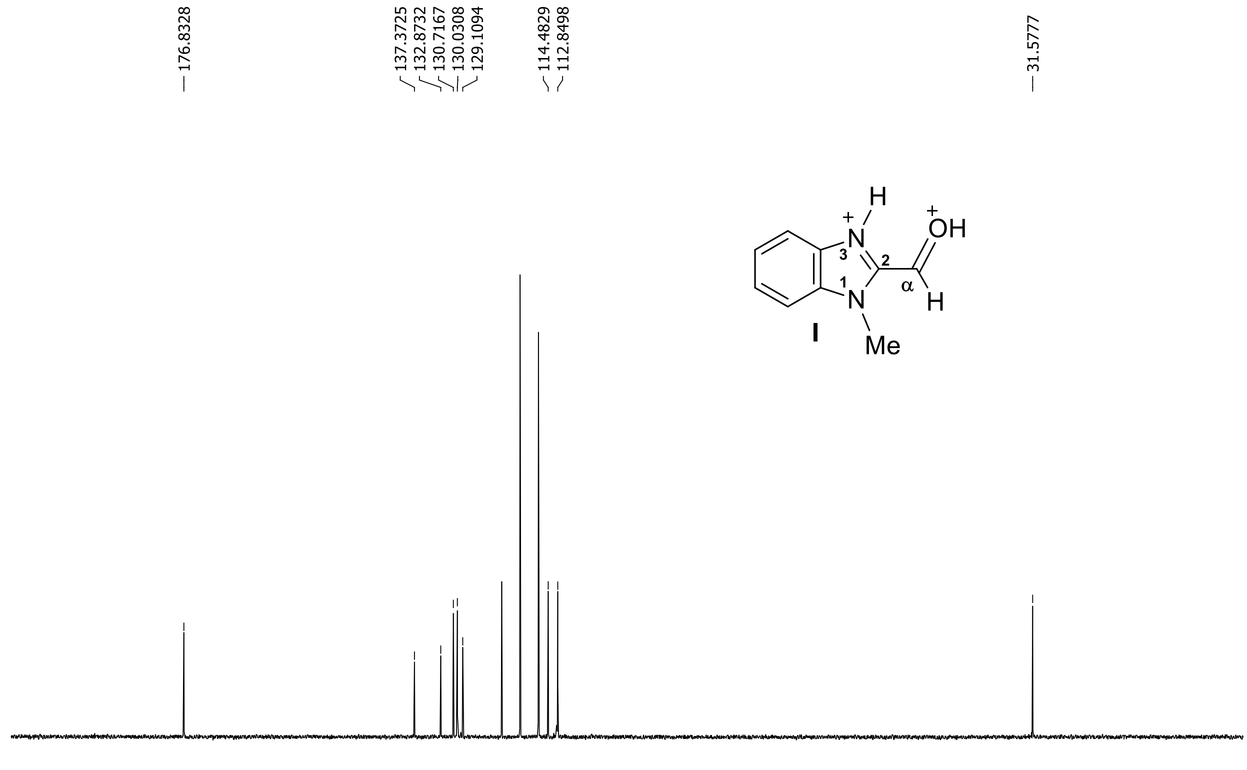

Figure S62. ^{13}C NMR spectrum of of cations **I** (100 MHz, TfOH).

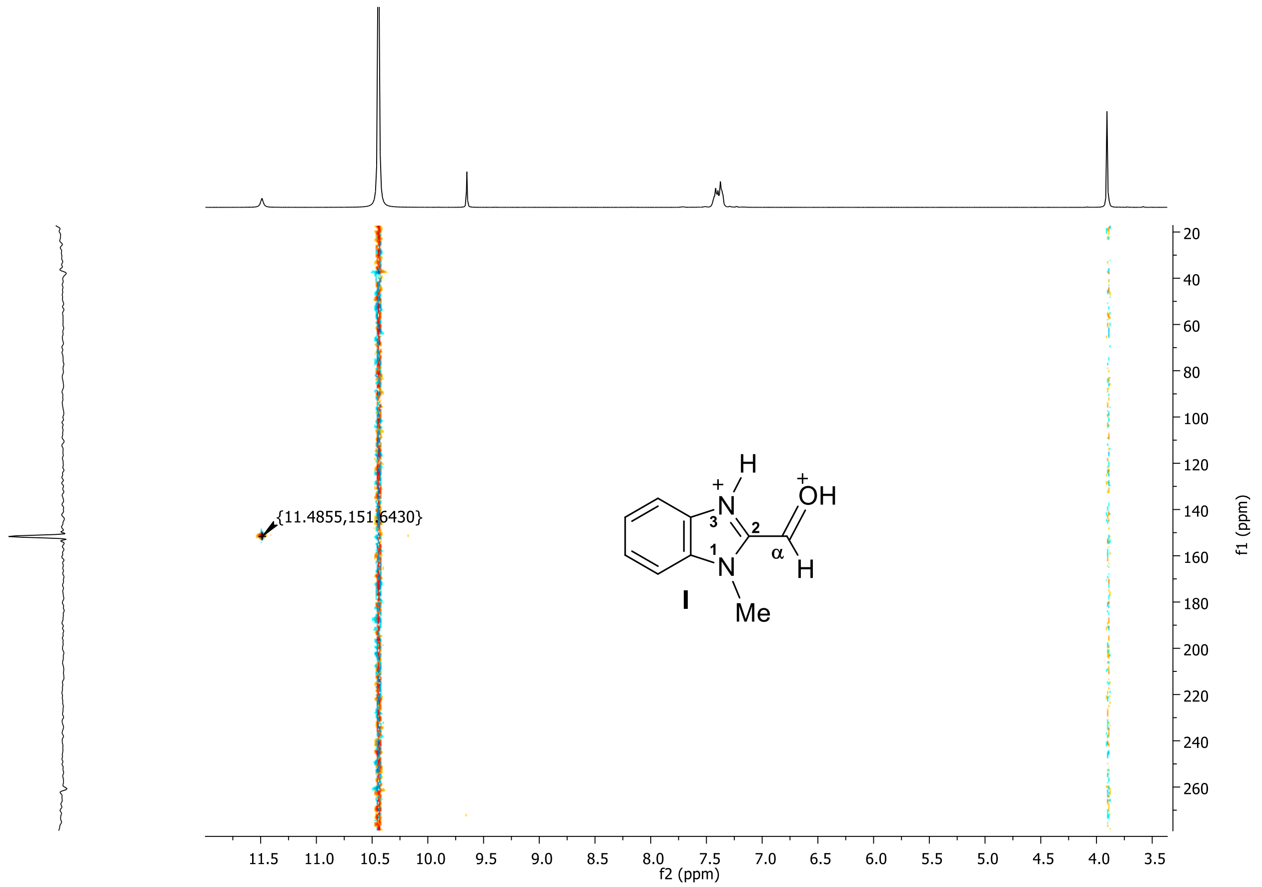

Figure S63. HSQC ^{1}H-^{15}N NMR spectrum of cations **I** (400 MHz, TfOH).

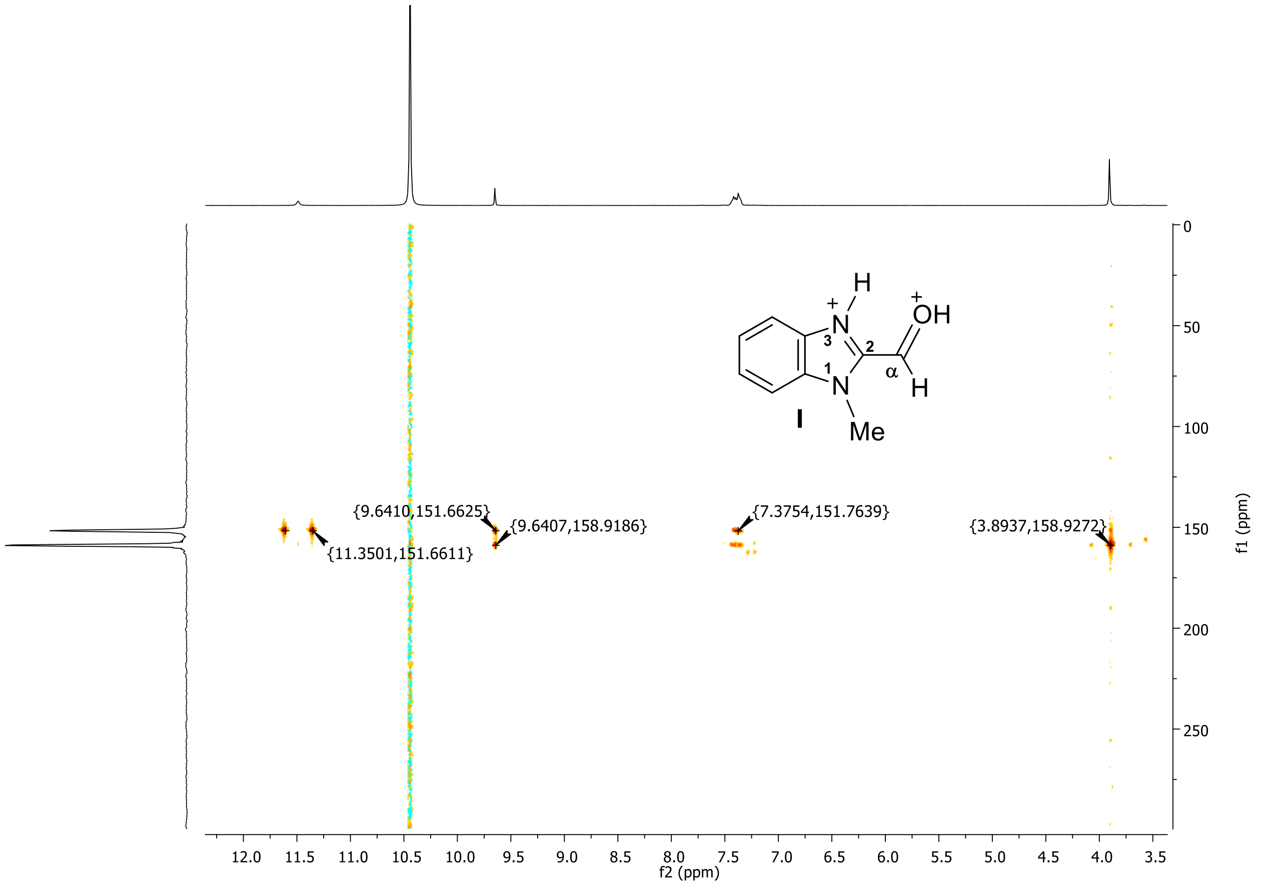

Figure S64. HMBC ^{1}H-^{15}N NMR spectrum of cations **I** (400 MHz, TfOH).

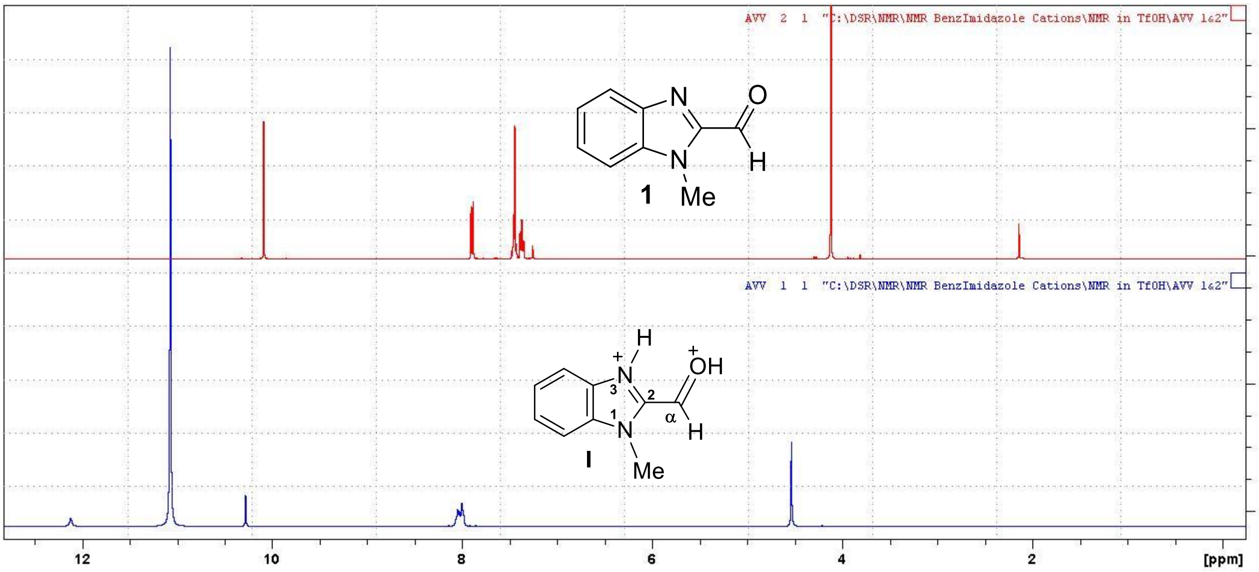

Figure S65. ^{1}H NMR spectrums of cations **I** (400 MHz, TfOH) and neutral initial compounds **1** (400 MHz, $CDCl_3$).

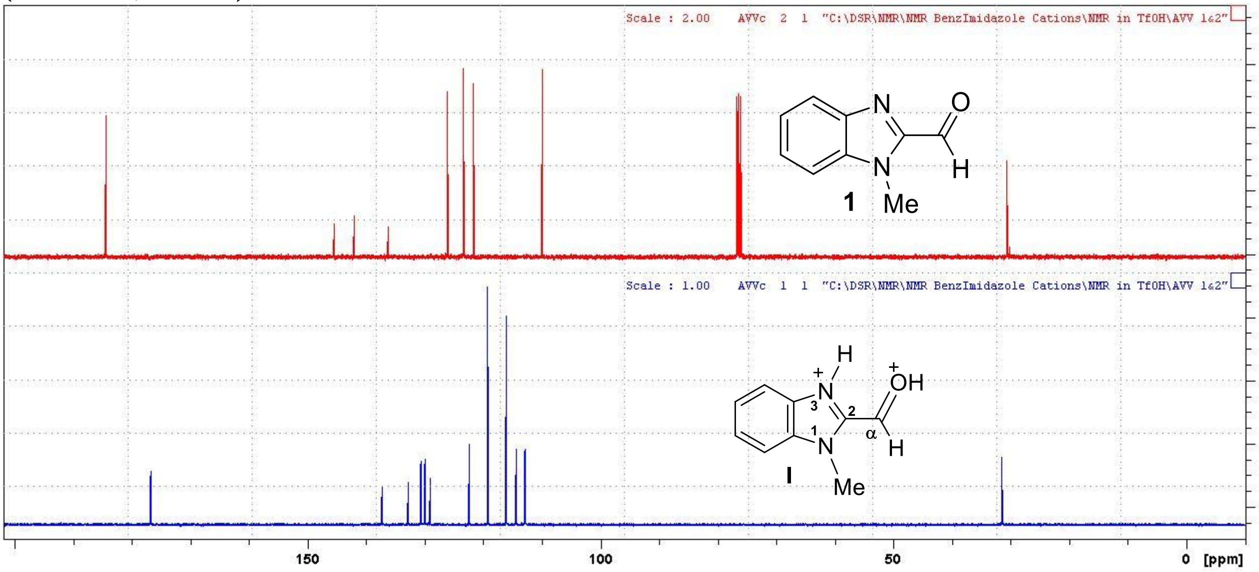

Figure S66. ^{1}H NMR spectrums of cations **I** (400 MHz, TfOH) and neutral initial compounds **1** (400 MHz, $CDCl_3$).

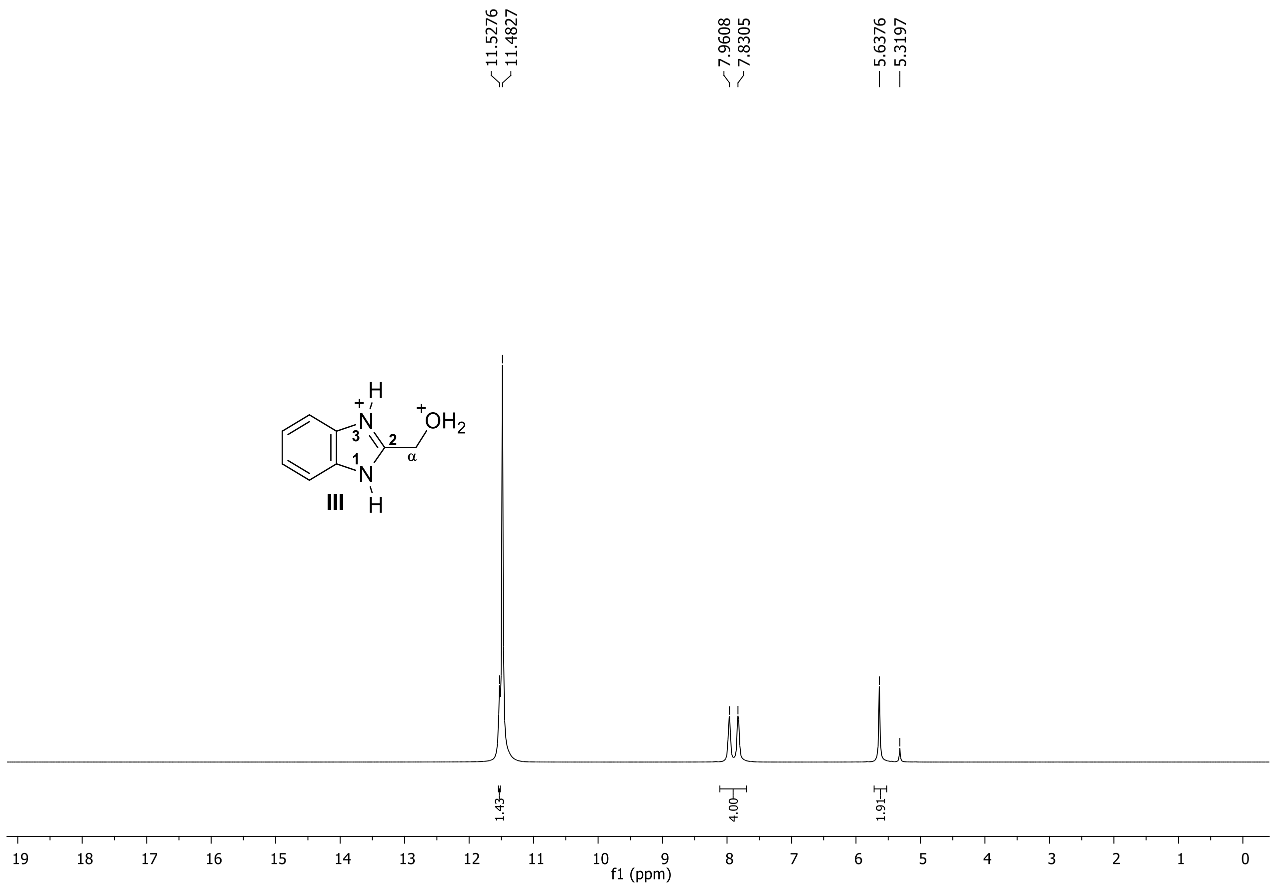

Figure S67. ^{1}H NMR spectrum of cations **III** (400 MHz, TfOH).

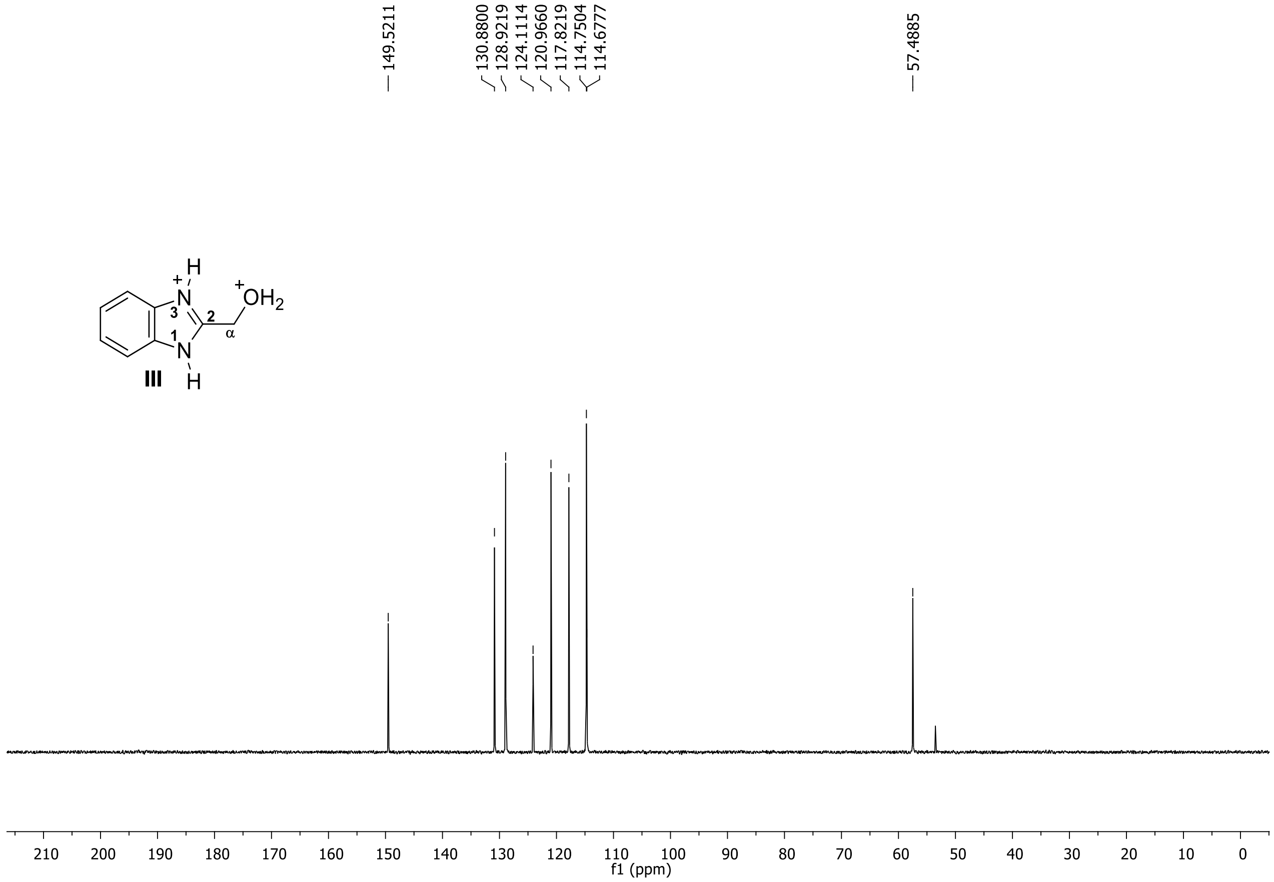

Figure S68. ^{13}C NMR spectrum of of cations **III** (100 MHz, TfOH).

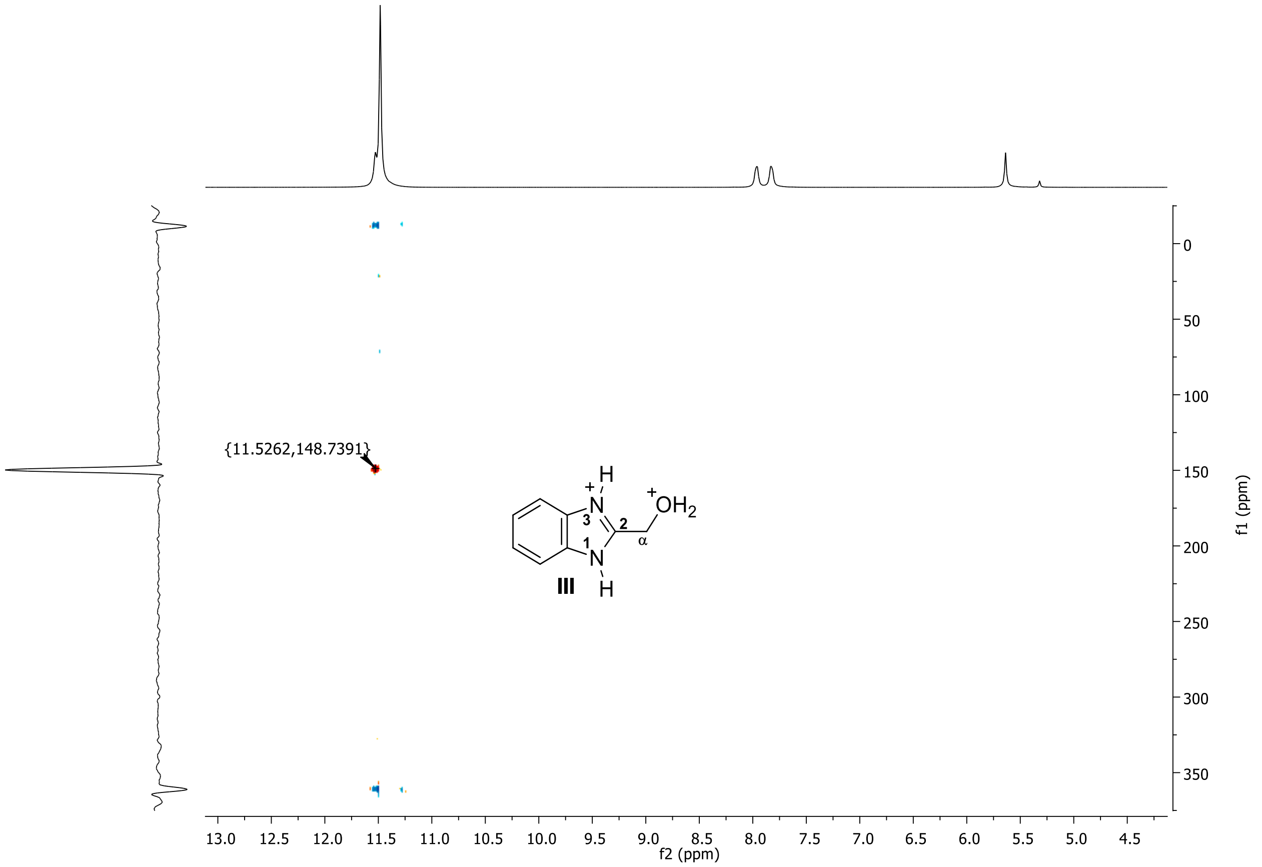

Figure S69. HSQC ^{1}H-^{15}N NMR spectrum of cations **III** (400 MHz, TfOH).

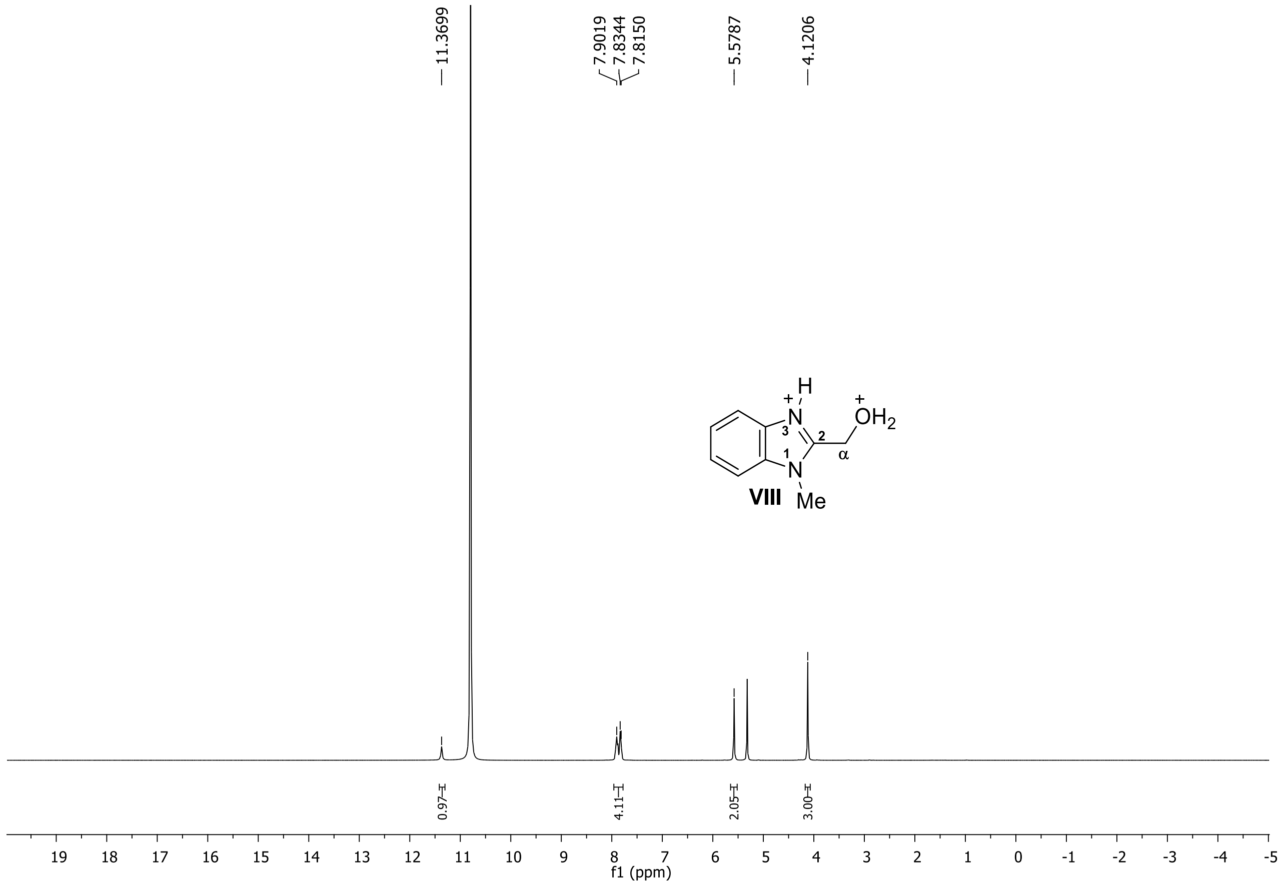

Figure S70. ^{1}H NMR spectrum of cations **VIII** (400 MHz, TfOH).

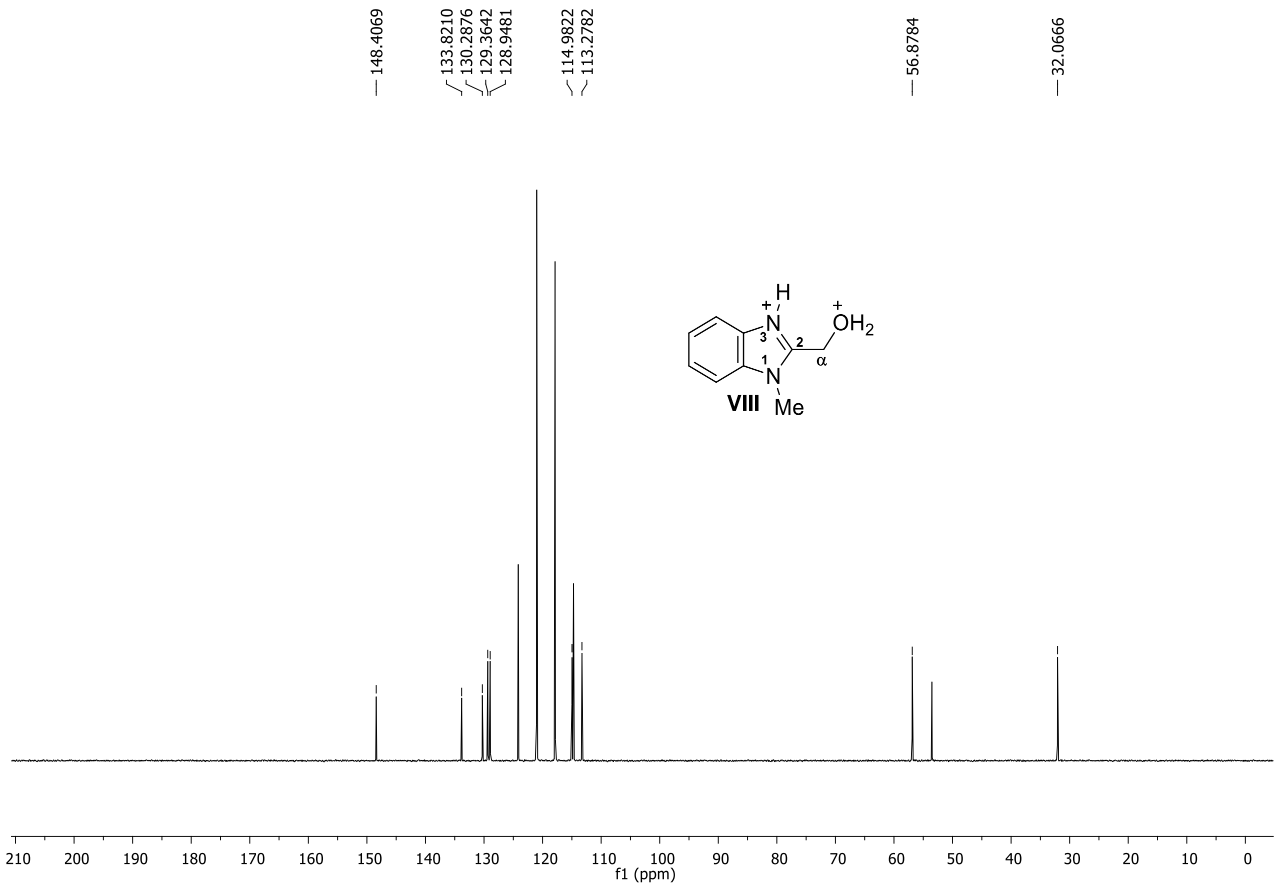

Figure S71. ^{13}C NMR spectrum of of cations **VIII** (100 MHz, TfOH).

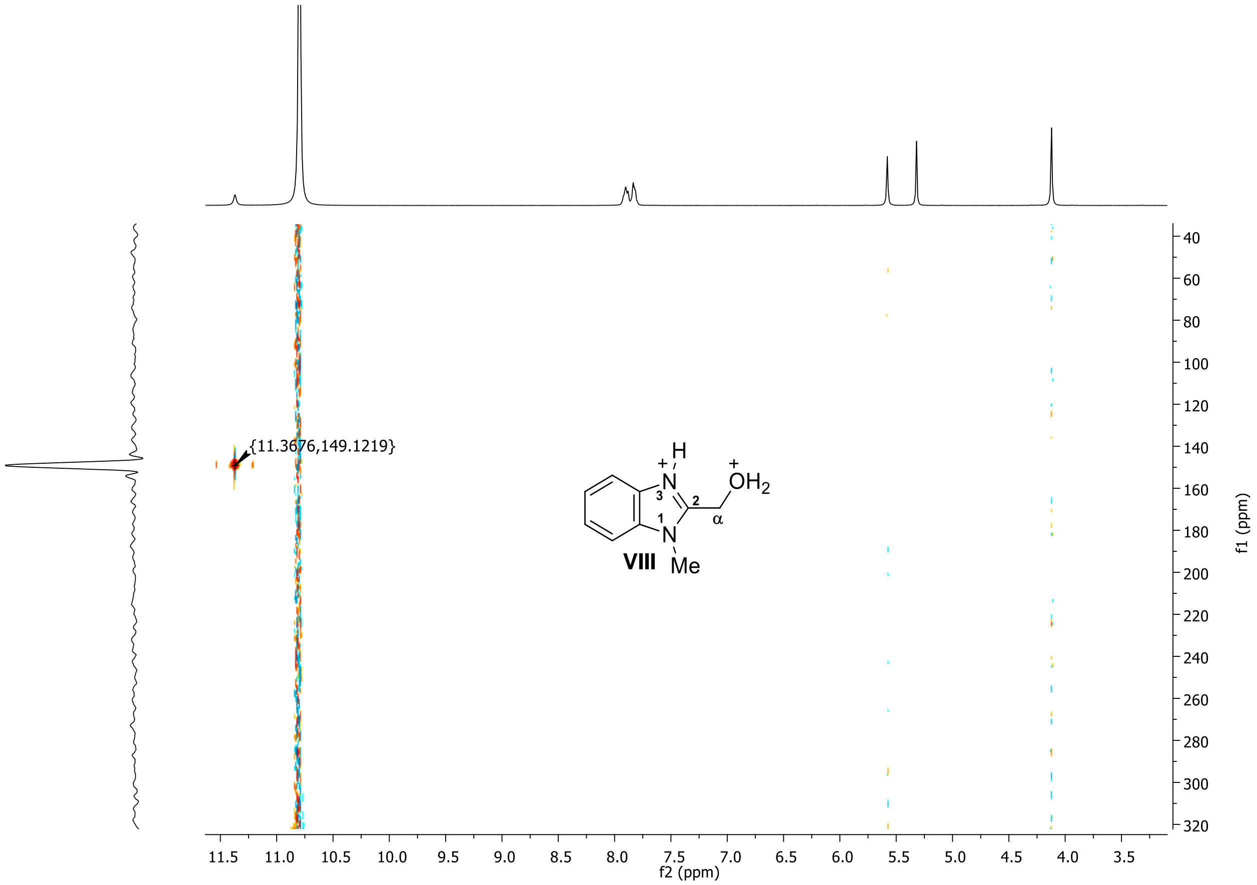

Figure S72. HSQC ^{1}H-^{15}N NMR spectrum of cations **VIII** (400 MHz, TfOH).

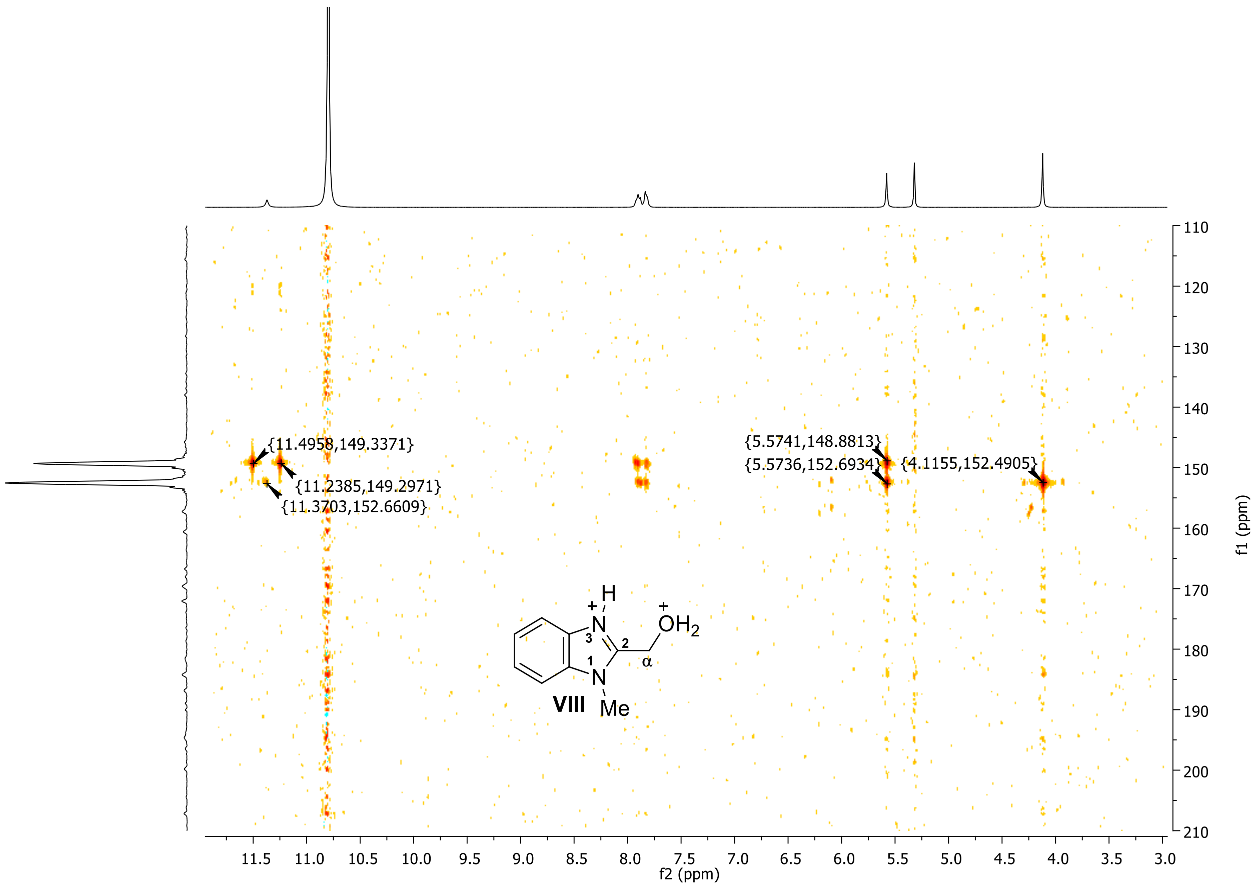

Figure S73. HMBC ^{1}H-^{15}N NMR spectrum of cations **VIII** (400 MHz, TfOH).

5. Details of DFT-calculations of cations I–IX

I q=0

Energy **E= -494.369025175 h, G^{298}= -494.250859 h, μ=4.6 D**

Cartesian coordinates, Å

N	atom	x	y	z
1	C	-1.958250	-1.339476	-0.000028
2	C	-0.681328	-0.776506	0.000018
3	C	-0.531873	0.621836	0.000070
4	C	-1.618320	1.491038	0.000051
5	C	-2.876726	0.913415	-0.000050
6	C	-3.044340	-0.481707	-0.000088
7	C	1.419549	-0.398245	0.000068
8	N	0.830898	0.827314	0.000114
9	H	-2.087499	-2.412924	-0.000072
10	H	-1.486799	2.563543	0.000108
11	H	-3.750540	1.549831	-0.000103
12	H	-4.045399	-0.889595	-0.000154
13	H	1.330352	1.701637	0.000357
14	N	0.565012	-1.387048	0.000015
15	C	2.907023	-0.544551	0.000102
16	H	3.204894	-1.110633	0.885102
17	H	3.204778	-1.111181	-0.884595
18	O	3.467522	0.754168	-0.000306
19	H	4.424270	0.679284	0.000045

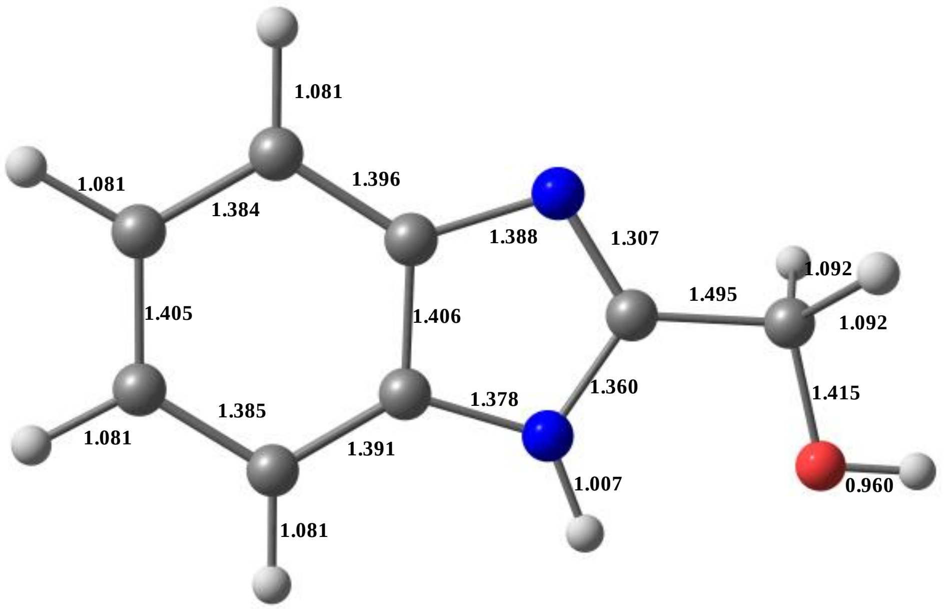

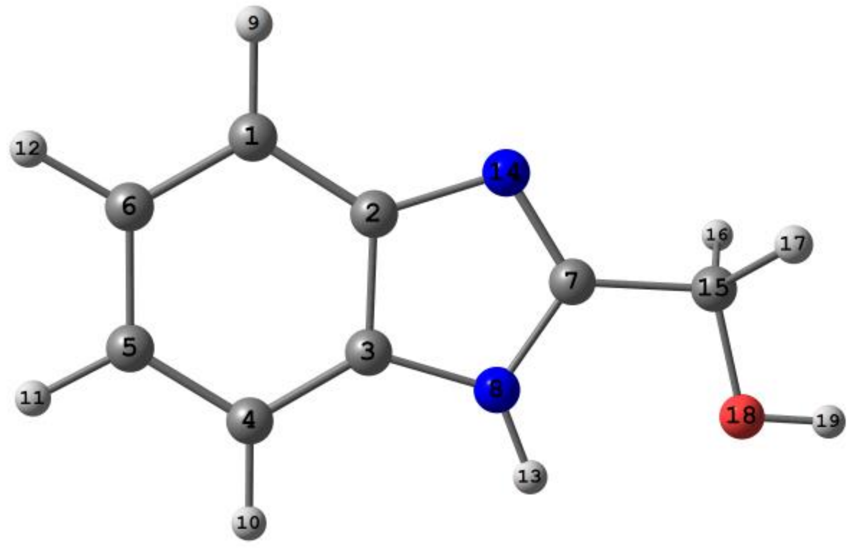

Summary of Natural Population Analysis:

Atom	No	Natural Charge	Core	Valence	Rydberg	Total
			Natural Population			
C	1	-0.21368	1.99902	4.19479	0.01987	6.21368
C	2	0.11387	1.99888	3.86176	0.02549	5.88613
C	3	0.12704	1.99890	3.85388	0.02017	5.87296
C	4	-0.23088	1.99903	4.21323	0.01862	6.23088
C	5	-0.21665	1.99916	4.19750	0.01998	6.21665
C	6	-0.22671	1.99916	4.20741	0.02014	6.22671
C	7	0.44094	1.99924	3.53022	0.02961	5.55906
N	8	-0.56754	1.99920	5.55162	0.01672	7.56754
H	9	0.21538	0.00000	0.78242	0.00220	0.78462
H	10	0.22017	0.00000	0.77786	0.00197	0.77983
H	11	0.21013	0.00000	0.78827	0.00160	0.78987
H	12	0.20931	0.00000	0.78907	0.00161	0.79069
H	13	0.44682	0.00000	0.55006	0.00312	0.55318
N	14	-0.58403	1.99934	5.54665	0.03804	7.58403
C	15	-0.05263	1.99909	4.03147	0.02207	6.05263
H	16	0.19303	0.00000	0.80453	0.00243	0.80697
H	17	0.19302	0.00000	0.80454	0.00243	0.80698
O	18	-0.77736	1.99978	6.76571	0.01187	8.77736
H	19	0.49977	0.00000	0.49575	0.00448	0.50023
* Total *		0.00000	21.99080	55.74676	0.26244	78.00000

I q=2

Energy **E= -495.183537902 h, G^{298}= -495.036881 h, μ=18.8 D**

Cartesian coordinates, Å

N	atom	x	y	z
1	C	1.825647	1.454948	-0.017676
2	C	0.671538	0.684549	0.104551
3	C	0.707708	-0.711133	0.073487
4	C	1.900445	-1.414630	-0.083083
5	C	3.039736	-0.651432	-0.207366
6	C	3.003136	0.758335	-0.174231
7	C	-1.393834	-0.071609	0.339654
8	N	-0.601180	-1.131603	0.222848
9	H	1.791176	2.533051	0.009506
10	H	1.923391	-2.493274	-0.107824
11	H	3.992160	-1.144660	-0.334656
12	H	3.928995	1.305217	-0.275071
13	H	-0.917699	-2.093116	0.241177
14	N	-0.655960	1.031539	0.275049
15	C	-2.868296	-0.115574	0.509080
16	H	-3.218302	-1.086731	0.840082
17	H	-3.216943	0.672245	1.167605
18	O	-3.487505	0.183489	-0.808531
19	H	-4.396514	0.533099	-0.731289
20	H	-3.483267	-0.567699	-1.433674
21	H	-1.019461	1.973681	0.340625

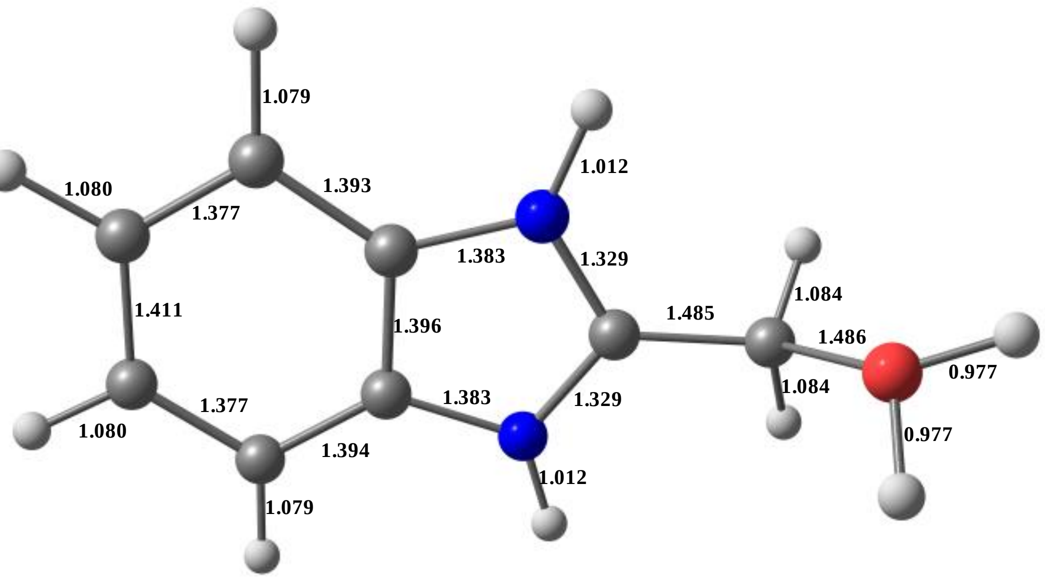

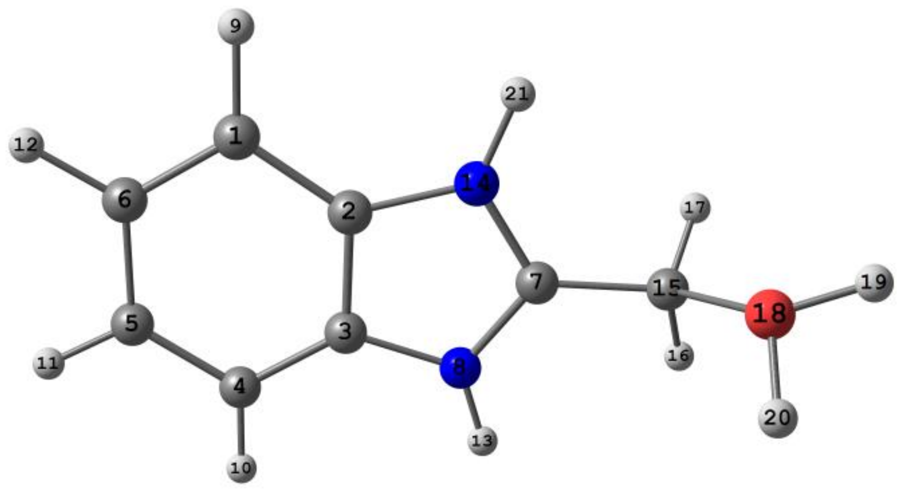

Summary of Natural Population Analysis:

Atom	No	Natural Charge	Natural Population: Core	Valence	Rydberg	Total
C	1	-0.19950	1.99902	4.18238	0.01810	6.19950
C	2	0.15815	1.99890	3.82308	0.01987	5.84185
C	3	0.15473	1.99890	3.82639	0.01998	5.84527
C	4	-0.19796	1.99902	4.18088	0.01805	6.19796
C	5	-0.16938	1.99916	4.15128	0.01894	6.16938
C	6	-0.16766	1.99916	4.14958	0.01892	6.16766
C	7	0.46045	1.99926	3.51624	0.02404	5.53955
N	8	-0.47236	1.99918	5.45761	0.01556	7.47236
H	9	0.24228	0.00000	0.75599	0.00173	0.75772
H	10	0.24235	0.00000	0.75593	0.00173	0.75765
H	11	0.22744	0.00000	0.77109	0.00146	0.77256
H	12	0.22745	0.00000	0.77108	0.00146	0.77255
H	13	0.47681	0.00000	0.52076	0.00243	0.52319
N	14	-0.47602	1.99918	5.46103	0.01580	7.47602
C	15	-0.05970	1.99901	4.04144	0.01924	6.05970
H	16	0.26203	0.00000	0.73646	0.00151	0.73797
H	17	0.26943	0.00000	0.72892	0.00164	0.73057
O	18	-0.63686	1.99972	6.62783	0.00931	8.63686
H	19	0.59256	0.00000	0.40464	0.00280	0.40744
H	20	0.58930	0.00000	0.40784	0.00286	0.41070
H	21	0.47645	0.00000	0.52110	0.00245	0.52355
* Total *		2.00000	21.99053	55.79156	0.21791	78.00000

II q=2

Energy **E= -418.701138027 h, G^{298}= -418.584651 h, μ=7.3 D**

Cartesian coordinates, Å

N	atom	x	y	z
1	C	1.355268	1.469278	0.000036
2	C	0.151153	0.731472	0.000005
3	C	0.151170	-0.731480	0.000034
4	C	1.355269	-1.469268	-0.000002
5	C	2.488444	-0.727421	-0.000008
6	C	2.488437	0.727432	0.000009
7	C	-1.946900	-0.000012	-0.000046
8	N	-1.111479	-1.103515	-0.000015
9	H	1.352524	2.547545	0.000026
10	H	1.352513	-2.547535	-0.000027
11	H	3.447755	-1.224138	-0.000046
12	H	3.447744	1.224156	-0.000006
13	H	-1.450666	-2.062259	-0.000006
14	N	-1.111510	1.103500	-0.000039
15	C	-3.288768	0.000000	0.000033
16	H	-3.831398	-0.934475	0.000141
17	H	-3.831294	0.934547	0.000012
18	H	-1.450688	2.062254	-0.000086

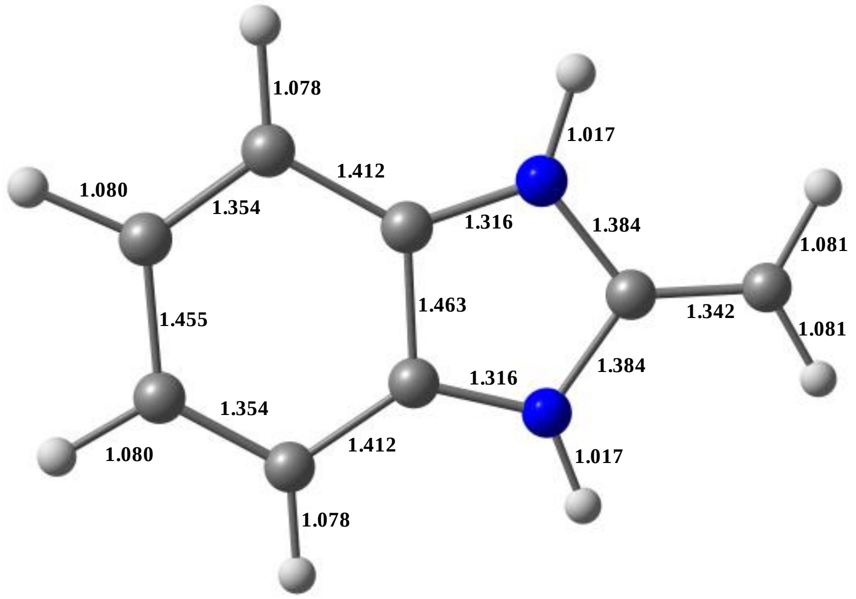

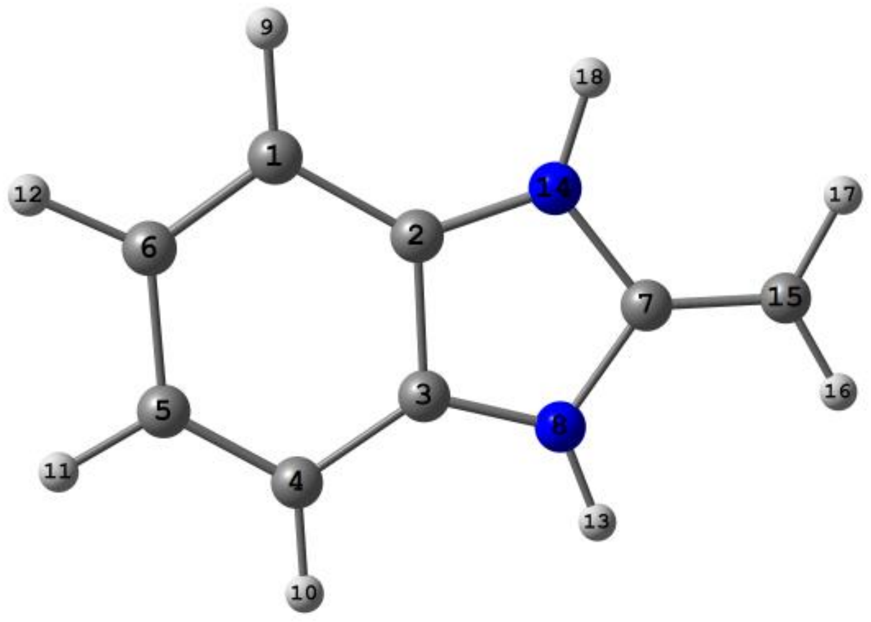

Summary of Natural Population Analysis:

Atom	No	Natural Charge	Natural Population Core	Valence	Rydberg	Total
C	1	-0.22355	1.99901	4.20554	0.01900	6.22355
C	2	0.31791	1.99908	3.66215	0.02086	5.68209
C	3	0.31790	1.99908	3.66216	0.02086	5.68210
C	4	-0.22356	1.99901	4.20555	0.01900	6.22356
C	5	-0.05983	1.99915	4.04228	0.01840	6.05983
C	6	-0.05985	1.99915	4.04230	0.01840	6.05985
C	7	0.31324	1.99919	3.66570	0.02187	5.68676
N	8	-0.42428	1.99916	5.40868	0.01645	7.42428
H	9	0.26656	0.00000	0.73179	0.00166	0.73344
H	10	0.26656	0.00000	0.73179	0.00166	0.73344
H	11	0.24729	0.00000	0.75137	0.00135	0.75271
H	12	0.24729	0.00000	0.75137	0.00135	0.75271
H	13	0.49449	0.00000	0.50311	0.00239	0.50551
N	14	-0.42429	1.99916	5.40868	0.01645	7.42429
C	15	-0.06389	1.99910	4.05280	0.01199	6.06389
H	16	0.25677	0.00000	0.74198	0.00125	0.74323
H	17	0.25676	0.00000	0.74198	0.00125	0.74324
H	18	0.49450	0.00000	0.50311	0.00239	0.50550
* Total *		2.00000	19.99108	47.81233	0.19658	68.00000

III q=0

Energy **E= -533.66804816 h, G^{298}= -533.523079 h, μ=5.6 D**

Cartesian coordinates, Å

N	atom	x	y	z
1	C	-2.119462	-1.457640	-0.035984
2	C	-0.838698	-0.910902	0.060796
3	C	-0.669609	0.483778	0.047751
4	C	-1.738993	1.369605	-0.059872
5	C	-3.000855	0.807893	-0.153016
6	C	-3.188517	-0.585649	-0.142323
7	C	1.261281	-0.548340	0.228043
8	N	0.685842	0.689206	0.161574
9	H	-2.264470	-2.529096	-0.028059
10	H	-1.593814	2.440782	-0.070223
11	H	-3.862666	1.455169	-0.235878
12	H	-4.191969	-0.980084	-0.219658
13	N	0.394478	-1.530051	0.171987
14	C	2.736028	-0.733890	0.355648
15	H	3.099085	-0.223493	1.251908
16	H	2.924205	-1.801581	0.462218
17	O	3.355538	-0.204587	-0.809078
18	H	4.307914	-0.234962	-0.685150
19	C	1.336752	1.985437	0.202564
20	H	0.879507	2.597646	0.977424
21	H	2.390064	1.850817	0.422837
22	H	1.238043	2.485660	-0.759366

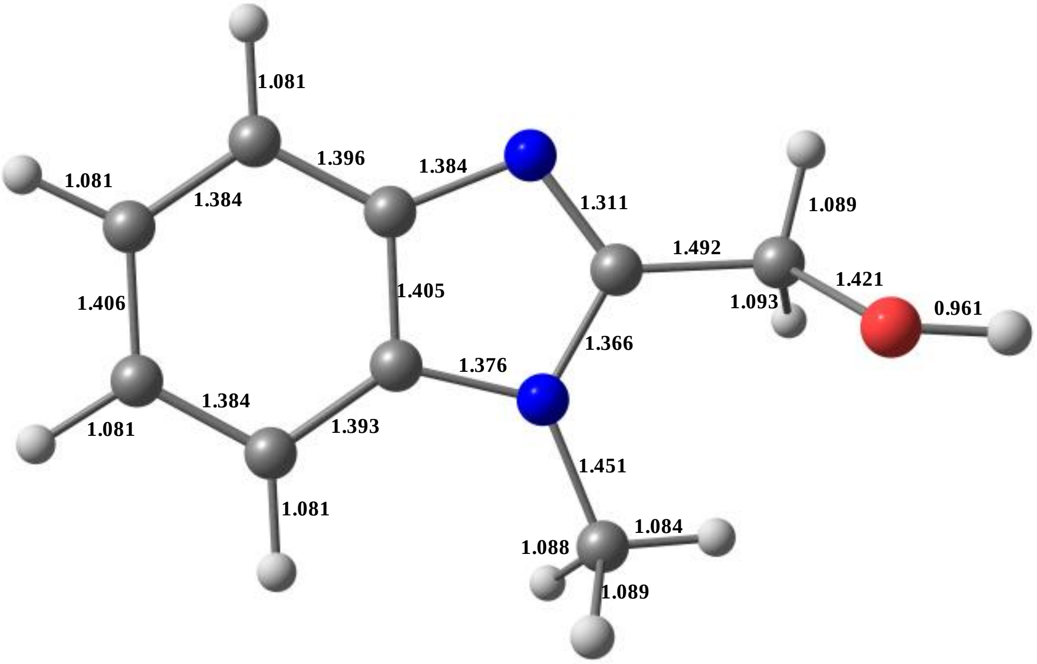

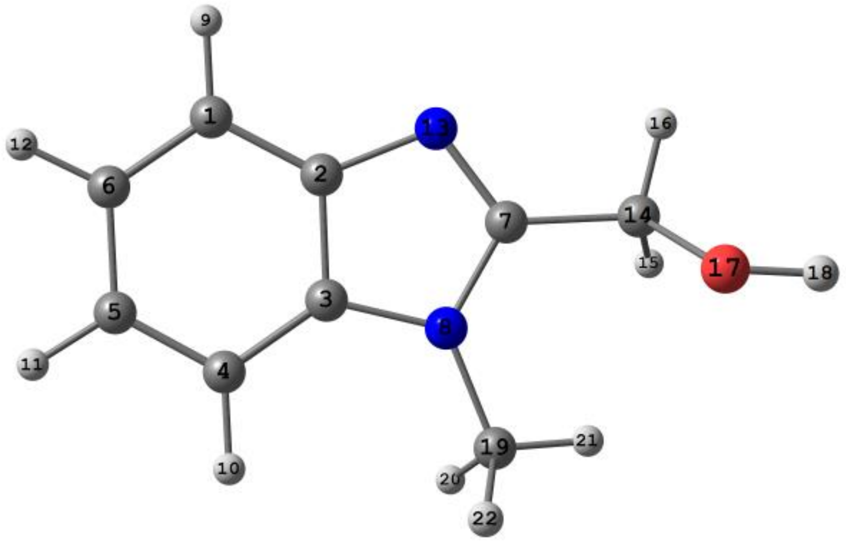

Summary of Natural Population Analysis:

Atom	No	Natural Charge	Natural Population Core	Valence	Rydberg	Total
C	1	-0.20609	1.99902	4.18757	0.01950	6.20609
C	2	0.11085	1.99888	3.86518	0.02509	5.88915
C	3	0.14671	1.99891	3.83620	0.01819	5.85329
C	4	-0.23849	1.99904	4.22166	0.01779	6.23849
C	5	-0.20929	1.99917	4.19017	0.01995	6.20929
C	6	-0.22827	1.99916	4.20895	0.02016	6.22827
C	7	0.44381	1.99918	3.52699	0.03002	5.55619
N	8	-0.43043	1.99912	5.41157	0.01975	7.43043
H	9	0.21580	0.00000	0.78208	0.00211	0.78420
H	10	0.22077	0.00000	0.77728	0.00195	0.77923
H	11	0.21051	0.00000	0.78788	0.00161	0.78949
H	12	0.20993	0.00000	0.78846	0.00161	0.79007
N	13	-0.56652	1.99936	5.52928	0.03788	7.56652
C	14	-0.06138	1.99906	4.04030	0.02202	6.06138
H	15	0.18662	0.00000	0.81150	0.00188	0.81338
H	16	0.19107	0.00000	0.80649	0.00244	0.80893
O	17	-0.77183	1.99978	6.75974	0.01231	8.77183
H	18	0.49241	0.00000	0.50290	0.00468	0.50759
C	19	-0.35649	1.99931	4.34348	0.01370	6.35649
H	20	0.20873	0.00000	0.78950	0.00177	0.79127
H	21	0.22199	0.00000	0.77646	0.00155	0.77801
H	22	0.20957	0.00000	0.78871	0.00172	0.79043
* Total *		0.00000	23.98998	61.73236	0.27766	86.00000

III q=2

Energy **E= -534.48468214 h, G^{298}= -534.312749 h, μ=18.2 D**

Cartesian coordinates, Å

N	atom	x	y	z
1	C	-2.010449	-1.586263	0.033017
2	C	-0.812582	-0.879379	0.113139
3	C	-0.770460	0.511850	0.025760
4	C	-1.922675	1.277065	-0.143397
5	C	-3.107068	0.577616	-0.221655
6	C	-3.149047	-0.829338	-0.135353
7	C	1.288950	-0.214712	0.302751
8	N	0.559162	0.887024	0.142825
9	H	-2.039260	-2.663049	0.100228
10	H	-1.888837	2.354150	-0.208128
11	H	-4.032072	1.119269	-0.352822
12	H	-4.105398	-1.326800	-0.203850
13	N	0.493485	-1.284957	0.288758
14	C	2.759562	-0.289713	0.492497
15	H	3.205169	0.651478	0.787700
16	H	3.019223	-1.068994	1.200112
17	O	3.374521	-0.723134	-0.789408
18	H	4.208723	-1.215045	-0.662530
19	H	3.517513	-0.004729	-1.435708
20	H	0.810340	-2.239660	0.394177
21	C	0.997832	2.282637	0.101530
22	H	0.567137	2.805121	0.950662
23	H	2.078247	2.339220	0.143642
24	H	0.650141	2.721060	-0.829040

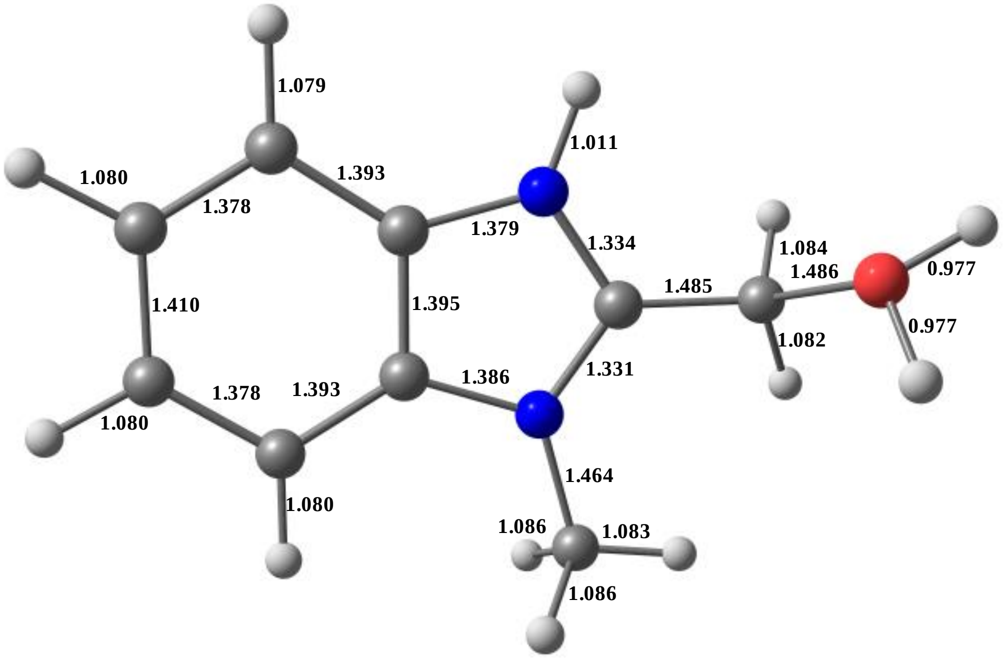

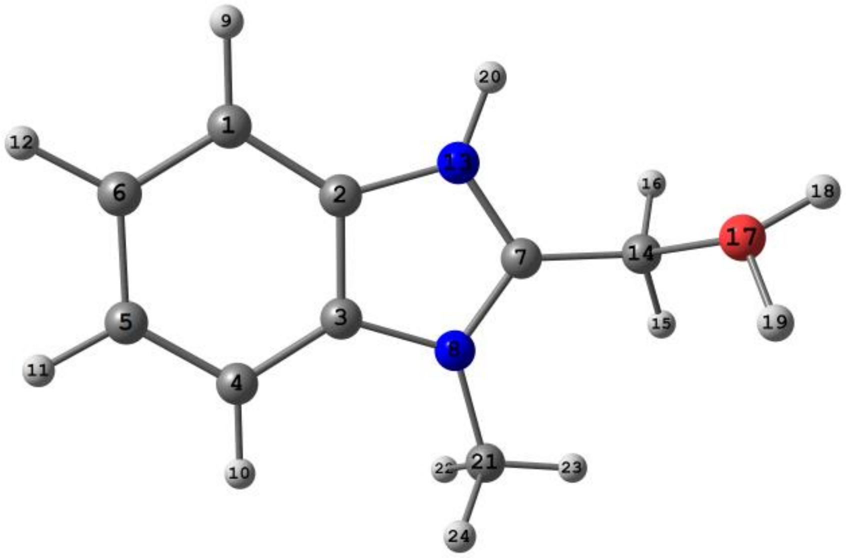

Summary of Natural Population Analysis:

Atom	No	Natural Charge	Natural Population Core	Valence	Rydberg	Total
C	1	-0.19821	1.99902	4.18128	0.01791	6.19821
C	2	0.16170	1.99890	3.81929	0.02011	5.83830
C	3	0.16590	1.99889	3.81704	0.01816	5.83410
C	4	-0.20505	1.99903	4.18879	0.01723	6.20505
C	5	-0.16882	1.99917	4.15061	0.01905	6.16882
C	6	-0.16781	1.99916	4.14968	0.01897	6.16781
C	7	0.46685	1.99918	3.51095	0.02302	5.53315
N	8	-0.33843	1.99911	5.32334	0.01598	7.33843
H	9	0.24168	0.00000	0.75659	0.00172	0.75832
H	10	0.24109	0.00000	0.75724	0.00167	0.75891
H	11	0.22727	0.00000	0.77126	0.00148	0.77273
H	12	0.22712	0.00000	0.77142	0.00146	0.77288
N	13	-0.48089	1.99919	5.46573	0.01597	7.48089
C	14	-0.06105	1.99900	4.04295	0.01910	6.06105
H	15	0.25811	0.00000	0.74041	0.00148	0.74189
H	16	0.26973	0.00000	0.72861	0.00166	0.73027
O	17	-0.63773	1.99972	6.62860	0.00941	8.63773
H	18	0.59238	0.00000	0.40484	0.00278	0.40762
H	19	0.58971	0.00000	0.40748	0.00281	0.41029
H	20	0.47404	0.00000	0.52353	0.00243	0.52596
C	21	-0.35841	1.99928	4.34623	0.01291	6.35841
H	22	0.23672	0.00000	0.76194	0.00134	0.76328
H	23	0.22742	0.00000	0.77133	0.00124	0.77258
H	24	0.23666	0.00000	0.76199	0.00134	0.76334
* Total *		2.00000	23.98965	61.78111	0.22925	86.00000

IV q=2

Energy **E= -458.006787024 h,** **G^{298}= -457.863021 h,** **μ=5.3 D**

Cartesian coordinates, Å

N	atom	x	y	z
1	C	-1.840493	-1.405874	-0.000218
2	C	-0.514482	-0.921929	-0.000158
3	C	-0.220276	0.511022	0.000081
4	C	-1.261962	1.471405	0.000347
5	C	-2.518512	0.970467	0.000232
6	C	-2.805693	-0.455731	-0.000095
7	C	1.683779	-0.621673	-0.000093
8	N	1.089773	0.640421	-0.000005
9	H	-2.050752	-2.463020	-0.000214
10	H	-1.060078	2.530137	0.000721
11	H	-3.359817	1.647429	0.000441
12	H	-3.844148	-0.754107	-0.000087
13	N	0.644404	-1.540648	-0.000118
14	C	2.996113	-0.886136	0.000479
15	H	3.717488	-0.081615	0.000689
16	H	3.349050	-1.907327	0.000840
17	H	0.781997	-2.547351	-0.000237
18	C	1.854219	1.891421	-0.000537
19	H	2.467437	1.921149	0.894837
20	H	2.467479	1.920418	-0.895824
21	H	1.155966	2.718051	-0.000530

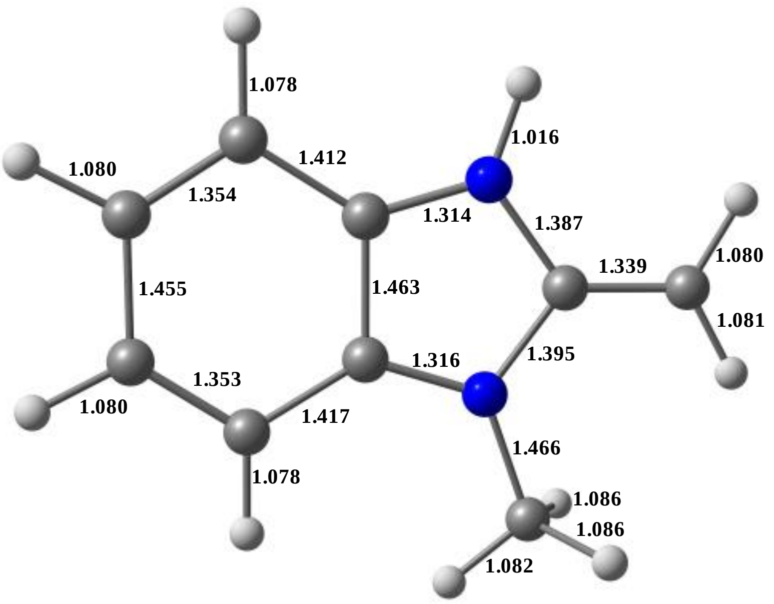

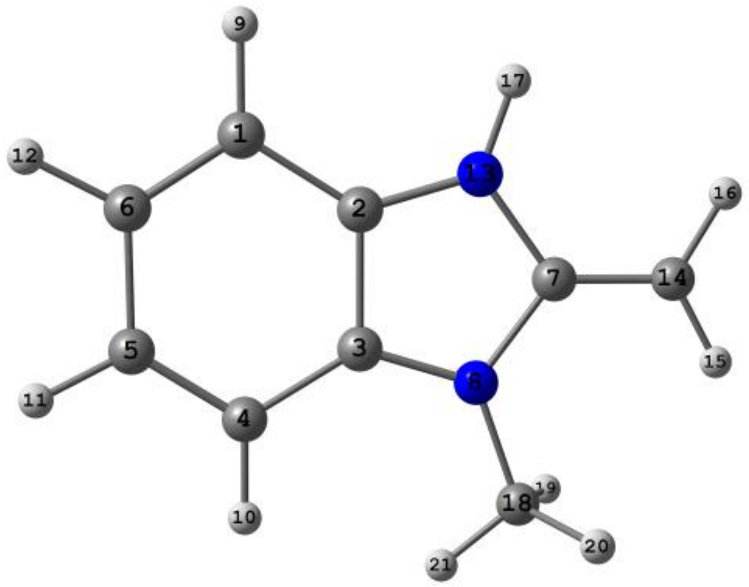

Summary of Natural Population Analysis:

Atom	No	Natural Charge	Natural Population Core	Valence	Rydberg	Total
C	1	-0.22610	1.99901	4.20823	0.01886	6.22610
C	2	0.32413	1.99908	3.65568	0.02111	5.67587
C	3	0.32410	1.99902	3.65724	0.01964	5.67590
C	4	-0.22456	1.99901	4.20749	0.01806	6.22456
C	5	-0.06840	1.99915	4.05076	0.01849	6.06840
C	6	-0.05574	1.99915	4.03819	0.01840	6.05574
C	7	0.32538	1.99912	3.65460	0.02090	5.67462
N	8	-0.28631	1.99909	5.27061	0.01661	7.28631
H	9	0.26548	0.00000	0.73287	0.00166	0.73452
H	10	0.26088	0.00000	0.73745	0.00167	0.73912
H	11	0.24699	0.00000	0.75165	0.00136	0.75301
H	12	0.24661	0.00000	0.75204	0.00135	0.75339
N	13	-0.43040	1.99915	5.41463	0.01662	7.43040
C	14	-0.08334	1.99909	4.07263	0.01162	6.08334
H	15	0.25277	0.00000	0.74607	0.00116	0.74723
H	16	0.25941	0.00000	0.73930	0.00129	0.74059
H	17	0.49275	0.00000	0.50490	0.00235	0.50725
C	18	-0.36824	1.99927	4.35512	0.01385	6.36824
H	19	0.25091	0.00000	0.74794	0.00115	0.74909
H	20	0.25091	0.00000	0.74794	0.00115	0.74909
H	21	0.24278	0.00000	0.75595	0.00127	0.75722
* Total *		2.00000	21.99015	53.80127	0.20858	76.00000

V q=0

Energy **E= -572.977907965 h,   G^{298}= -572.806501 h,   μ=5.4 D**

Cartesian coordinates, Å

N	atom	x	y	z
1	C	2.383886	-1.481565	-0.045499
2	C	1.111077	-0.909491	-0.056933
3	C	0.969061	0.486197	-0.002088
4	C	2.059332	1.349853	0.066892
5	C	3.313911	0.763745	0.078681
6	C	3.474107	-0.631690	0.023176
7	C	-0.989695	-0.504737	-0.108382
8	N	-0.387371	0.721397	-0.037002
9	H	2.507538	-2.554926	-0.088881
10	H	1.936105	2.422898	0.112489
11	H	4.191198	1.393209	0.131488
12	H	4.472707	-1.045324	0.034817
13	N	-0.138059	-1.501380	-0.126253
14	C	-2.476009	-0.708196	-0.104869
15	H	-2.624151	-1.768159	-0.317815
16	O	-3.030979	0.081448	-1.151938
17	H	-3.983707	-0.048363	-1.152183
18	C	-0.989214	2.042801	0.028886
19	H	-0.503648	2.692020	-0.697370
20	H	-2.041985	1.969895	-0.214672
21	H	-0.863339	2.463263	1.025636
22	C	-3.092428	-0.376088	1.248822
23	H	-4.166199	-0.560248	1.218931
24	H	-2.658266	-1.008237	2.022212
25	H	-2.924580	0.667297	1.511528

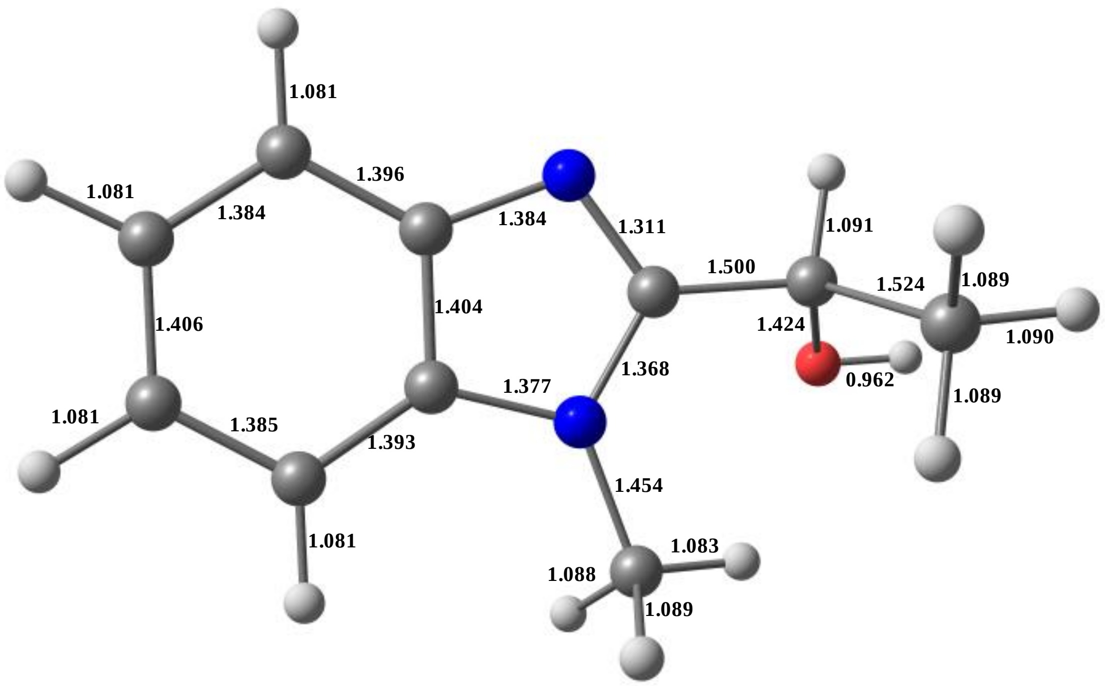

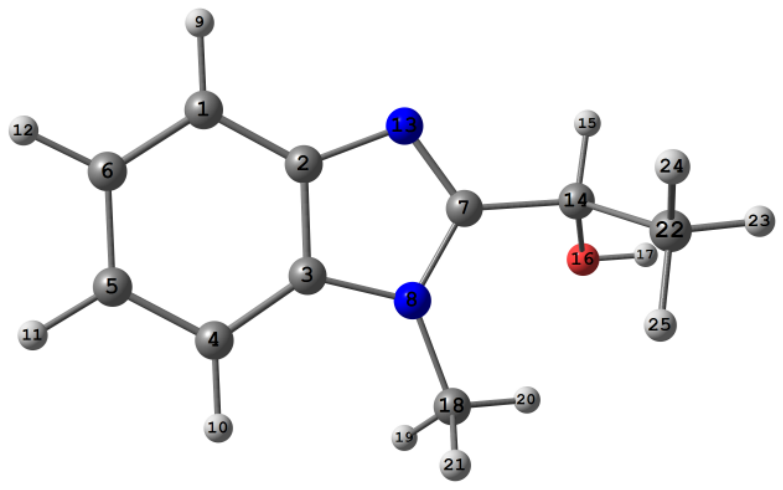

Summary of Natural Population Analysis:

Atom	No	Natural Charge	Natural Population Core	Valence	Rydberg	Total
C	1	-0.20704	1.99902	4.18853	0.01949	6.20704
C	2	0.11161	1.99887	3.86434	0.02517	5.88839
C	3	0.14648	1.99890	3.83640	0.01822	5.85352
C	4	-0.23873	1.99904	4.22196	0.01774	6.23873
C	5	-0.21069	1.99917	4.19153	0.02000	6.21069
C	6	-0.22842	1.99916	4.20909	0.02018	6.22842
C	7	0.45514	1.99911	3.51267	0.03307	5.54486
N	8	-0.43316	1.99911	5.41473	0.01931	7.43316
H	9	0.21549	0.00000	0.78239	0.00213	0.78451
H	10	0.22043	0.00000	0.77761	0.00196	0.77957
H	11	0.21031	0.00000	0.78808	0.00161	0.78969
H	12	0.20968	0.00000	0.78872	0.00161	0.79032
N	13	-0.56992	1.99936	5.53293	0.03763	7.56992
C	14	0.09492	1.99901	3.88059	0.02548	5.90508
H	15	0.18955	0.00000	0.80759	0.00286	0.81045
O	16	-0.77781	1.99977	6.76488	0.01316	8.77781
H	17	0.49059	0.00000	0.50481	0.00460	0.50941
C	18	-0.36572	1.99931	4.35286	0.01355	6.36572
H	19	0.21024	0.00000	0.78800	0.00177	0.78976
H	20	0.23197	0.00000	0.76638	0.00165	0.76803
H	21	0.20617	0.00000	0.79221	0.00162	0.79383
C	22	-0.59641	1.99928	4.58593	0.01120	6.59641
H	23	0.21142	0.00000	0.78693	0.00165	0.78858
H	24	0.21825	0.00000	0.78010	0.00165	0.78175
H	25	0.20566	0.00000	0.79278	0.00156	0.79434
* Total *		0.00000	25.98911	67.71203	0.29886	94.00000

V q=2

Energy **E= -573.797055441 h, G^{298}= -573.59919 h, μ=15.7 D**

Cartesian coordinates, Å

N	atom	x	y	z
1	C	-2.116991	-1.683796	-0.050505
2	C	-0.995716	-0.859491	-0.006556
3	C	-1.100821	0.530025	0.028462
4	C	-2.335553	1.175467	0.035345
5	C	-3.445979	0.359621	-0.004754
6	C	-3.338181	-1.044733	-0.049102
7	C	1.038926	0.007676	0.042057
8	N	0.192766	1.034045	0.044122
9	H	-2.027851	-2.758584	-0.083558
10	H	-2.419304	2.251028	0.073566
11	H	-4.428472	0.807911	-0.002012
12	H	-4.241206	-1.636466	-0.080763
13	N	0.356394	-1.137126	0.011903
14	C	2.529154	0.087912	0.095216
15	H	2.864311	1.117085	0.147753
16	O	3.002348	-0.452504	-1.230470
17	H	3.941300	-0.722791	-1.214958
18	H	2.852935	0.144024	-1.990279
19	H	0.769932	-2.059233	-0.015295
20	C	0.495936	2.465696	0.075815
21	H	0.362791	2.838586	1.086898
22	H	1.509413	2.641930	-0.262485
23	H	-0.189358	2.962675	-0.603413
24	C	3.145931	-0.765771	1.173654
25	H	4.227366	-0.651633	1.170737
26	H	2.769679	-0.413293	2.131899
27	H	2.885328	-1.815283	1.055706

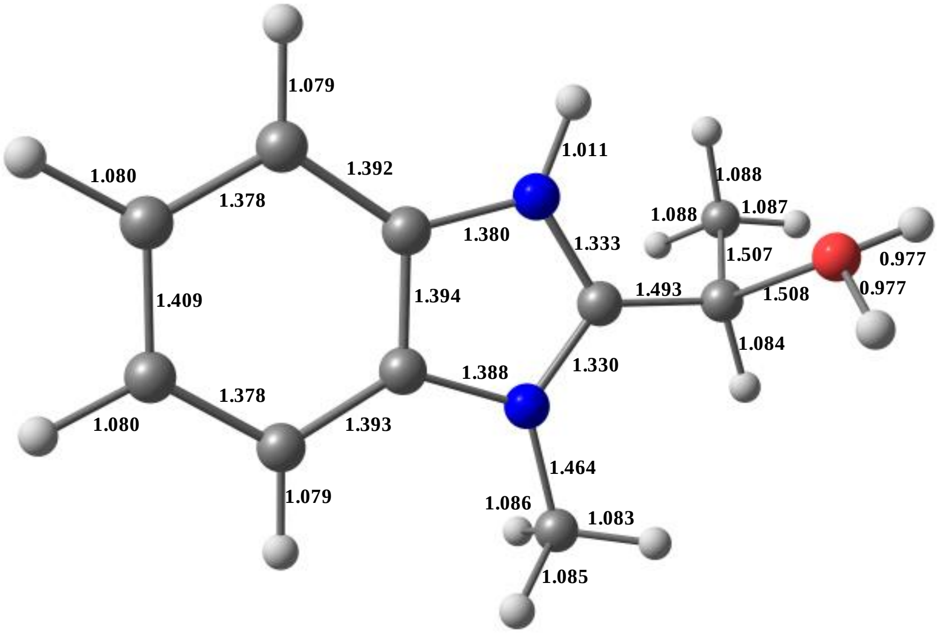

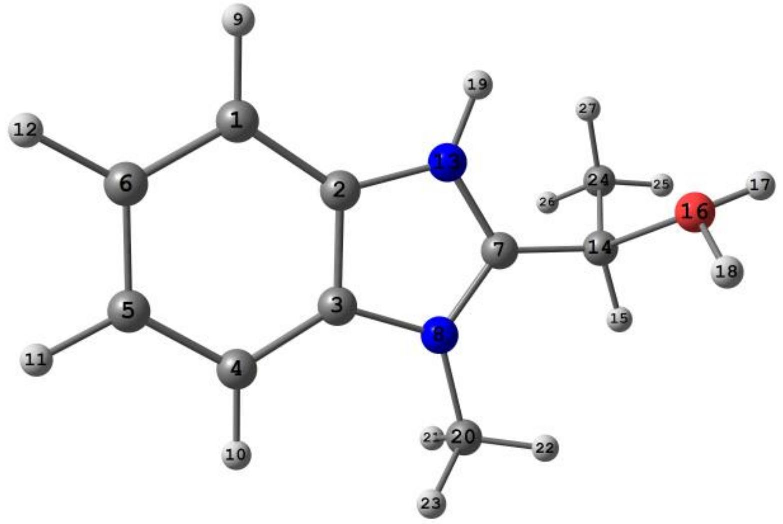

Summary of Natural Population Analysis:

Atom	No	Natural Charge	Natural Population Core	Valence	Rydberg	Total
C	1	-0.19836	1.99902	4.18150	0.01784	6.19836
C	2	0.16089	1.99889	3.81996	0.02025	5.83911
C	3	0.16524	1.99889	3.81776	0.01811	5.83476
C	4	-0.20452	1.99903	4.18825	0.01725	6.20452
C	5	-0.17089	1.99917	4.15268	0.01904	6.17089
C	6	-0.16893	1.99916	4.15079	0.01898	6.16893
C	7	0.47616	1.99912	3.49895	0.02577	5.52384
N	8	-0.34047	1.99910	5.32518	0.01618	7.34047
H	9	0.24121	0.00000	0.75706	0.00173	0.75879
H	10	0.24043	0.00000	0.75788	0.00169	0.75957
H	11	0.22701	0.00000	0.77151	0.00148	0.77299
H	12	0.22702	0.00000	0.77151	0.00147	0.77298
N	13	-0.48831	1.99918	5.47347	0.01565	7.48831
C	14	0.11310	1.99897	3.86893	0.01900	5.88690
H	15	0.25504	0.00000	0.74304	0.00192	0.74496
O	16	-0.65310	1.99973	6.64271	0.01066	8.65310
H	17	0.58662	0.00000	0.41060	0.00278	0.41338
H	18	0.58879	0.00000	0.40853	0.00268	0.41121
H	19	0.47124	0.00000	0.52610	0.00266	0.52876
C	20	-0.35873	1.99928	4.34668	0.01277	6.35873
H	21	0.23725	0.00000	0.76150	0.00124	0.76275
H	22	0.22778	0.00000	0.77093	0.00129	0.77222
H	23	0.23593	0.00000	0.76270	0.00137	0.76407
C	24	-0.61687	1.99926	4.60270	0.01492	6.61687
H	25	0.24669	0.00000	0.75186	0.00146	0.75331
H	26	0.26494	0.00000	0.73388	0.00118	0.73506
H	27	0.23482	0.00000	0.76361	0.00156	0.76518
* Total *		2.00000	25.98879	67.76029	0.25093	94.00000

VI q=2

Energy **E= -497.328171971 h, G^{298}= -497.159717 h, μ=5.3 D**

Cartesian coordinates, Å

N	atom	x	y	z
1	C	1.820935	-1.693335	0.004030
2	C	0.663620	-0.888949	0.003595
3	C	0.736576	0.555123	0.000649
4	C	1.983855	1.222709	-0.005671
5	C	3.079522	0.422860	-0.006593
6	C	3.000145	-1.020367	-0.001233
7	C	-1.387187	-0.057718	0.004968
8	N	-0.511630	1.010218	0.004286
9	H	1.754111	-2.769300	0.007200
10	H	2.056717	2.298087	-0.010075
11	H	4.061789	0.871669	-0.011734
12	H	3.926947	-1.575028	-0.002166
13	N	-0.624184	-1.199418	0.004928
14	C	-2.747644	0.029836	0.001819
15	H	-3.163749	1.030882	0.010364
16	H	-1.000728	-2.141202	0.013485
17	C	-0.925861	2.418549	0.002854
18	H	-1.498649	2.615237	-0.898329
19	H	-1.514505	2.611960	0.894431
20	H	-0.034303	3.031693	0.012315
21	C	-3.680334	-1.091378	-0.011863
22	H	-4.315180	-1.008726	0.877565
23	H	-4.361019	-0.952741	-0.857965
24	H	-3.222498	-2.072102	-0.054915

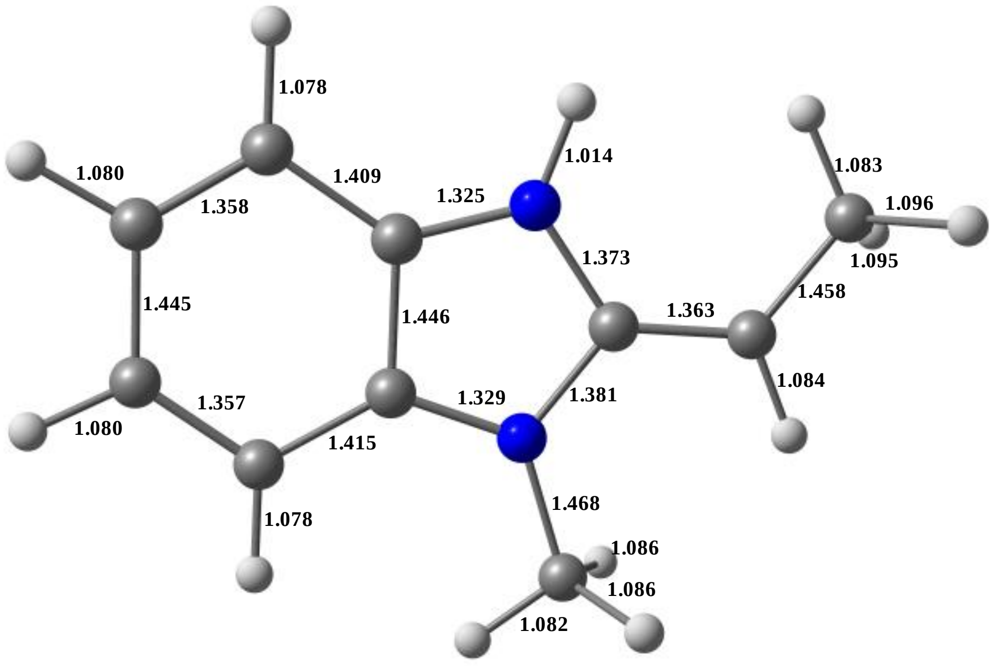

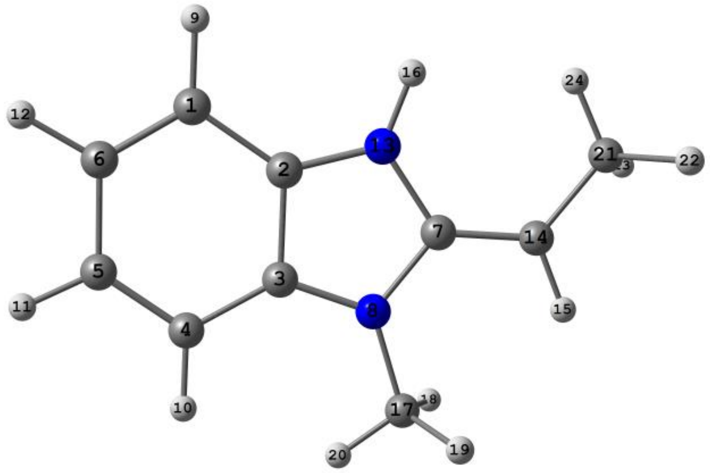

Summary of Natural Population Analysis:

Atom	No	Natural Charge	Natural Population Core	Valence	Rydberg	Total
C	1	-0.21894	1.99902	4.20140	0.01853	6.21894
C	2	0.28990	1.99906	3.69022	0.02082	5.71010
C	3	0.28883	1.99900	3.69299	0.01918	5.71117
C	4	-0.21781	1.99902	4.20100	0.01779	6.21781
C	5	-0.09083	1.99916	4.07311	0.01857	6.09083
C	6	-0.07882	1.99916	4.06116	0.01850	6.07882
C	7	0.32883	1.99904	3.65037	0.02175	5.67117
N	8	-0.28140	1.99910	5.26644	0.01586	7.28140
H	9	0.26040	0.00000	0.73794	0.00166	0.73960
H	10	0.25594	0.00000	0.74238	0.00168	0.74406
H	11	0.24292	0.00000	0.75570	0.00138	0.75708
H	12	0.24268	0.00000	0.75595	0.00137	0.75732
N	13	-0.43190	1.99916	5.41722	0.01551	7.43190
C	14	0.14082	1.99910	3.84718	0.01290	5.85918
H	15	0.25321	0.00000	0.74514	0.00165	0.74679
H	16	0.48537	0.00000	0.51199	0.00264	0.51463
C	17	-0.36918	1.99927	4.35608	0.01383	6.36918
H	18	0.24871	0.00000	0.75013	0.00116	0.75129
H	19	0.24841	0.00000	0.75042	0.00116	0.75159
H	20	0.24169	0.00000	0.75702	0.00129	0.75831
C	21	-0.65066	1.99925	4.63835	0.01306	6.65066
H	22	0.29071	0.00000	0.70791	0.00137	0.70929
H	23	0.28810	0.00000	0.71054	0.00136	0.71190
H	24	0.23301	0.00000	0.76553	0.00146	0.76699
* Total *		2.00000	23.98932	59.78618	0.22449	84.00000

VII q=0

Energy **E= -533.659161533 h, G^{298}= -533.514487 h, μ=4.7 D**

Cartesian coordinates, Å

N	atom	x	y	z
1	C	-2.501009	-1.276892	0.000307
2	C	-1.196917	-0.779874	0.000043
3	C	-0.976298	0.609044	-0.000223
4	C	-2.016719	1.532544	-0.000186
5	C	-3.302877	1.020118	0.000081
6	C	-3.541658	-0.364814	0.000310
7	C	0.920382	-0.509137	-0.000346
8	N	0.395332	0.744893	-0.000403
9	H	-2.684450	-2.342405	0.000517
10	H	-1.831136	2.596994	-0.000351
11	H	-4.143042	1.700135	0.000119
12	H	-4.562154	-0.721462	0.000524
13	H	0.940073	1.592008	-0.000518
14	N	0.016806	-1.453104	0.000062
15	C	2.398468	-0.729503	-0.000753
16	H	2.678352	-1.311783	0.883481
17	H	2.678198	-1.309451	-0.886602
18	O	3.032680	0.524704	0.000751
19	C	4.442628	0.401252	0.000425
20	H	4.783378	-0.134534	0.889885
21	H	4.783320	-0.131868	-0.890650
22	H	4.855057	1.405781	0.002020

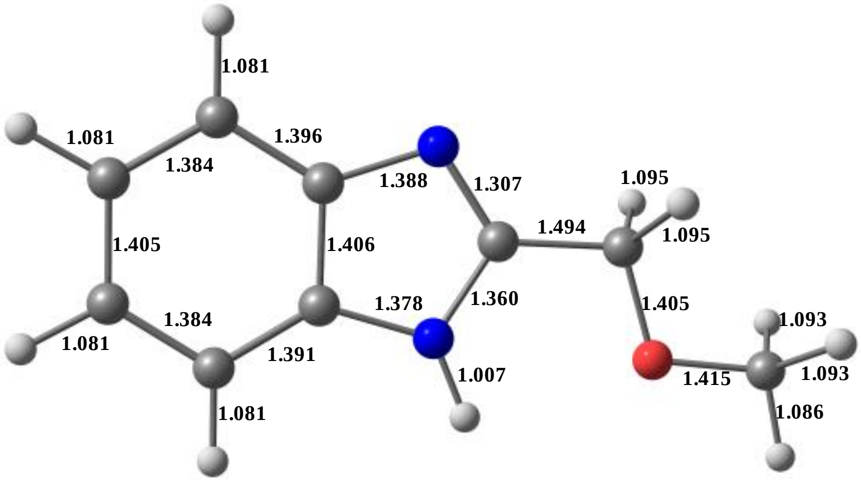

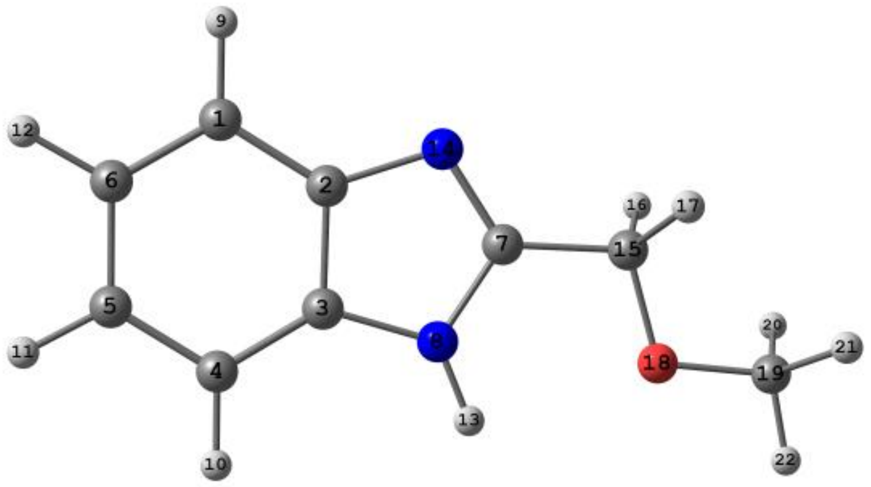

Summary of Natural Population Analysis:

Atom	No	Natural Charge	Natural Population: Core	Valence	Rydberg	Total
C	1	-0.21319	1.99902	4.19442	0.01975	6.21319
C	2	0.11461	1.99888	3.86153	0.02499	5.88539
C	3	0.12821	1.99890	3.85364	0.01925	5.87179
C	4	-0.23038	1.99903	4.21317	0.01819	6.23038
C	5	-0.21574	1.99916	4.19683	0.01975	6.21574
C	6	-0.22658	1.99916	4.20732	0.02010	6.22658
C	7	0.44192	1.99923	3.52989	0.02896	5.55808
N	8	-0.57162	1.99920	5.55235	0.02007	7.57162
H	9	0.21548	0.00000	0.78236	0.00217	0.78452
H	10	0.22032	0.00000	0.77778	0.00189	0.77968
H	11	0.21009	0.00000	0.78830	0.00161	0.78991
H	12	0.20937	0.00000	0.78902	0.00161	0.79063
H	13	0.44693	0.00000	0.55037	0.00271	0.55307
N	14	-0.58202	1.99934	5.54547	0.03720	7.58202
C	15	-0.04886	1.99908	4.02897	0.02082	6.04886
H	16	0.19470	0.00000	0.80284	0.00246	0.80530
H	17	0.19470	0.00000	0.80285	0.00246	0.80530
O	18	-0.62303	1.99976	6.60459	0.01868	8.62303
C	19	-0.19123	1.99925	4.17656	0.01542	6.19123
H	20	0.16796	0.00000	0.82973	0.00231	0.83204
H	21	0.16797	0.00000	0.82972	0.00231	0.83203
H	22	0.19040	0.00000	0.80829	0.00131	0.80960
* Total *		0.00000	23.99001	61.72597	0.28401	86.00000

VII q=1

Energy **E= -534.10265871 h,** **G^{298}= -533.944512 h,** **μ=4.3 D**

Cartesian coordinates, Å

N	atom	x	y	z
1	C	-1.994322	-1.557197	0.000073
2	C	-0.978950	-0.609604	0.000043
3	C	-1.247277	0.758728	-0.000063
4	C	-2.546023	1.250847	-0.000162
5	C	-3.557728	0.309119	-0.000147
6	C	-3.287356	-1.069459	-0.000021
7	C	0.953576	0.478376	0.000146
8	N	-0.008937	1.394119	0.000045
9	H	-1.780259	-2.615157	0.000179
10	H	-2.750379	2.310511	-0.000257
11	H	-4.585278	0.642342	-0.000210
12	H	-4.112976	-1.765778	0.000021
13	H	0.150281	2.390974	-0.000246
14	N	0.406170	-0.727669	0.000163
15	C	2.423798	0.717704	0.000387
16	H	2.690848	1.301897	-0.886711
17	H	2.690672	1.301118	0.888055
18	O	3.030891	-0.540978	-0.000066
19	C	4.448741	-0.448119	-0.000361
20	H	4.796750	0.076558	-0.892110
21	H	4.797139	0.076646	0.891186
22	H	4.830552	-1.463688	-0.000381
23	H	0.958134	-1.575114	0.000176

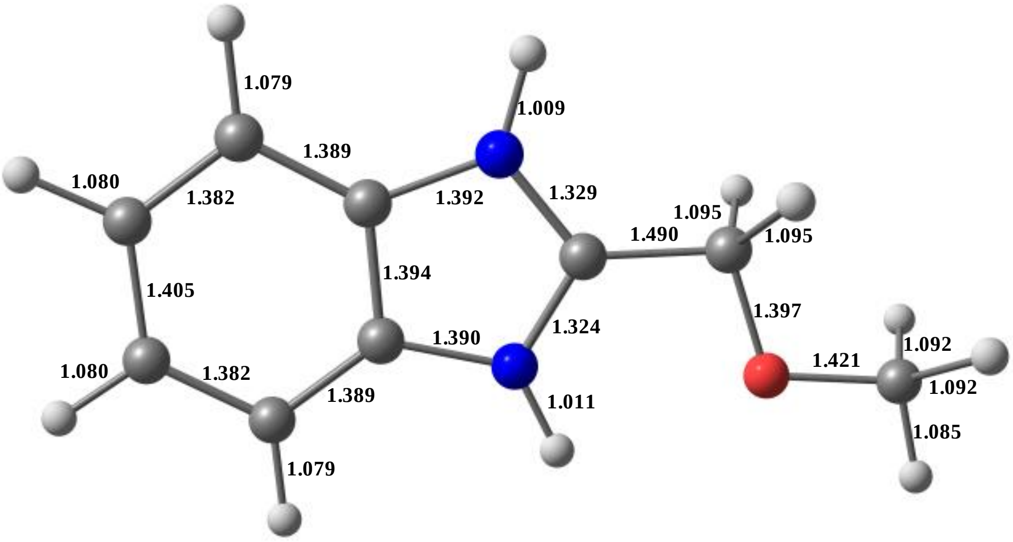

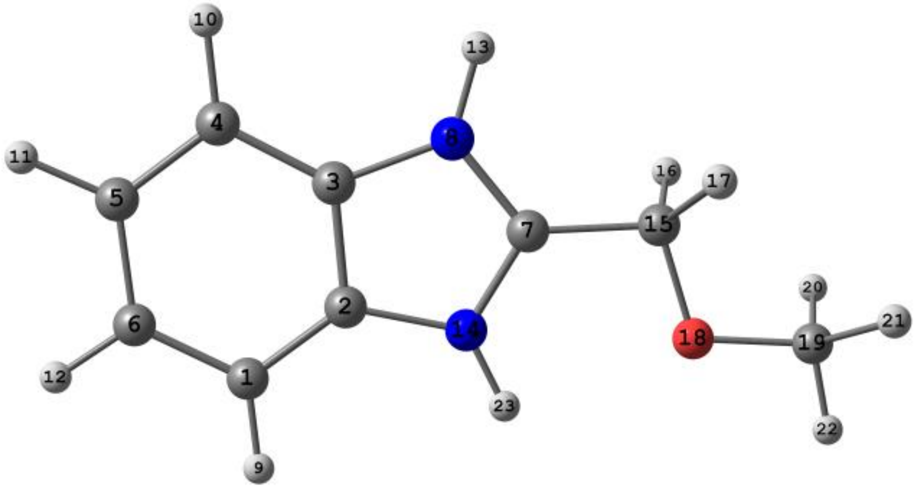

Summary of Natural Population Analysis:

Atom	No	Natural Charge	Natural Population Core	Valence	Rydberg	Total
C	1	-0.20276	1.99902	4.18572	0.01802	6.20276
C	2	0.14340	1.99888	3.83784	0.01988	5.85660
C	3	0.14803	1.99888	3.83324	0.01985	5.85197
C	4	-0.20592	1.99902	4.18872	0.01817	6.20592
C	5	-0.18496	1.99916	4.16666	0.01913	6.18496
C	6	-0.18716	1.99916	4.16888	0.01912	6.18716
C	7	0.53940	1.99926	3.43718	0.02416	5.46060
N	8	-0.51169	1.99918	5.49739	0.01512	7.51169
H	9	0.23636	0.00000	0.76188	0.00176	0.76364
H	10	0.23635	0.00000	0.76188	0.00177	0.76365
H	11	0.22251	0.00000	0.77600	0.00149	0.77749
H	12	0.22251	0.00000	0.77599	0.00150	0.77749
H	13	0.46631	0.00000	0.53133	0.00236	0.53369
N	14	-0.50684	1.99917	5.49124	0.01644	7.50684
C	15	-0.05934	1.99908	4.03922	0.02104	6.05934
H	16	0.21788	0.00000	0.77974	0.00238	0.78212
H	17	0.21789	0.00000	0.77973	0.00238	0.78211
O	18	-0.61680	1.99976	6.59833	0.01870	8.61680
C	19	-0.19242	1.99924	4.17799	0.01519	6.19242
H	20	0.17413	0.00000	0.82378	0.00209	0.82587
H	21	0.17413	0.00000	0.82378	0.00209	0.82587
H	22	0.19680	0.00000	0.80198	0.00121	0.80320
H	23	0.47219	0.00000	0.52506	0.00275	0.52781
* Total *		1.00000	23.98983	61.76358	0.24659	86.00000

VII q=2

Energy **E= -534.47886956 h,** **G^{298}= -534.307497 h,** **μ=15.7 D**

Cartesian coordinates, Å

N	atom	x	y	z
1	C	-2.469728	-1.330548	-0.183826
2	C	-1.231910	-0.728133	0.029489
3	C	-1.093980	0.656919	0.138074
4	C	-2.184340	1.517356	0.035301
5	C	-3.407431	0.920374	-0.178085
6	C	-3.547548	-0.478601	-0.285283
7	C	0.903819	-0.263184	0.373763
8	N	0.251462	0.893792	0.357506
9	H	-2.571974	-2.401814	-0.264142
10	H	-2.070041	2.587058	0.121214
11	H	-4.287465	1.540750	-0.262987
12	H	-4.531046	-0.893430	-0.450162
13	H	0.680508	1.801180	0.484350
14	N	0.040115	-1.252460	0.177173
15	C	2.363200	-0.433122	0.597192
16	H	2.702536	0.160851	1.439959
17	H	2.635131	-1.476160	0.726640
18	O	3.078211	0.112268	-0.565191
19	C	4.520335	0.397089	-0.342403
20	H	4.540075	1.189401	0.395006
21	H	4.996472	-0.514240	0.000349
22	H	4.893951	0.730762	-1.301501
23	H	0.287766	-2.233525	0.150620
24	H	2.942858	-0.427193	-1.365896

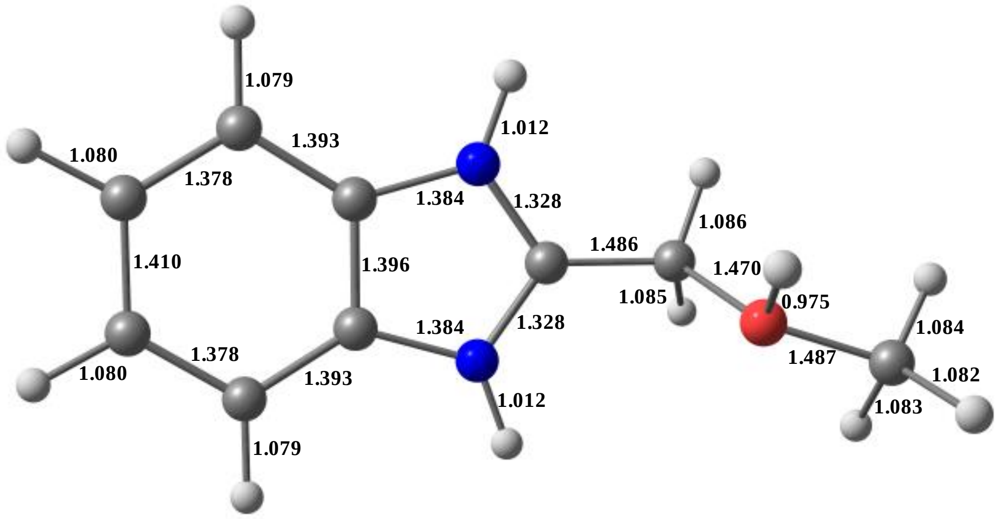

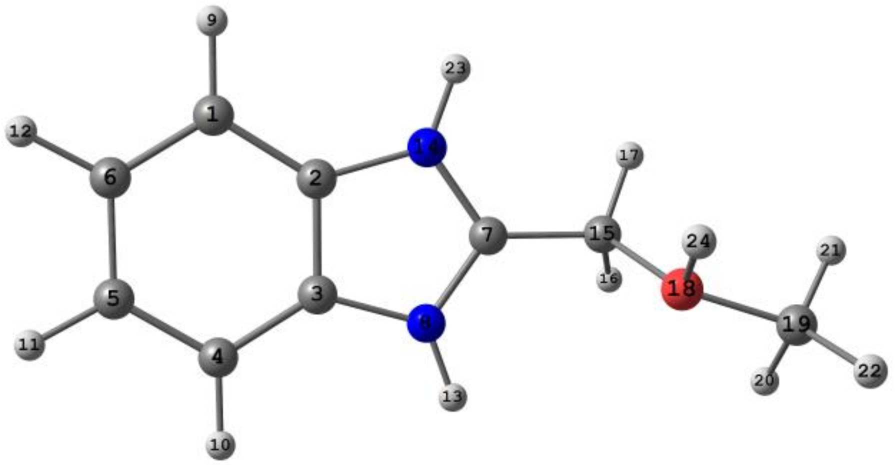

Summary of Natural Population Analysis:

Atom	No	Natural Charge	Core	Valence	Rydberg	Total
C	1	-0.19754	1.99902	4.18049	0.01803	6.19754
C	2	0.15306	1.99889	3.82809	0.01996	5.84694
C	3	0.15773	1.99890	3.82353	0.01984	5.84227
C	4	-0.19999	1.99902	4.18288	0.01809	6.19999
C	5	-0.16875	1.99916	4.15068	0.01891	6.16875
C	6	-0.17133	1.99916	4.15323	0.01894	6.17133
C	7	0.46618	1.99926	3.50995	0.02460	5.53382
N	8	-0.47981	1.99918	5.46430	0.01633	7.47981
H	9	0.24186	0.00000	0.75642	0.00173	0.75814
H	10	0.24181	0.00000	0.75646	0.00173	0.75819
H	11	0.22719	0.00000	0.77134	0.00146	0.77281
H	12	0.22718	0.00000	0.77135	0.00147	0.77282
H	13	0.47545	0.00000	0.52196	0.00259	0.52455
N	14	-0.47304	1.99918	5.45826	0.01560	7.47304
C	15	-0.06018	1.99902	4.04234	0.01882	6.06018
H	16	0.26457	0.00000	0.73365	0.00178	0.73543
H	17	0.25838	0.00000	0.74006	0.00156	0.74162
O	18	-0.51446	1.99974	6.50356	0.01117	8.51446
C	19	-0.18455	1.99921	4.17501	0.01034	6.18455
H	20	0.22595	0.00000	0.77294	0.00112	0.77405
H	21	0.22006	0.00000	0.77883	0.00110	0.77994
H	22	0.23612	0.00000	0.76282	0.00106	0.76388
H	23	0.47602	0.00000	0.52157	0.00241	0.52398
H	24	0.57810	0.00000	0.41888	0.00302	0.42190
* Total *		2.00000	23.98976	61.77859	0.23166	86.00000

VIII q=0

Energy **E= -532.452416636 h, G^{298}= -532.331442 h, μ=8.3 D**

Cartesian coordinates, Å

N	atom	x	y	z
1	C	-1.824239	-1.606975	0.000002
2	C	-0.625538	-0.879619	-0.000019
3	C	-0.652233	0.529619	-0.000032
4	C	-1.849850	1.251989	0.000000
5	C	-3.014376	0.514778	0.000023
6	C	-3.004088	-0.897889	0.000017
7	C	1.400240	-0.232574	-0.000039
8	N	0.661104	0.920616	-0.000038
9	H	-1.809721	-2.687695	0.000011
10	H	-1.871253	2.331903	0.000042
11	H	-3.963869	1.031158	0.000054
12	H	-3.946495	-1.426784	0.000029
13	N	0.671562	-1.325539	-0.000022
14	C	2.876019	-0.209435	0.000000
15	O	3.544967	-1.212102	0.000049
16	H	3.343515	0.785971	-0.000026
17	C	1.154691	2.291823	0.000026
18	H	1.747386	2.483151	0.890901
19	H	1.747730	2.483077	-0.890633
20	H	0.300558	2.960202	-0.000220

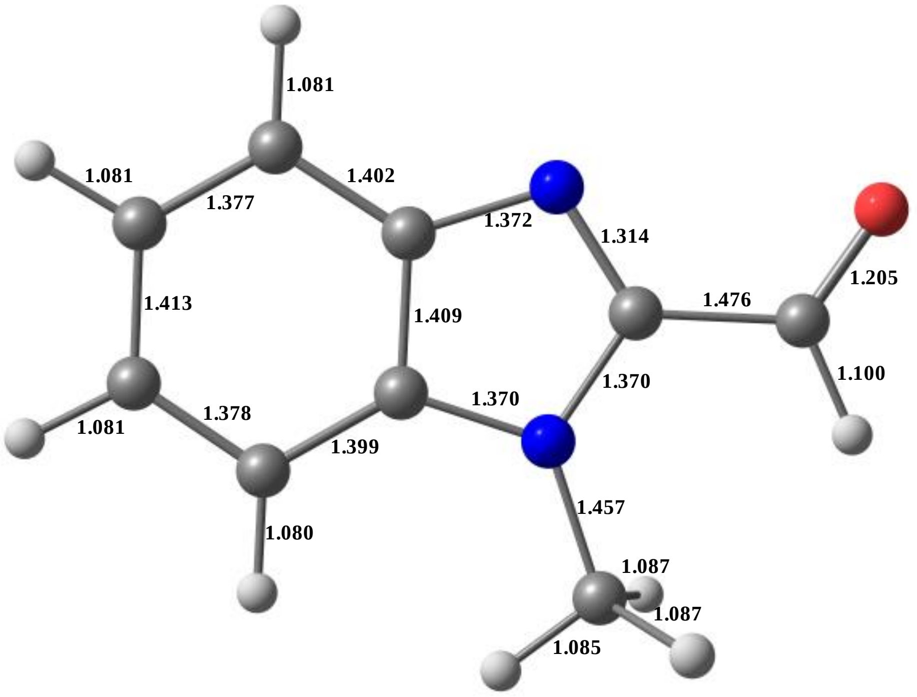

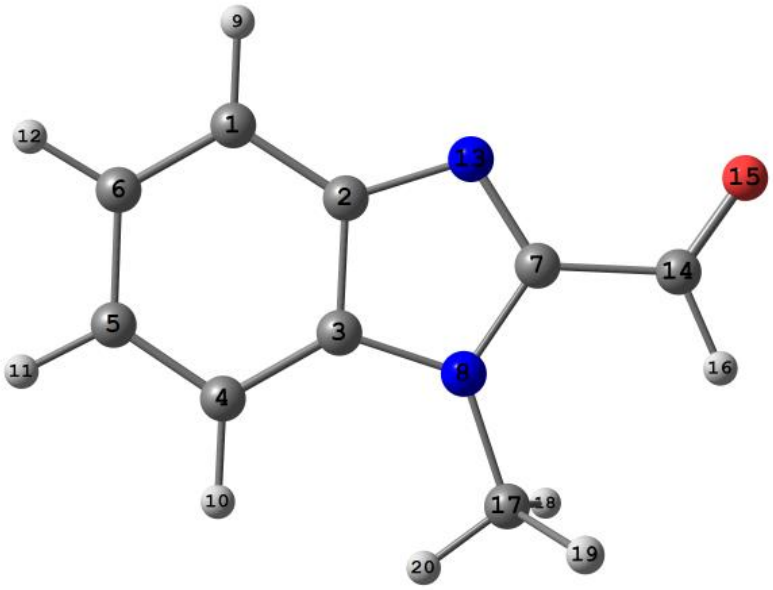

Summary of Natural Population Analysis:

Atom	No	Natural Charge	Natural Population Core	Valence	Rydberg	Total
C	1	-0.19542	1.99902	4.17700	0.01940	6.19542
C	2	0.11351	1.99890	3.86247	0.02512	5.88649
C	3	0.16494	1.99891	3.81744	0.01871	5.83506
C	4	-0.23625	1.99903	4.21926	0.01796	6.23625
C	5	-0.18913	1.99917	4.17036	0.01961	6.18913
C	6	-0.21797	1.99916	4.19886	0.01995	6.21797
C	7	0.31025	1.99915	3.66320	0.02740	5.68975
N	8	-0.40132	1.99914	5.38531	0.01687	7.40132
H	9	0.22055	0.00000	0.77739	0.00206	0.77945
H	10	0.22314	0.00000	0.77486	0.00200	0.77686
H	11	0.21426	0.00000	0.78419	0.00156	0.78574
H	12	0.21381	0.00000	0.78461	0.00158	0.78619
N	13	-0.50346	1.99936	5.46727	0.03682	7.50346
C	14	0.43133	1.99938	3.53856	0.03073	5.56867
O	15	-0.57115	1.99976	6.54369	0.02770	8.57115
H	16	0.12962	0.00000	0.86848	0.00190	0.87038
C	17	-0.36302	1.99930	4.34990	0.01382	6.36302
H	18	0.21771	0.00000	0.78089	0.00140	0.78229
H	19	0.21771	0.00000	0.78089	0.00140	0.78229
H	20	0.22089	0.00000	0.77763	0.00148	0.77911
* Total *		0.00000	23.99029	59.72224	0.28747	84.00000

VIII q=2

Energy **E= -533.261671252 h, G^{298}= -533.113079 h, μ=10.7 D**

Cartesian coordinates, Å

N	atom	x	y	z
1	C	-1.816222	-1.663875	-0.006540
2	C	-0.653213	-0.878650	-0.006052
3	C	-0.706333	0.541687	-0.002900
4	C	-1.930752	1.233197	0.011137
5	C	-3.051111	0.454491	0.013755
6	C	-2.994608	-0.974921	0.002071
7	C	1.394800	-0.079231	-0.012353
8	N	0.568816	0.991347	-0.015220
9	H	-1.766524	-2.741012	-0.013688
10	H	-1.977049	2.310653	0.025776
11	H	-4.022269	0.926621	0.026244
12	H	-3.925625	-1.522298	0.001435
13	N	0.653060	-1.207371	-0.010179
14	C	2.800063	-0.049740	0.006411
15	O	3.406452	-1.160878	0.016457
16	H	3.349836	0.884953	0.016583
17	C	0.956649	2.406812	-0.004646
18	H	0.949614	2.763113	1.021184
19	H	1.938583	2.520506	-0.447269
20	H	0.235596	2.950730	-0.605122
21	H	1.040100	-2.145300	-0.013614
22	H	4.377351	-1.087383	0.029301

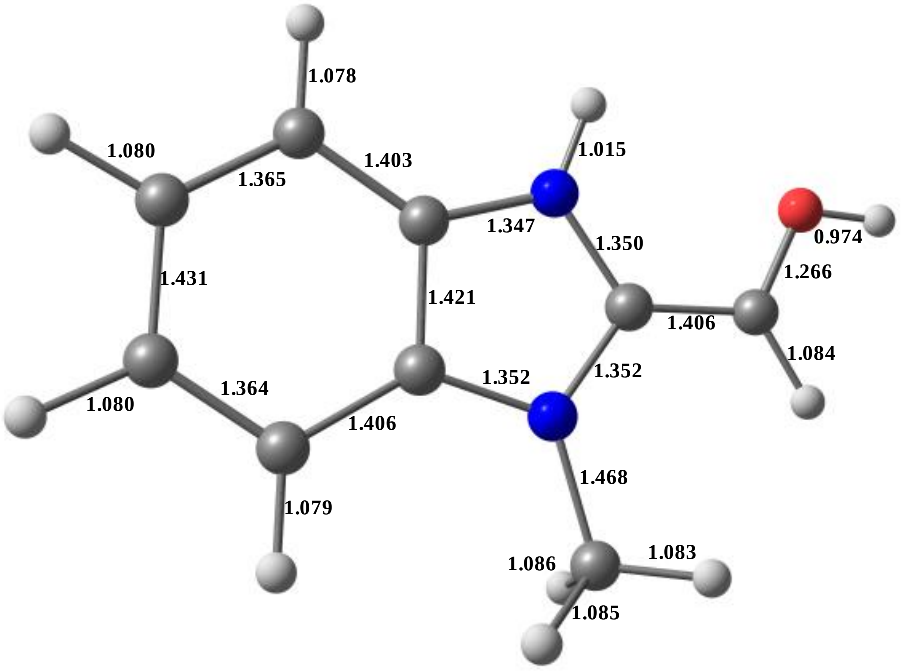

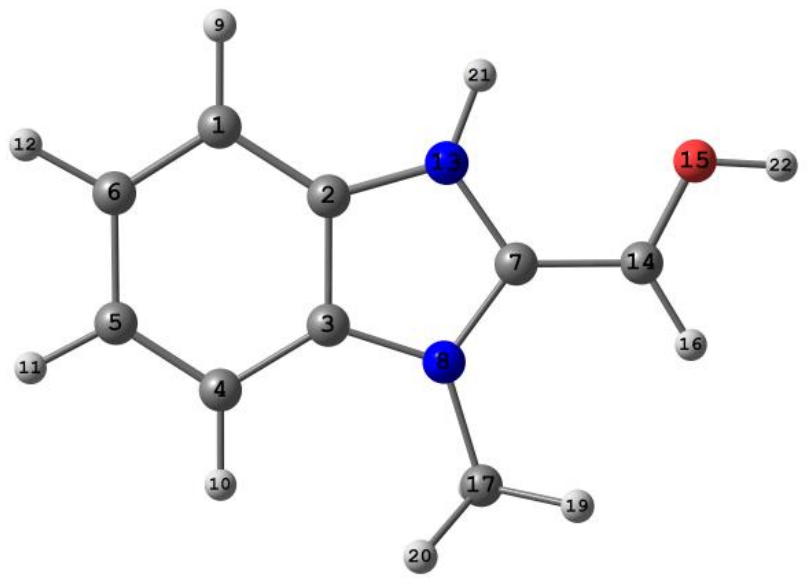

Summary of Natural Population Analysis:

Atom	No	Natural Charge	Natural Population: Core	Valence	Rydberg	Total
C	1	-0.20835	1.99902	4.19108	0.01825	6.20835
C	2	0.23986	1.99900	3.74072	0.02041	5.76014
C	3	0.23676	1.99897	3.74541	0.01886	5.76324
C	4	-0.21018	1.99902	4.19370	0.01745	6.21018
C	5	-0.11796	1.99916	4.10005	0.01875	6.11796
C	6	-0.10939	1.99916	4.09161	0.01861	6.10939
C	7	0.32529	1.99918	3.65231	0.02322	5.67471
N	8	-0.28903	1.99912	5.27400	0.01592	7.28903
H	9	0.25465	0.00000	0.74368	0.00167	0.74535
H	10	0.25265	0.00000	0.74570	0.00165	0.74735
H	11	0.23809	0.00000	0.76050	0.00141	0.76191
H	12	0.23797	0.00000	0.76063	0.00140	0.76203
N	13	-0.44039	1.99918	5.42517	0.01604	7.44039
C	14	0.46513	1.99919	3.51389	0.02179	5.53487
O	15	-0.54461	1.99969	6.52146	0.02346	8.54461
H	16	0.24736	0.00000	0.75136	0.00128	0.75264
C	17	-0.36320	1.99927	4.35071	0.01321	6.36320
H	18	0.24945	0.00000	0.74942	0.00113	0.75055
H	19	0.23578	0.00000	0.76293	0.00129	0.76422
H	20	0.24430	0.00000	0.75442	0.00128	0.75570
H	21	0.48783	0.00000	0.50958	0.00258	0.51217
H	22	0.56797	0.00000	0.42961	0.00242	0.43203
* Total *		2.00000	23.98998	59.76795	0.24208	84.00000

IX q=0

Energy **E= -571.774355517 h, G^{298}= -571.627163 h, μ=2.5 D**

Cartesian coordinates, Å

N	atom	x	y	z
1	C	2.126345	-1.614131	-0.002087
2	C	0.933311	-0.879894	-0.000702
3	C	0.965193	0.527392	0.001001
4	C	2.166966	1.241857	0.000881
5	C	3.329108	0.498749	-0.000505
6	C	3.311917	-0.912385	-0.001873
7	C	-1.093181	-0.218652	0.000478
8	N	-0.347516	0.929636	0.002255
9	H	2.105467	-2.694863	-0.003313
10	H	2.195607	2.321566	0.000910
11	H	4.280767	1.011285	-0.000932
12	H	4.251069	-1.447081	-0.002998
13	N	-0.369070	-1.315758	-0.000686
14	C	-2.578999	-0.232221	-0.002434
15	O	-3.205059	0.805744	-0.013828
16	C	-0.809302	2.311627	0.006061
17	H	-1.379309	2.521928	-0.893811
18	H	-1.433563	2.497225	0.874377
19	H	0.063460	2.955394	0.041267
20	C	-3.232550	-1.582196	0.008049
21	H	-2.913707	-2.140680	0.887342
22	H	-4.310386	-1.456142	0.007960
23	H	-2.915680	-2.152602	-0.864375

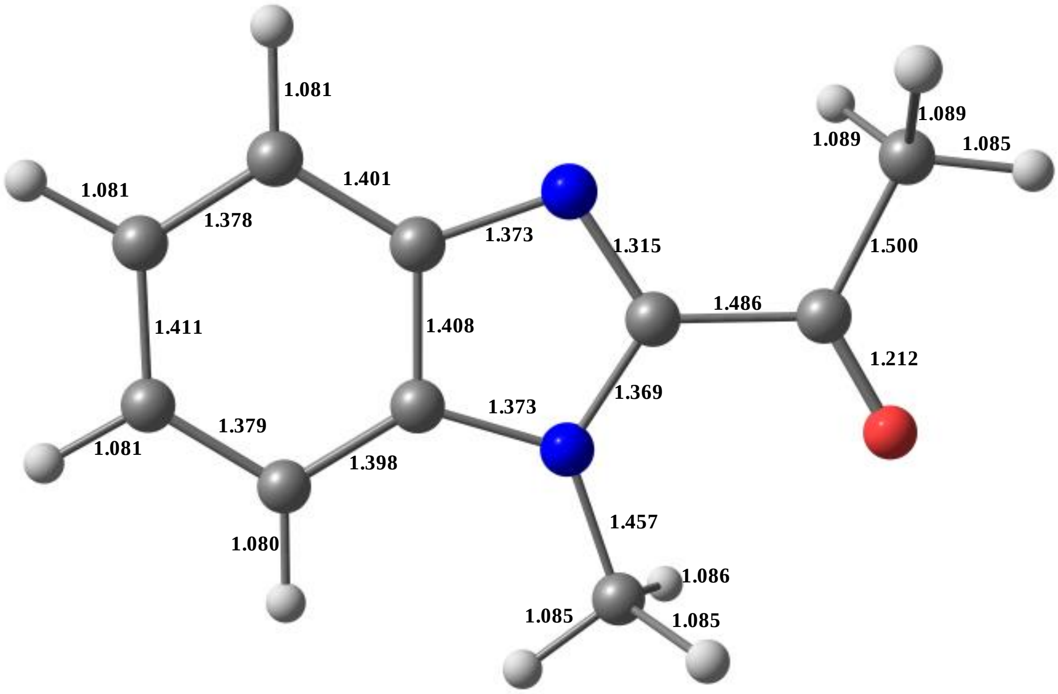

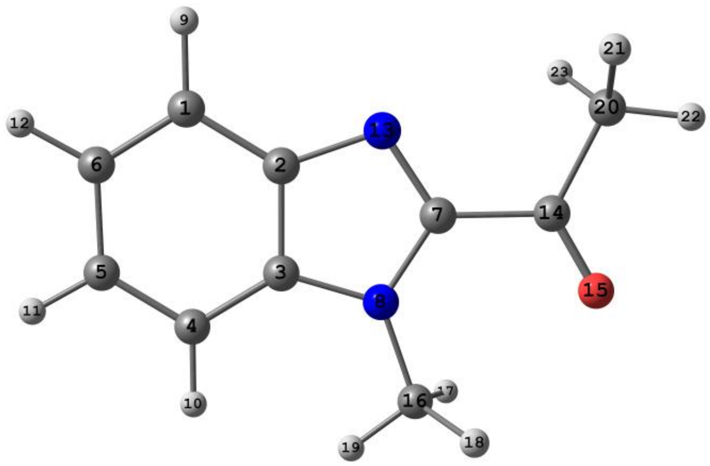

Summary of Natural Population Analysis:

Atom	No	Natural Charge	Core	Valence	Rydberg	Total
C	1	-0.19626	1.99902	4.17789	0.01934	6.19626
C	2	0.10871	1.99890	3.86706	0.02533	5.89129
C	3	0.16365	1.99890	3.81919	0.01825	5.83635
C	4	-0.23705	1.99903	4.22027	0.01775	6.23705
C	5	-0.19336	1.99917	4.17452	0.01968	6.19336
C	6	-0.22244	1.99915	4.20329	0.01999	6.22244
C	7	0.33261	1.99907	3.64016	0.02816	5.66739
N	8	-0.40250	1.99913	5.38532	0.01805	7.40250
H	9	0.21909	0.00000	0.77884	0.00207	0.78091
H	10	0.22161	0.00000	0.77639	0.00200	0.77839
H	11	0.21308	0.00000	0.78536	0.00156	0.78692
H	12	0.21269	0.00000	0.78572	0.00158	0.78731
N	13	-0.51708	1.99937	5.48374	0.03397	7.51708
C	14	0.58144	1.99926	3.38864	0.03065	5.41856
O	15	-0.61153	1.99975	6.58328	0.02849	8.61153
C	16	-0.34685	1.99930	4.33398	0.01356	6.34685
H	17	0.21694	0.00000	0.78151	0.00155	0.78306
H	18	0.21845	0.00000	0.78001	0.00154	0.78155
H	19	0.20711	0.00000	0.79135	0.00154	0.79289
C	20	-0.67010	1.99924	4.65889	0.01197	6.67010
H	21	0.23988	0.00000	0.75841	0.00171	0.76012
H	22	0.22172	0.00000	0.77618	0.00210	0.77828
H	23	0.24019	0.00000	0.75810	0.00171	0.75981
* Total *		0.00000	25.98932	65.70811	0.30257	92.00000

Natural Population spans Core, Valence, Rydberg, Total.

IX q=2

Energy **E= -572.586361722 h, G^{298}= -572.41236 h, μ=10.7 D**

Cartesian coordinates, Å

N	atom	x	y	z
1	C	-2.215999	-1.606015	0.000046
2	C	-1.019142	-0.876566	-0.000157
3	C	-0.996784	0.534877	-0.000140
4	C	-2.186162	1.281601	-0.000048
5	C	-3.347958	0.559966	0.000157
6	C	-3.364235	-0.864126	0.000232
7	C	1.075244	-0.192718	-0.000274
8	N	0.312819	0.918664	-0.000092
9	H	-2.217699	-2.684887	0.000037
10	H	-2.190719	2.359594	-0.000162
11	H	-4.291776	1.084735	0.000279
12	H	-4.319330	-1.368267	0.000431
13	N	0.275715	-1.274519	-0.000263
14	C	2.506845	-0.279148	0.000032
15	O	3.122778	0.830108	0.000125
16	C	0.778008	2.312842	0.000014
17	H	1.368170	2.493032	0.892328
18	H	1.366649	2.493666	-0.893179
19	H	-0.097293	2.948725	0.000961
20	H	0.592674	-2.236631	-0.000816
21	H	4.093590	0.741852	0.000355
22	C	3.193154	-1.578109	0.000236
23	H	2.893445	-2.144942	-0.884934
24	H	4.270230	-1.447066	0.000831
25	H	2.892281	-2.145310	0.884762

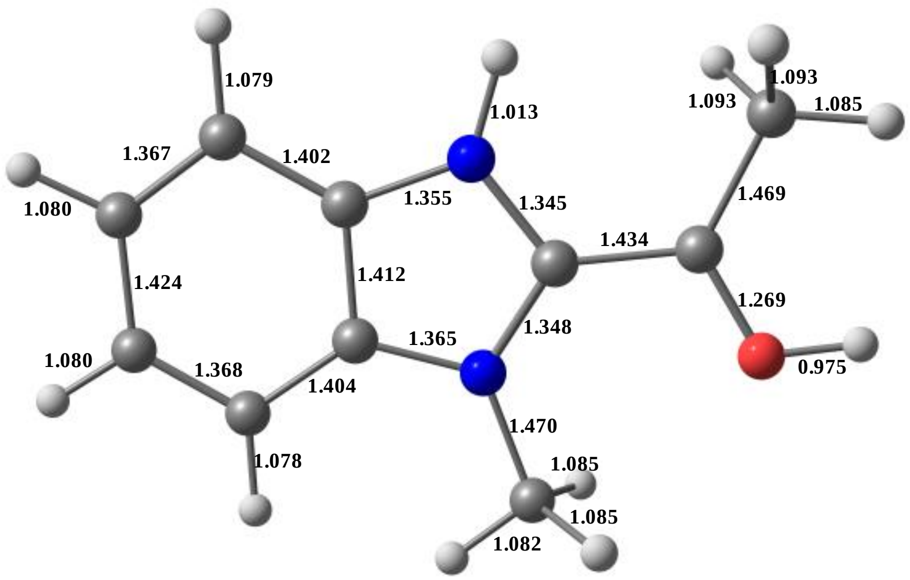

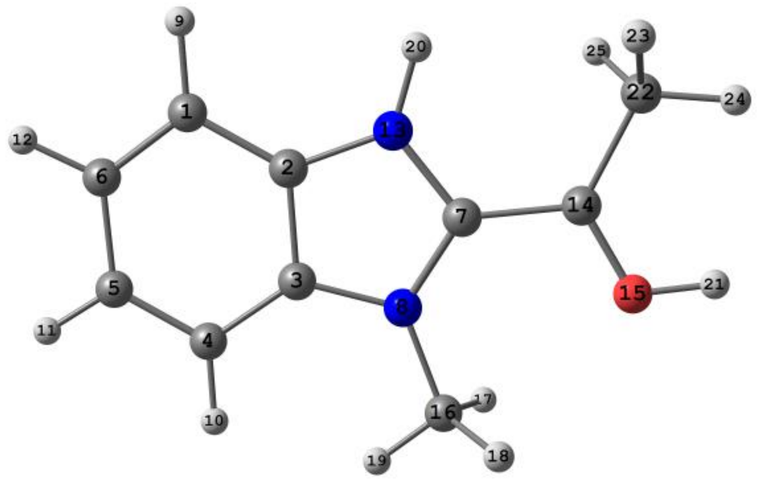

Summary of Natural Population Analysis:

Atom	No	Natural Charge	Core	Valence	Rydberg	Total
C	1	-0.20283	1.99902	4.18579	0.01802	6.20283
C	2	0.21251	1.99897	3.76808	0.02043	5.78749
C	3	0.22062	1.99894	3.76203	0.01841	5.77938
C	4	-0.20876	1.99902	4.19226	0.01748	6.20876
C	5	-0.13120	1.99916	4.11330	0.01873	6.13120
C	6	-0.12970	1.99916	4.11179	0.01875	6.12970
C	7	0.35218	1.99907	3.62508	0.02368	5.64782
N	8	-0.29692	1.99912	5.28228	0.01552	7.29692
H	9	0.25100	0.00000	0.74732	0.00168	0.74900
H	10	0.24678	0.00000	0.75152	0.00170	0.75322
H	11	0.23507	0.00000	0.76351	0.00142	0.76493
H	12	0.23500	0.00000	0.76359	0.00141	0.76500
N	13	-0.44369	1.99919	5.43029	0.01421	7.44369
C	14	0.65378	1.99912	3.32552	0.02159	5.34622
O	15	-0.55274	1.99966	6.52681	0.02627	8.55274
C	16	-0.35859	1.99927	4.34506	0.01427	6.35859
H	17	0.24458	0.00000	0.75419	0.00124	0.75542
H	18	0.24458	0.00000	0.75418	0.00124	0.75542
H	19	0.23294	0.00000	0.76574	0.00132	0.76706
H	20	0.48090	0.00000	0.51667	0.00243	0.51910
H	21	0.56071	0.00000	0.43676	0.00253	0.43929
C	22	-0.69081	1.99920	4.67786	0.01375	6.69081
H	23	0.29035	0.00000	0.70829	0.00136	0.70965
H	24	0.26394	0.00000	0.73420	0.00186	0.73606
H	25	0.29030	0.00000	0.70833	0.00136	0.70970
* Total *		2.00000	25.98890	65.75044	0.26066	92.00000

Core, Valence, Rydberg and Total are grouped under "Natural Population".
